# Digital outcome measures from smartwatch data relate to non-motor features of Parkinson’s disease

**DOI:** 10.1038/s41531-024-00719-w

**Published:** 2024-05-29

**Authors:** Ann-Kathrin Schalkamp, Neil A. Harrison, Kathryn J. Peall, Cynthia Sandor

**Affiliations:** 1 Division of Psychological Medicine and Clinical Neuroscience, School of Medicine, Cardiff University, Cardiff, United Kingdom; 2 UK Dementia Research Institute, Cardiff University, Cardiff, United Kingdom; 3 Division of Psychological Medicine and Clinical Neurosciences, Neuroscience and Mental Health Innovation Institute, Cardiff, United Kingdom; 4 Cardiff University Brain Research Imaging Centre (CUBRIC), Cardiff, United Kingdom; 5 Neuroscience and Mental Health Innovation Institute, Cardiff University, Cardiff, United Kingdom; 6 Present Address: Division of Brain Sciences, Faculty of Medicine, Imperial College London, London, United Kingdom

**Keywords:** Parkinson's disease, Predictive markers

## Abstract

Monitoring of Parkinson’s disease (PD) has seen substantial improvement over recent years as digital sensors enable a passive and continuous collection of information in the home environment. However, the primary focus of this work has been motor symptoms, with little focus on the non-motor aspects of the disease. To address this, we combined longitudinal clinical non-motor assessment data and digital multi-sensor data from the Verily Study Watch for 149 participants from the Parkinson’s Progression Monitoring Initiative (PPMI) cohort with a diagnosis of PD. We show that digitally collected physical activity and sleep measures significantly relate to clinical non-motor assessments of cognitive, autonomic, and daily living impairment. However, the poor predictive performance we observed, highlights the need for better targeted digital outcome measures to enable monitoring of non-motor symptoms.

## Introduction

Though classified as a motor disorder, Parkinson’s disease (PD) is associated with multiple non-motor symptoms that frequently arise prior to clinical diagnosis and progress throughout the disease course^1,2^. Non-motor symptoms include psychiatric symptoms, autonomic and sleep disturbance, pain, fatigue, and cognitive impairment, with many recognised to impact quality of life to a greater extent than motor symptoms^1,3^. Monitoring of these symptoms through in-clinic visits that use self-report and clinical rating scales poses limitations such as the time and cost of in-person visits, subjectivity of self-report, inter-rater variability, and assessments made in a clinical environment, rather than a ‘real life’ setting^4^.

Advances in digital heath technologies (DHTs), which are defined as technologies remotely acquiring information on health^5^, have enabled the transition of this monitoring to the at-home setting, overcoming several of the limitations outlined above. DHTs have already been developed to track motor signs and symptoms of PD with the digital scores developed providing a good representation of the existing gold standard clinical rating scale, the Unified Parkinson’s Disease Rating Scale (UPDRS)^6–10^. By contrast, with the exception of sleep, non-motor symptoms have been largely neglected in the context of DHTs^4,11^. Digital outcome measures for sleep length, quality, and stage exist^12^, however, are rarely used for the monitoring of PD. van Wamelen et al.^4^ identified 18 studies that use digital sensors to measure non-motor aspects of PD. Most studies related clinical examinations to sleep or activity measures extracted from accelerometers. They concluded that these studies show the potential of wearables for the assessment of non-motor symptoms but that their translation to clinical practice is far behind that of systems monitoring motor symptoms. Studies reporting on associations between digitally tracked motor symptoms and non-motor aspects of the disease found a link between digitally tracked bradykinesia and disturbed sleep^13^ and a relationship between digitally tracked bradykinesia and constipation^14^.

Here, we used rich multi-modal data from the Parkinson’s disease Progression Marker Initiative (PPMI) cohort to investigate how standard digital outcome measures of physical activity, sleep, and vital signs obtained from passively collected free-living smartwatch data relate to clinically assessed non-motor signs and symptoms and evaluated their potential utility in the context of clinical care.

## Results

### Digital weekly averages in the PPMI cohort

**Fig. 1:**
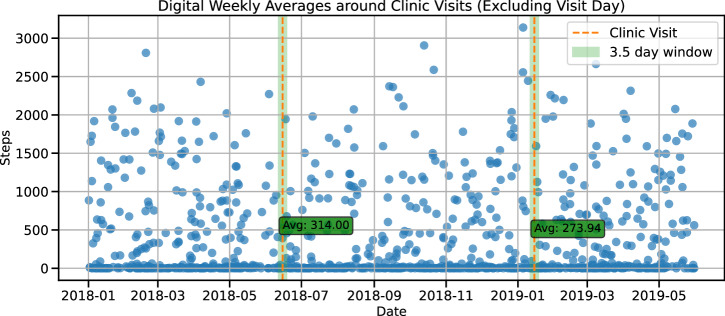
Deriving digital weekly averages. The schematic plot displays the data collection timeline using the example of hourly step count on simulated data. The continuously collected digital data was aligned with the visits to the clinic. An average over 6 days for each of the 14 digital outcome measures around the clinic visit was calculated using a 3.5-day window either side of the clinic visit, excluding the day of the clinic visit itself.

At the time of data retrieval (November 2022), the PPMI dataset provided a mean of 485 days of at home monitoring for 14 digital outcome measures describing physical activity (step count, walking minutes), sleep (total time, Rapid Eye Movement (REM) time, Non-REM (NREM) time, deep NREM time, light NREM time, wake after sleep onset (WASO), awakenings, sleep efficiency), and vital signs (pulse rate, mean root mean squared successive differences (RMSSD) (heart beat), median RMSSD, RMSSD variance) for 149 participants with a diagnosis of PD. Clinical data including assessments of cognitive performance, autonomic functioning, psychiatric symptoms, impairment in daily living, and motor symptoms was collected between 2010 and 2021^15^. 85 subjects had clinic visits while the digital data was collected with a mean of 1.58 ± 0.78 clinic visits per participant during this period. We computed digital weekly averages as the temporal mean over a 3.5-day window around the clinic visit, excluding the visit itself (Fig. 1). Analyses using a 30-day window are reported in the Supplementary Material (Supplementary Fig. 1). In the selected 6 days, the missingness of the digital data was high, ranging between 78.04% to 98.85%, where sleep features had the highest missingness due to them only being recorded during sleep hours thus biasing the missingness computation (Supplementary Table 1). PD cases were recruited no more than 2 years after their initial PD diagnosis such that 6.81 ± 2.11 years had passed since diagnosis at their respective last available visit to the clinic, coinciding with the digital data collection, leading to a cohort of individuals diagnosed with PD with an average age of 67.69 ± 7.54.

### Digital weekly averages capture variability in motor and non-motor clinical assessments

We investigated whether the weekly averages of each digital measure correspond to the clinical measures obtained at the respective clinic visit of that week.

**Fig. 2:**
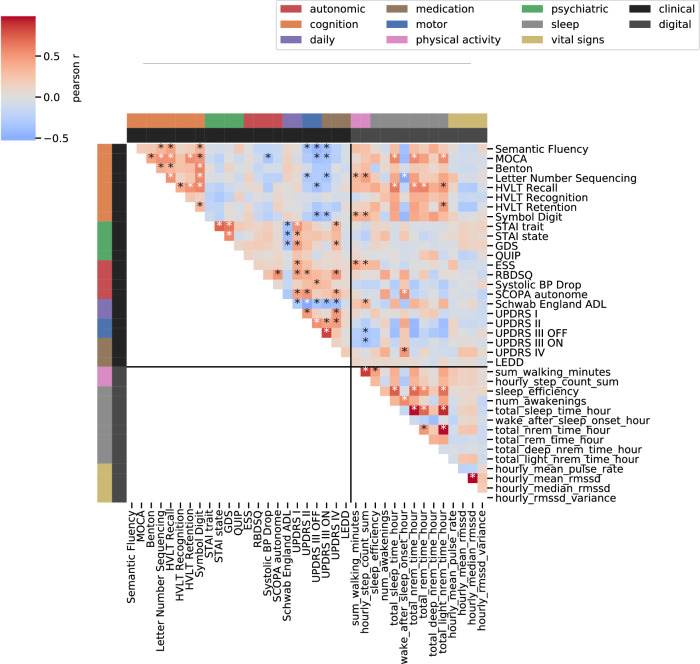
Correlation of digital weekly averages and clinical assessments. The heatmap displays the Pearson’s r coefficient for each digital weekly average and clinical assessment in the Parkinson’s disease group. If multiple visits overlapped with the digital data per person, the last visit to the clinic was chosen. Individual tests are grouped into modalities as indicated by the colours on the left and top. Asterisks indicate significant correlation *p* < 0.05 after FDR correction. ESS Epworth Sleepiness Scale, RBDSQ REM behavioural sleep disorder screening questionnaire, BP blood pressure, SCOPA Scale for Outcomes in Parkinson’s disease for Autonomic Symptoms, MOCA Montreal Cognitive Assessment, HVLT Hopkins Verbal Learning Test, STAI state-trait anxiety index, GDS Geriatric Depression Scale, QUIP Impulsive-Compulsive Disorders in PD, ADL activities of daily living, UPDRS Universal Parkinson’s Disease Rating Scale, LEDD Levodopa equivalent daily dosage, RMSSD root mean squared successive differences.

Clinical assessments themselves showed several associations within and between domains. Multiple groups of clinical non-motor symptoms demonstrated relatedness within their domains including cognitive (17 of 28 pairs reached significance after FDR correction), psychiatric (3 of 6), autonomic (1 of 6), and daily functioning (1 of 1) (Fig. 2, Supplementary Table 2). Several of the clinical non-motor signs and symptoms related to higher motor impairment (UPDRS II): reduced cognitive performance (2 of 8), increased RBDSQ (*p*-value = 9.6 × 10^−3^), reduced independence in daily functioning (2 of 2), and higher UPDRS IV (*p*-value = 1.34 × 10^−4^). The clinical non-motor assessments were further related to independence in daily living and UPDRS IV. Psychiatric assessment demonstrated correlation with increased difficulties in daily living (6 of 8) and with increased UPDRS IV (GDS: *p*-value = 4.72 × 10^−2^, STAI trait: *p*-value = 1.93 × 10^−2^). Higher levels of autonomic dysfunction were shown to be associated with increased difficulty in daily living and SCOPA autonome (*p*-value = 8.01 × 10^−3^) and RBDSQ (*p*-value = 1.54 × 10^−4^) were additionally related to UPDRS IV. Few relationships to cognitive performance were identified with only higher MoCA scores correlating with smaller drop in systolic blood pressure (*p*-value = 3.78 × 10^−2^).

There was also evidence of interrelatedness within digital outcome measures including sleep (10 of 28), vital signs (1 of 3), and physical activity (1 of 1), although there was no evidence of a relationship between the domains.

The weekly averages of the digital outcome measures (a 3.5-day window spanning the clinical visit date, Fig. 1) correlated with several of the non-motor clinical measures (Fig. 2, Supplementary Table 2). Of the eight cognitive measures, two were represented by the digital outcome measure for step count (LNS: *p*-value = 6.35 × 10^−4^, and symbol digit: *p*-value = 8.64 × 10^−3^) and four related to digital sleep measures; MoCA (*p*-value = 7.64 × 10^−3^) and HVTL recall (*p*-value = 2.07 × 10^−3^) were represented by total sleep time, LNS was negatively related to WASO (*p*-value = 1.93 × 10^−2^) and HVLT retention was positively related to light NREM sleep time (*p*-value = 4.37 × 10^−2^). Of the four autonomic functioning measures, two were captured by digital outcome measures; SCOPA autonome was represented by WASO (*p*-value = 1.14 × 10^−2^) and ESS by both physical activity measures (*p*-value < 4.64 × 10^−2^). One of the two daily living questionnaires was captured through the digitally measured step count (Schwab England ADL: *p*-value = 8.01 × 10^−3^). High UPDRS IV scores were associated with increased WASO (*p*-value = 2.09 × 10^−2^). None of the psychiatric measures were captured by any of the digital timeseries components. A link between the digital and clinical motor measures was also observed with UPDRS III OFF being negatively related to step count (*p*-value = 3.54 × 10^−2^).

### Digital averages could not predict non-motor assessments on an individual level

Having shown associations between digital and clinical measures, we sought to determine whether digital outcome measures could predict clinical scores. We compared linear regression models based on the 14 weekly digital averages to baseline models using only age at diagnosis, time since diagnosis, and sex as features.

**Fig. 3:**
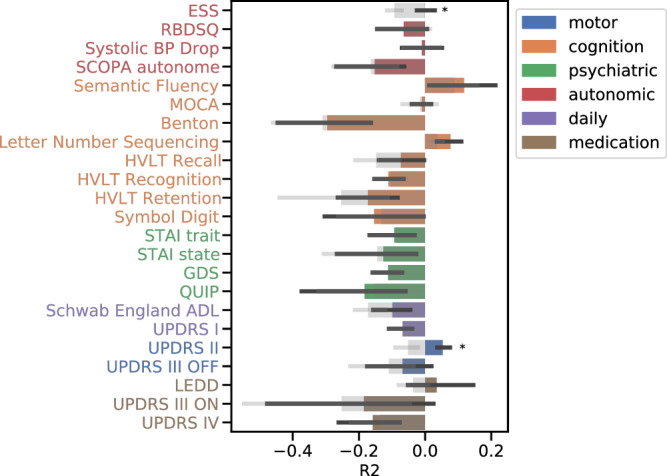
Digital outcome measures fail to predict clinical scores. The predictive performance (x-axis) of the 14 digital weekly averages (step count, walking minutes, NREM sleep time, deep NREM sleep time, light NREM sleep time, REM sleep time, sleep efficiency, number of awakenings, wake after sleep onset, total sleep time, mean pulse rate, mean RMSSD, median RMSSD, RMSSD variance) is shown for each clinical measure (y-axis) as the mean R2 across the five outer cross-validation test sets with their 95% Confidence Interval. The grey bars show the respective baseline model performance also with 95% CI. An asterisk indicates significant improvement over baseline at 0.05 significance (two-sided t-test with *N* = 5). The colour indicates the domain of the clinical measure. ESS Epworth Sleepiness Scale, RBDSQ REM behavioural sleep disorder screening questionnaire, BP blood pressure, SCOPA Scale for Outcomes in Parkinson’s disease for Autonomic Symptoms, MOCA Montreal Cognitive Assessment, HVLT Hopkins Verbal Learning Test, STAI state-trait anxiety index, GDS Geriatric Depression Scale, QUIP Impulsive-Compulsive Disorders in PD, ADL activities of daily living, UPDRS Universal Parkinson’s Disease Rating Scale, LEDD Levodopa equivalent daily dosage, RMSSD root mean squared successive differences.

Digital weekly averages could not predict the scores of standardised non-motor symptom questionnaires or rating scales used during the clinical assessments, on an individual level (Fig. 3, Supplementary Table 3). Most models achieved an R2 below 0, indicating that no variation in the clinical data could be explained through the digital outcome measures. Of note, the clinical motor measures were also not explained through the digital weekly averages. UPDRS II (R2 = 0.05 ± 0.02, *p*-value = 1.83 × 10^−3^) and ESS (R2 = 0 ± 0.04, *p*-value = 1.9 × 10^−3^) were the only measures that showed a significant improvement in performance compared to baseline models. UPDRS II being a self-report of motor impairments, digital measures being capable of capturing this indicates the general capabilities of digital measures to indicate motor symptom severity.

### Change in clinical non-motor scores over time relate to digital outcome measures

We investigated the sensitivity of the digital and clinical measures to detect change over time. We further assessed the relationship between changes in digital measures to that of the clinical measures with two approaches. First, we computed the difference between visits for the clinical scores and the associated weekly averages. Second, we extracted progression estimates from the whole observation time via linear mixed models for clinical scores and an automatic timeseries feature extraction method, tsfresh, for the digital timeseries.

We computed the difference of the clinical scores as well as the digital weekly averages between subsequent clinical visits during digital data collection (*N* = 35). Cohen’s d indicating the sensitivity to change detected showed the highest value for digitally measured total REM sleep time (0.63) (Supplementary Table 4). The second highest Cohen’s d value was observed for a cognitive measure (HVLT recall: 0.44). On average, the digital outcome measures were significantly better at detecting change than clinical ones (t = −2.79, *p*-value = 8.53 × 10^−3^, dof = 35, 95% CI = [−0.2–−0.03]). The clinic visits were on average 0.63 ± 0.22 years apart after an average of 5.55 ± 2.31 years post-diagnosis, potentially limiting the amount of change occurring.

The rate of change between visits revealed no association amongst the clinical measures indicating poor reliability in this data subset (Supplementary Fig. 2, Supplementary Table 5). By contrast, the rate of change of the digital outcome measures was observed to be related within domains, however, this was anticipated as they derive from one another. No significant associations between the rate of change of the clinical and digital measures were identified.

**Fig. 4:**
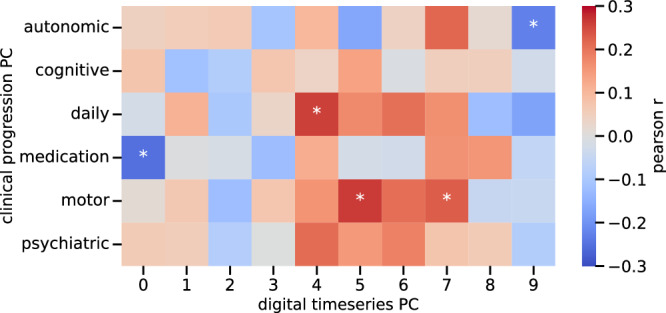
Correlation of digital and clinical progression. Associations between clinical progression and digital timeseries features are shown. The heatmap displays the Pearson’s r coefficient for the first 10 digital progression principal components from the features extracted with tsfresh and the clinical principal components for progression (the slope from the linear mixed models). Asterisk indicates significant correlation after 0.05 Bonferroni-correction.

Our second approach for assessing progression over time considered the whole observation time, meaning 5.91 ± 2.77 years for the clinical measures and 1.25 ± 0.54 years for the digital ones. This showed significant associations for motor and non-motor assessments, including impairments in daily living (*p*-value = 2.52 × 10^−3^), autonomic functioning (*p*-value = 8.23 × 10^−3^), and medication (*p*-value = 4.17 × 10^−3^) (Fig. 4, Supplementary Table 6). Motor progression was also related to digital timeseries components.

## Discussion

Here, we provide the first demonstration of how digital outcome measures collected in free-living conditions may relate to non-motor clinical assessments in a PD cohort. More specifically, we show that digital measures capturing weekly averages of physical activity are concordant with clinical assessments of multiple non-motor symptoms including cognitive, autonomic, and daily functioning as well as motor signs and symptoms. However, digital measures demonstrated limited power in predicting clinical scores on an individual level. The rate of progression of clinical measures showed associations with digital timeseries features when assessed over the whole disease course.

While PD is primarily viewed as a motor disorder with symptomatic treatment focussing on relieve of such motor aspects, non-motor aspects of the disease can precede the onset of motor symptoms by several years and throughout the disease course can dominate the clinical presentation^2^. Although DHTs have proven successful in the passive and continuous monitoring of the motor symptoms of PD, few studies have sought to examine whether digital measures may also aid in monitoring and longitudinal understanding of the non-motor symptoms despite the recognition that this latter symptom group has the greater impact on quality of life in PD^3,16^. Monitoring non-motor symptoms and assessing their progression is an important area of research which has thus far been neglected. We address this by investigating the potential of smartwatch data to inform non-motor symptoms. However, predictive performance on an individual level remains poor.

Non-motor symptoms are in themselves difficult to objectively measure with clinical scales^17^ thus evaluating the digital outcome measures against these scores might not be optimal. The digital tools may capture real-life impairments, not reflected in the clinical rating scales. The sensors used in this study and the derived clinical outcome measures were largely focussed on movement-based data, thus limited in capturing some of the non-motor aspects. More tailored sensors and derived outcome measures such as frequency of bathroom usage to potentially determine changes in urinary habits^18^ or frequency of text messages and phone calls to inform depressive symptoms^19^ may better reflect the non-motor symptoms. Such technologies still need to be explored in PD research. However, we have demonstrated here that standard measures from smartwatches are associated with cognitive, daily living, and autonomic impairment as assessed with clinical scales.

Previous studies have indicated the potential of DHTs in monitoring non-motor symptoms in PD with van Wamelen et al.^14^ reporting an association between digital bradykinesia scores and constipation. We found relations between step count and cognition in PD that were previously reported in other disorder like Schizophrenia^20^ or subacute stroke^21^. Our results further replicated the findings of digitally tracked poor sleep being related to worse cognitive performance in PD^22^ and self-reported sleep disturbances being associated with more PD treatment/motor complications^23,24^, which we identified with digitally measured increased WASO being related to increased UPDRS IV.

Our digital weekly averages were unable to predict clinical non-motor measures on an individual level. Although previous research has shown that motor symptoms of PD (as captured with UPDRS) can be predicted from digital outcome measures^7,10^, digital sensor data collected in free-living conditions showed only few associations with the clinical motor severity scales^25,26^. Our digital weekly averages of standard smartwatch measures could predict UPDRS II better than a baseline model but showed only low explained variance. It is likely that the standard digital measures considered here lack the specificity needed to accurately represent the clinical measures as they only describe high-level features of physical activity, sleep behaviour, and vital signs. More specific digital outcome measures collected passively in real-life settings, such as those identified for tremor or dyskinesia^7^, are currently lacking for the non-motor aspects of PD.

Limitations of this study relate primarily to data availability. Due to the Verily Study Watch only being introduced to the PPMI study 10 years after it began, overlapping clinical assessment and digital weekly average data was only available in 85 participants. Furthermore, at the time of digital monitoring these patients had already had a diagnosis of PD for about six years and had received dopaminergic therapy. The sample size was even smaller for the rate of change analysis where at least two visits per person had to overlap with the digital data collection leading to only 35 subjects being considered in this analysis. The computation of the digital weekly averages did not correct for missing data and could thus be biased by patient’s wearing patterns. Finally, our analysis was limited by only considering standard digital measures as provided by Verily, the code for which is proprietary, and therefore limiting the reproducibility of this work.

In conclusion, we have demonstrated associations between standard smartwatch digital measures and multiple non-motor aspects of PD including i) digitally measured sleep with cognition and motor complications due to medication, and ii) digitally measured physical activity with cognition, daytime sleepiness, and independence in daily living. Despite these associations, the clinical measures could not be predicted from digital data on an individual level, highlighting the need for more specialised digital outcome measures for the non-motor aspects of PD.

## Methods

All analyses were performed in python v3.9 using sklearn^27^ 1.2.1 for model training and evaluation, scipy 1.10.0 and pingouin^28^ 0.5.3 for statistical testing, and matplotlib 3.6.3 and seaborn 0.12.2 for creating figures. Data loading and manipulation has been facilitated through an adapted version of pypmi (https://github.com/rmarkello/pypmi). All code will be made available upon publication at https://github.com/aschalkamp/PPMI_nonmotor_digital.

### Study cohort

The Parkinson’s disease progression marker initiative (PPMI) has collected data from those with recently diagnosed (*denovo*) PD, people at risk, and unaffected controls since 2010. Participating PPMI sites all received approval from an ethical standards committee before study initiation and written informed consent was obtained for all individuals participating in the study. The study was registered at clinicaltrials.gov (NCT01141023). This analysis used data openly available from PPMI. They put a focus on longitudinal data collection of brain imaging, blood, urine, cerebrospinal fluid (CSF), and clinical assessment data. Since 2018 a subset of participants has been supplied with a Verily Study Watch, which is equipped with a multitude of sensors including accelerometer, gyroscope, electroencephalography (ECG), and photoplethysmography (PPG). We used the analytic dataset cohort assignment, which provides the most up-to-date assignment of subjects into PD, healthy control, Scans Without evidence of dopaminergic deficit (SWEDD), and prodromal class.

#### Digital data

From the raw Verily Study Watch data several derived measures are provided by Verily and were accessed by us through the LONI website of PPMI in November 2022. These include data on sleep, physical activity, and vital signs. Data was available between 2018 and 2020. Each derived digital measures had a different availability with the sleep measures being the scarcest due to them only being available for hours spent asleep. The hourly step count data covered an average of 1.25 years (std = 0.54) with a mean recorded time of 0.91 years (std = 0.52). The time between the measurements ranged from one hour to 6.76 days with a mean of 137.56 min (std = 530.76). The sleep data covered an average of 1.19 years (std = 0.57) with a mean covered time of 8.4 days (std = 6.68). Here, the time between measurements was larger on average with 6.35 days (std = 19.83).

### Clinical data

Data was downloaded from LONI PPMI in March 2021. The following clinical assessments were retrieved: motor assessments including UPDRS scores (part II, III), cognitive assessments like Montreal Cognitive Assessment (MoCA), semantic fluency, Benton judgement of line orientation, and WMS-III Letter-Number Sequencing Test (LNS), Hopkins Verbal Learning Test (HVLT), and symbol digit, psychiatric questionnaires including State-trait-anxiety inventory (STAI), geriatric depression score (GDS), and Impulsive-Compulsive Disorders in PD (QUIP), as well as autonomic assessments like Scale for Outcomes in Parkinson’s disease for Autonomic Symptoms (SCOPA), Epworth Sleepiness Score (ESS), REM sleep behaviour disorder screening questionnaire (RBDSQ), and blood pressure drop, and assessments of daily functioning with the modified Schwab England Activities of Daily Living (Schwab England ADL) and the UPDRS I. Information on medication was included as Levodopa equivalent daily dosage (LEDD) and UPDRS IV.

These data were collected, cleaned, and merged based on the subject identifier and visit date. For UPDRS III, we distinguished between ON and OFF assessments with OFF being those where the subject was not on medication either due to not taking medication or because the medication was deliberately not taken for this assessment, and ON being all assessments conducted when the subject took the normal medication.

### Temporal alignment

To align the smartwatch data with the clinic visit data, the date of digital data collection had to be inferred and was then merged with the date of the clinic visit. The local date for the derived digital measures was calculated based on the provided age in seconds, the weekday, and the local time. The age in seconds was transformed to a date using the date of birth. The weekday of this estimated date was compared to the provided weekday. If they did not match, the estimated date was shifted to the closest date which has the correct weekday. Using this estimated date, the digital data was merged to the clinical data. Due to the smartwatch study only being included later in the study, the overlap with the clinical examinations is limited. 85 participants with PD had an overlap of digital data and a clinic visit with a mean of 1.58 ± 0.78 clinical visits per person during digital data collection.

### Correlation of clinical measures and digital weekly averages

To relate digital and clinical measures to one another, we computed digital weekly averages from the digital timeseries data (Fig. 1). For this, the clinical visit date was used as the mid-week point around which a 3.5-days sized window of the digital data was averaged, removing the visit day itself due to it being a non-representative day including a visit to the clinic. Thus, a mean over 6 days of all available data was calculated. We restricted this analysis to only include each participant once to avoid overrepresentation of specific subjects by choosing the last available clinical visit with an overlapping digital recording available. We computed Pearson’s correlation between the digital averages and the clinical visit information with 0.05 FDR correction. Due to varying availability of clinical assessments, the number of subjects in each correlation differs (Supplementary Table 2).

### Prediction of clinical scores from digital weekly averages

To predict the clinical measures from the digital weekly averages, we built regression models using all digital weekly averages as predictors and diagnosis age, time since diagnosis, and male sex as covariates. To estimate the baseline performance, a model using only the covariates was built. All these models used an elastic net penalty with R2 score loss and were fitted with a nested five-fold cross-validation. In the inner split, gridsearch was applied to identify the best hyperparameters for the penalty, namely the L1 to L2 ratio and the alpha (strength of penalty) parameter. Performance was reported with mean and standard deviation of the R2 score across the five outer test folds.

### Rate of change analysis

To assess the sensitivity of the measures to detect change over time, we computed Cohen’s d in a paired test between the first two visits coinciding with digital data collection (*N* = 35). We further added Hedges g for comparison due to small sample sizes. We tested for differences between digital and clinical measures to detect change with a two-sided t-test on the Cohen’s d coefficients.

We further assessed the relationship between the measured change over time with two approaches, the first one limited to digital weekly averages and clinic visits during digital data collection, the second one considering the whole available data.

First, we computed the difference between consecutive visits scaled by the time between visits. 35 subjects had more than two overlaps of the digital data and a clinic visit. The mean over this change over time was correlated between clinical and digital data with 0.05 FDR correction.

Second, we evaluated the progression over the whole observation period. The clinical measures were transformed, if needed, such that higher scores represented more impairment. Random intercepts and slopes were fitted to allow for variation of the individuals around the population mean. The slope was interpreted as the speed of progression. We restricted the data for each modality individually to subjects with at least two visits to retain the most data possible. Overall, this data was available for 665 PD cases with a diagnosis age of 60.24 ± 10.10 years. They were followed for an average of 5.91 ± 2.77 years with 11.33 ± 4.66 visits which were on average 208 ± 130 days apart. We computed principal components via Principal Component Analysis (PCA) for the clinical intercepts and slopes separately for defined modalities: motor (UPDRS III OFF, UPDRS II), daily (Schwab England ADL, UPDRS I), cognitive (MoCA, Benton, LNS, HVTL recall, retention, and recognition, symbol digit, semantic fluency), psychological (STAI trait, STAI state, GDS, QUIP), autonomic (ESS, RBDSQ, SCOPA autonome, systolic blood pressure drop). The same method was used for the medication domain (UPDRS III ON, UPDRS III OFF, UPDRS IV, LEDD) but the time since first medication was modelled instead of time since diagnosis. The principal components were then identified based on UPDRS III ON-OFF, UPDRS IV, and LEDD. On average, the first principal component explained 0.76 ± 0.25 of the variance.

For the digital timeseries data, we used tsfresh. Tsfresh was applied for each individual for each digital feature to extract their timeseries features. This included 783 derived features like the maximum, minimum, skewness, kurtosis, and trend for each timeseries. The method does not consider the time intervals between data, leading to potential biases where data was only sporadically available. We ran PCA on all digital timeseries features from tsfresh. The first 10 principal components explained 27% of the total variance.

We aimed to study the correlation of the progression estimates of the different data modalities. The principal components of the clinical features were correlated with the principal components of the digital features via Pearson’s correlation (*N* = 135).

### Reporting summary

Further information on research design is available in the Nature Research Reporting Summary linked to this article.

### Supplementary information

Supplemental Material

Reporting summary

## Data Availability

This analysis used data openly available from PPMI. Data used in the preparation of this article were obtained in March 2021 with digital data downloaded in November 2022 from the Parkinson’s Progression Markers Initiative (PPMI) database (www.ppmi-info.org/access-data-specimens/download-data), RRID:SCR_006431. For up-to-date information on the study, visit www.ppmi-info.org.

## Code availability

All associated code to reproduce the analyses performed here will be made publicly available upon publication at https://github.com/aschalkamp/PPMI_nonmotor_digital.
