# Supplementary material for: Digital outcome measures from smartwatch data relate to non-motor features of Parkinson’s disease
Source: NPJ Parkinsons Dis. 2024 May 29;10:110. doi: 10.1038/s41531-024-00719-w (PMC11137004; doi:10.1038/s41531-024-00719-w)
Supplement: Supplementary file 1 — Supplemental Material [file 41531_2024_719_MOESM1_ESM.pdf]

Supplemental Material

Digital monthly averages

Instead of using a 3.5-day window around the clinic visit, we also evaluated a 30-day window. Due to the large, considered timeframe, more subjects and more visits were overlapping with digital data collection (N=116). Missingness was thus also further increased (84.1% - 99.53%). The monthly digital averages correlated with the clinical measures, revealing a similar pattern as the weekly digital averages (Supplemental Figure 1). Due to the increased sample size and thus power, more associations reached statistical significance at 0.05 FDR correction. Of note, two additional cognitive measures were associated with step count (Semantic Fluency, HVLT Recall). Again, half of the cognitive measures related to sleep measures. In this analysis, new associations with vital signs were revealed for Semantic Fluency and QUIP. Associations for ESS, UPDRS IV, and SCOPA autonome were not identified here. For the rate of change analysis, HVTL recall remained the most sensitive to change (Cohen's d = 0.5). Digital sleep measures remained the most sensitive digital ones and overall digital measures had slightly higher, but not significantly, sensitivity to change (clinical: 0.16, digital: 0.19, p-value = 0.41). Contrary to the digital weekly averages, the difference of the monthly averages did show associations with the difference between clinic visits (Supplemental Figure 3). Increases in UPDRS II related to decreases in light NREM sleep time, increases in UPDRS III ON related to increases in mean pulse rate, and increases in UPDRS IV related to decreases in walking minutes. Findings for this monthly analysis that were not significant in the weekly one, can again be attributed to the bigger sample size (mean N=26 vs mean N=10).

Supplemental Figure 1: Associations between digital monthly averages and clinical assessments

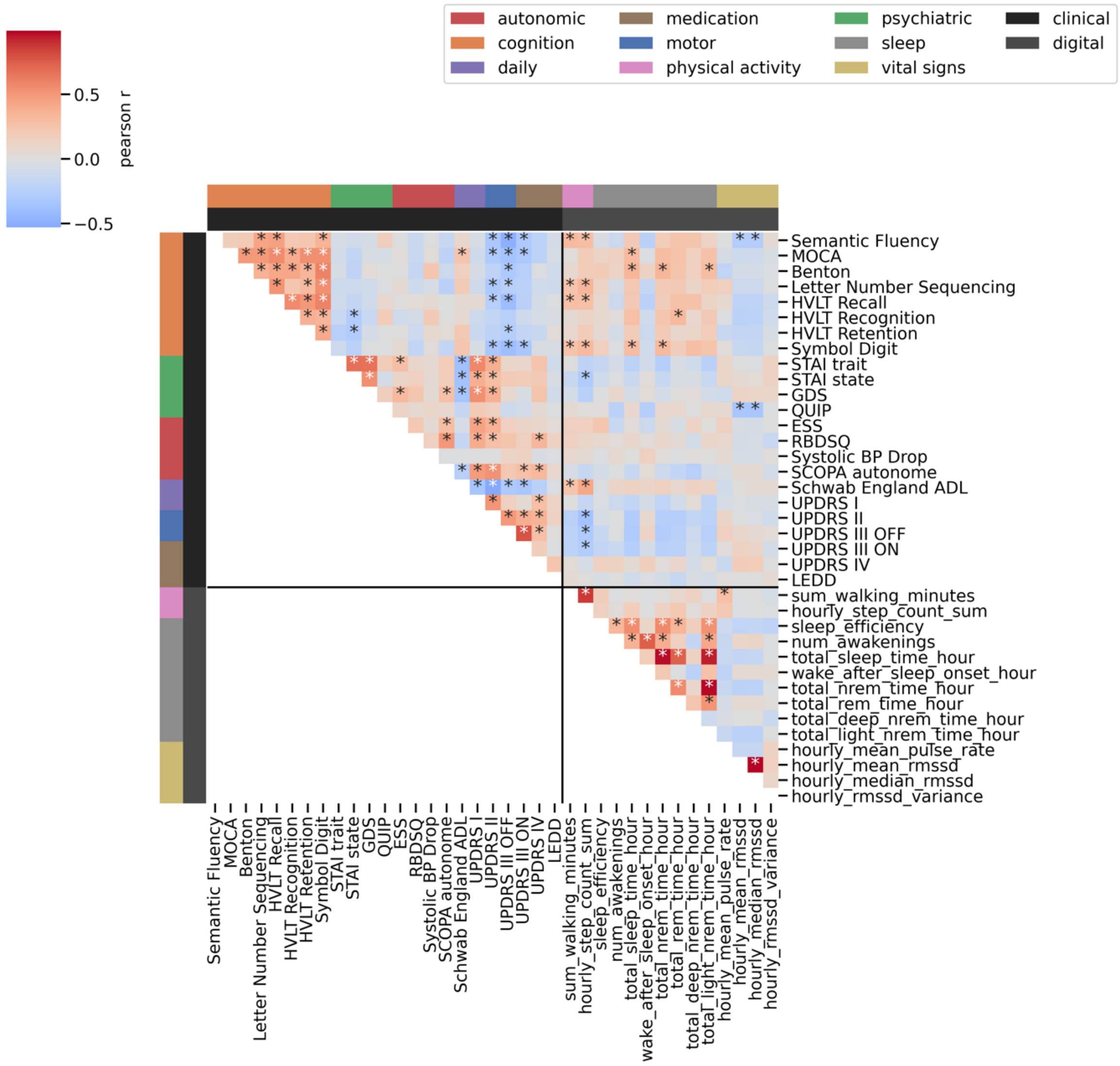

The heatmap displays the Pearson's r coefficient for each digital monthly average and clinical assessment in the Parkinson's disease group. If multiple visits overlapped with the digital data per person, the last visit to the clinic was chosen. Individual tests are grouped into modalities as indicated by the colours on the left and top. Asterisks indicate significant correlation after 0.05 FDR correction.

Supplemental Figure 2: Difference between visits is not related between digital weekly averages and clinical measures

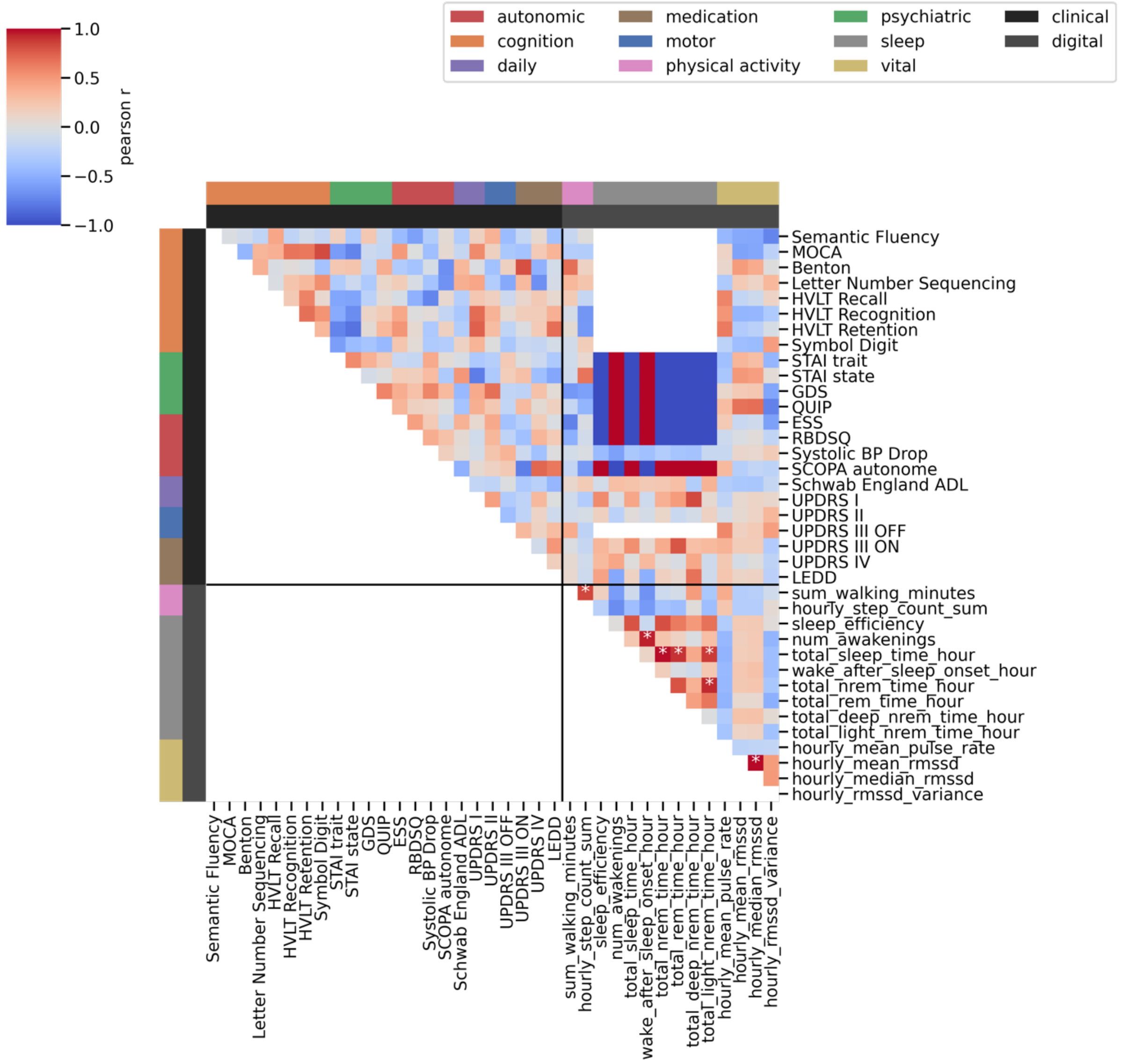

The heatmap displays for every combination of digital and clinical measures the correlation between the mean rate of change over time. The domains can be seen on the left and top coded by the colour bars. The colour indicates the Pearson r coefficient and an asterisk indicates 0.05 FDR corrected significance.

ESS: Epworth Sleepiness Scale, RBDSQ: REM behavioral sleep disorder screening questionnaire, BP: blood pressure, SCOPA: Scale for Outcomes in Parkinson's disease for Autonomic Symptoms, MOCA: Montreal Cognitive Assessment, HVLT: Hopkins Verbal Learning Test, STAI: state-trait anxiety index, GDS: Geriatric Depression Scale, QUIP: Impulsive-Compulsive Disorders in PD, ADL: activities of daily living, UPDRS: Universal Parkinson’s Disease Rating Scale, LEDD: Levodopa equivalent daily dosage, RMSSD: mean root mean squared successive differences

Supplemental Figure 3: Difference between visits is not related between digital monthly averages and clinical measures

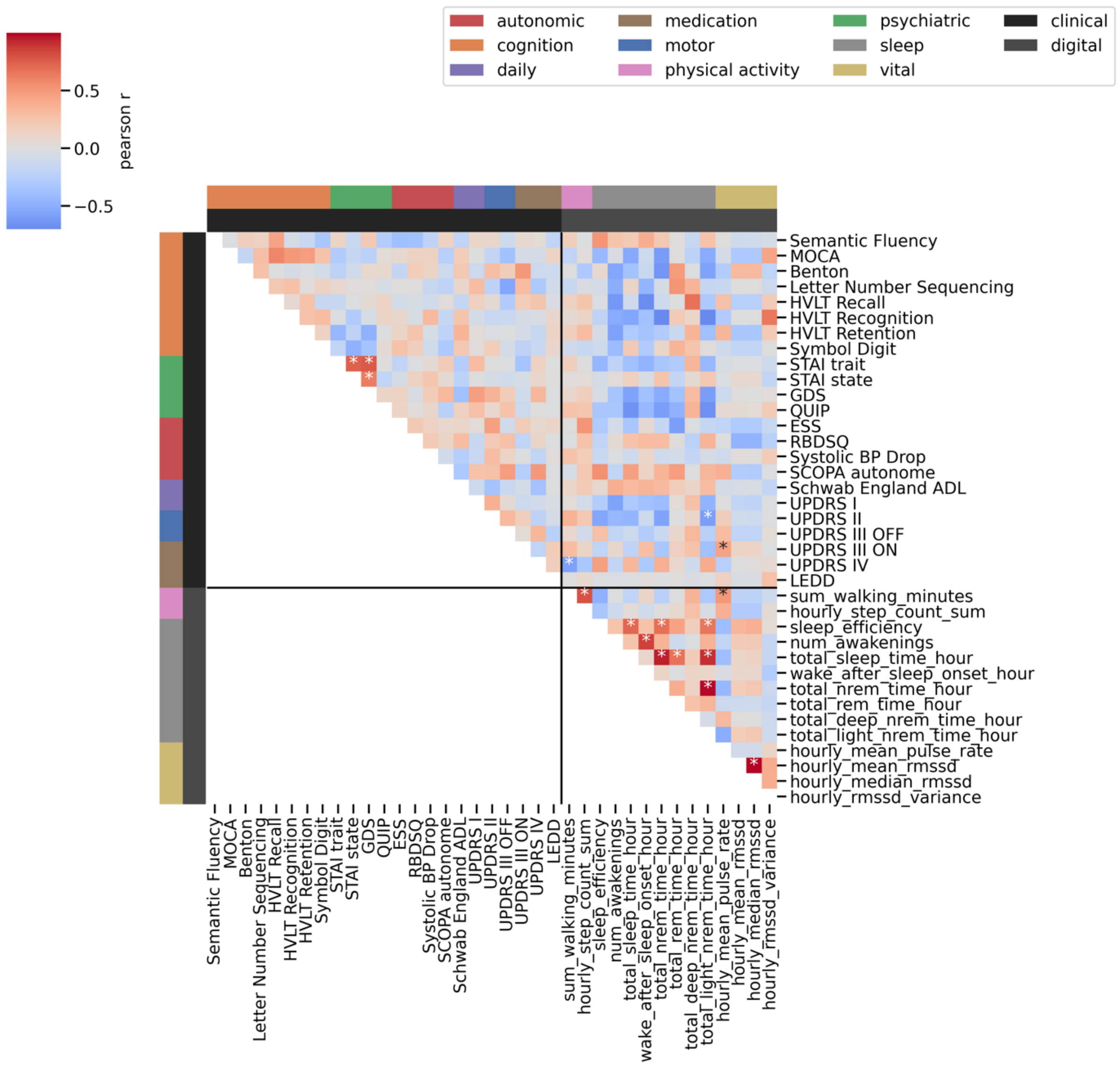

The heatmap displays for every combination of digital and clinical measures the correlation between the mean rate of change over time. The domains can be seen on the left and top coded by the colour bars. The colour indicates the Pearson r coefficient and an asterisk indicates 0.05 FDR corrected significance.

ESS: Epworth Sleepiness Scale, RBDSQ: REM behavioral sleep disorder screening questionnaire, BP: blood pressure, SCOPA: Scale for Outcomes in Parkinson's disease for Autonomic Symptoms, MOCA: Montreal Cognitive Assessment, HVLT: Hopkins Verbal Learning Test, STAI: state-trait anxiety index, GDS: Geriatric Depression Scale, QUIP: Impulsive-Compulsive Disorders in PD, ADL: activities

of daily living, UPDRS: Universal Parkinson's Disease Rating Scale, LEDD: Levodopa equivalent daily dosage, RMSSD: mean root mean squared successive differences

Supplemental Figure 4: Monthly digital outcome measures fail to predict clinical scores

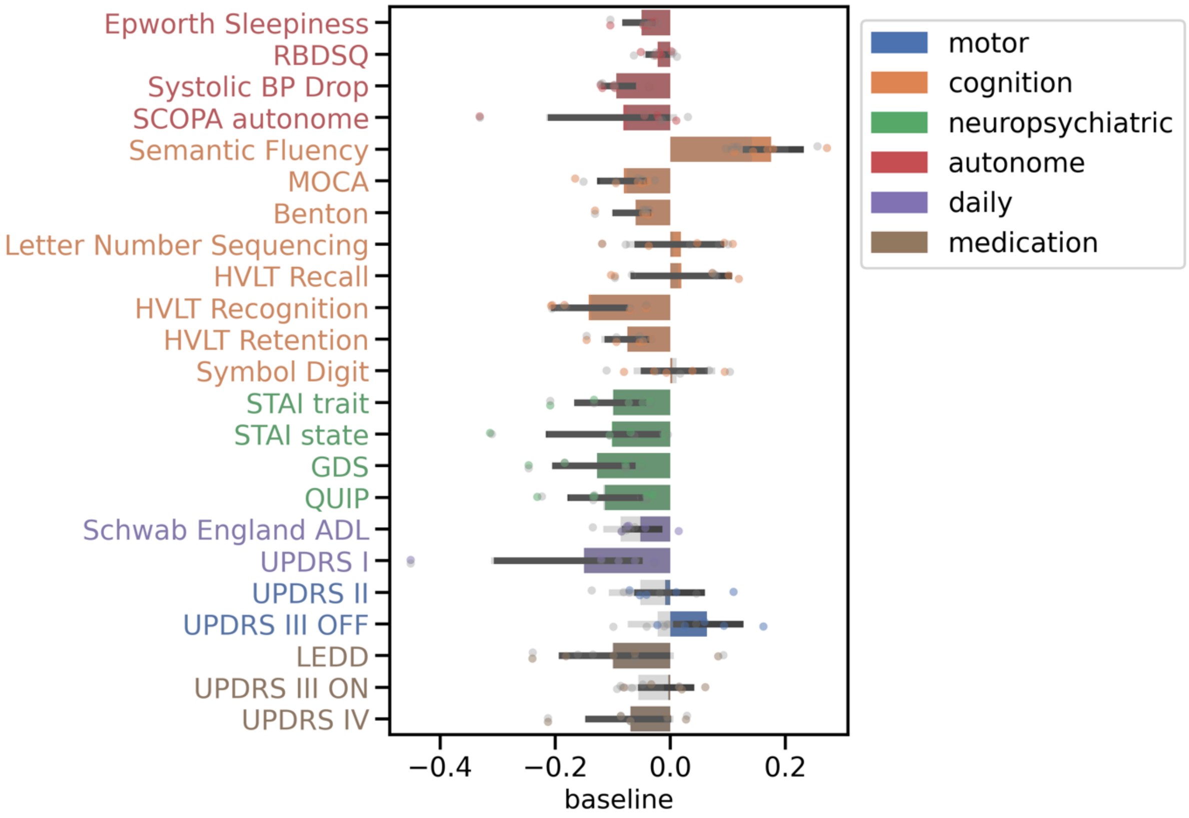

The predictive performance (x-axis) of the 14 monthly digital averages (step count, walking minutes, NREM sleep time, deep NREM sleep time, light NREM sleep time, REM sleep time, sleep efficiency, number of awakenings, wake after sleep onset, total sleep time, mean pulse rate, mean RMSSD, median RMSSD, RMSSD variance) for each clinical measure (y-axis) as the mean R2 across the five outer cross-validation test sets with their 95% Confidence Interval. The grey bars show the respective baseline performance also with 95% CI. The dots display the individual data points. An asterisk indicates significant improvement over baseline at 0.05 significance (two-sided t-test with N=5). The colour indicates the domain of the clinical measure.

ESS: Epworth Sleepiness Scale, RBDSQ: REM behavioral sleep disorder screening questionnaire, BP: blood pressure, SCOPA: Scale for Outcomes in Parkinson's disease for Autonomic Symptoms,

MOCA: Montreal Cognitive Assessment, HVLT: Hopkins Verbal Learning Test, STAI: state-trait anxiety index, GDS: Geriatric Depression Scale, QUIP: Impulsive-Compulsive Disorders in PD, ADL: activities of daily living, UPDRS: Universal Parkinson's Disease Rating Scale, LEDD: Levodopa equivalent daily dosage, RMSSD: mean root mean squared successive differences

Supplemental Table 1: Missingness of digital data

The percentage of missing data is shown for each digital outcome measure around the clinic visit. Missingness was computed as the difference to the expected hourly data rate for six days (24*6).

	count	mean
sum_walking_minutes	134	78.36339
hourly_step_count_sum	129	78.04156
sleep_efficiency	53	98.84696
num_awakenings	53	98.84696
total_sleep_time_hour	53	98.84696
wake_after_sleep_onset_hour	53	98.84696
total_nrem_time_hour	53	98.84696
total_rem_time_hour	53	98.84696
total_deep_nrem_time_hour	53	98.84696
total_light_nrem_time_hour	53	98.84696
hourly_mean_pulse_rate	129	78.07386
hourly_mean_rmssd	127	89.37008
hourly_median_rmssd	127	89.37008
hourly_rmssd_variance	123	92.46838

Supplemental Table 2: Association between digital weekly averages and clinical assessments

Results of the Pearson correlation analysis between clinical and digital markers. The Pearson's r coefficient, the associated p-value, the FDR corrected p-value, and the sample size is displayed.

f1	f2	N	pearson r	p-value	p-corrected FDR	sign
Semantic Fluency	MOCA	73	0.202439	0.085865	0.259126	
Semantic Fluency	Benton	74	0.226843	0.051947	0.177419	
Semantic Fluency	Letter Number Sequencing	74	0.334697	0.00356	0.031197	*
Semantic Fluency	HVLT Recall	74	0.447504	6.4E-05	0.001184	*
Semantic Fluency	HVLT Recognition	73	0.116517	0.326254	0.602213	
Semantic Fluency	HVLT Retention	74	0.187127	0.110387	0.30997	

Semantic Fluency	Symbol Digit	74	0.365282	0.001374	0.014521	*
Semantic Fluency	STAI trait	74	-0.03545	0.764284	0.883703	
Semantic Fluency	STAI state	74	-0.08188	0.488	0.742027	
Semantic Fluency	GDS	74	-0.04069	0.73068	0.870542	
Semantic Fluency	QUIP	74	0.214516	0.066453	0.215893	
Semantic Fluency	ESS	74	-0.01767	0.881224	0.93745	
Semantic Fluency	RBDSQ	74	-0.1761	0.133404	0.356815	
Semantic Fluency	Systolic BP Drop	74	-0.17757	0.13013	0.350877	
Semantic Fluency	SCOPA autonome	74	-0.15421	0.18958	0.464191	
Semantic Fluency	Schwab England ADL	74	0.113899	0.333914	0.605958	
Semantic Fluency	UPDRS I	74	0.009577	0.935457	0.963872	
Semantic Fluency	UPDRS II	74	-0.33247	0.003802	0.032888	*
Semantic Fluency	UPDRS III OFF	70	-0.43623	0.00016	0.002599	*
Semantic Fluency	UPDRS III ON	71	-0.36678	0.001655	0.017218	*
Semantic Fluency	UPDRS IV	72	-0.23568	0.046263	0.163888	
Semantic Fluency	LEDD	74	-0.05633	0.633583	0.825766	
Semantic Fluency	sum_walking_minutes	74	0.285853	0.013553	0.075223	
Semantic Fluency	hourly_step_count_sum	74	0.258508	0.026158	0.110963	
Semantic Fluency	sleep_efficiency	32	0.063703	0.729064	0.870173	
Semantic Fluency	num_awakenings	32	-0.09285	0.613266	0.811434	
Semantic Fluency	total_sleep_time_hour	32	0.290323	0.106981	0.304485	
Semantic Fluency	wake_after_sleep_onset_hour	32	-0.38001	0.031925	0.128086	
Semantic Fluency	total_nrem_time_hour	32	0.294865	0.101359	0.293499	
Semantic Fluency	total_rem_time_hour	32	0.198315	0.276569	0.549034	
Semantic Fluency	total_deep_nrem_time_hour	32	0.397034	0.024449	0.106997	
Semantic Fluency	total_light_nrem_time_hour	32	0.199179	0.274441	0.549034	
Semantic Fluency	hourly_mean_pulse_rate	74	0.06295	0.594153	0.810625	
Semantic Fluency	hourly_mean_rmssd	73	-0.12548	0.290163	0.566712	
Semantic Fluency	hourly_median_rmssd	73	-0.12449	0.294022	0.568101	
Semantic Fluency	hourly_rmssd_variance	72	0.11687	0.328233	0.602213	
MOCA	Benton	73	0.451103	6.19E-05	0.001178	*
MOCA	Letter Number Sequencing	73	0.529279	1.48E-06	4.94E-05	*
MOCA	HVLT Recall	73	0.525734	1.79E-06	5.41E-05	*
MOCA	HVLT Recognition	72	0.255907	0.030029	0.123451	
MOCA	HVLT Retention	73	0.54988	4.68E-07	2.08E-05	*
MOCA	Symbol Digit	73	0.506274	4.89E-06	0.000116	*
MOCA	STAI trait	73	-0.11881	0.316768	0.594274	
MOCA	STAI state	73	-0.21553	0.067059	0.216803	
MOCA	GDS	73	-0.00294	0.980292	0.986679	
MOCA	QUIP	73	-0.10752	0.365245	0.633471	
MOCA	ESS	73	0.045069	0.704975	0.859134	
MOCA	RBDSQ	73	-0.0932	0.432904	0.704924	
MOCA	Systolic BP Drop	73	-0.3264	0.004829	0.037837	*
MOCA	SCOPA autonome	73	-0.13545	0.253216	0.533677	
MOCA	Schwab England ADL	73	0.150944	0.202407	0.47836	
MOCA	UPDRS I	73	-0.06486	0.585655	0.803773	

MOCA	UPDRS II	73	-0.28795	0.0135	0.075223	
MOCA	UPDRS III OFF	69	-0.36741	0.001899	0.019161	*
MOCA	UPDRS III ON	70	-0.33822	0.004187	0.034424	*
MOCA	UPDRS IV	71	-0.24618	0.038503	0.14653	
MOCA	LEDD	73	-0.04741	0.69043	0.849525	
MOCA	sum_walking_minutes	73	0.080828	0.49664	0.751733	
MOCA	hourly_step_count_sum	73	0.229165	0.051149	0.176908	
MOCA	sleep_efficiency	32	0.328721	0.066214	0.215893	
MOCA	num_awakenings	32	0.079887	0.663835	0.843729	
MOCA	total_sleep_time_hour	32	0.575949	0.000562	0.007637	*
MOCA	wake_after_sleep_onset_hour	32	-0.4072	0.020719	0.095826	
MOCA	total_nrem_time_hour	32	0.558915	0.000884	0.010329	*
MOCA	total_rem_time_hour	32	0.455513	0.008797	0.05744	
MOCA	total_deep_nrem_time_hour	32	0.165725	0.364684	0.633471	
MOCA	total_light_nrem_time_hour	32	0.548928	0.00114	0.012451	*
MOCA	hourly_mean_pulse_rate	73	-0.05785	0.626876	0.820235	
MOCA	hourly_mean_rmssd	72	-0.08677	0.468625	0.729716	
MOCA	hourly_median_rmssd	72	-0.0667	0.577717	0.799916	
MOCA	hourly_rmssd_variance	71	-0.06295	0.60197	0.811434	
Benton	Letter Number Sequencing	74	0.388036	0.000635	0.008137	*
Benton	HVLT Recall	74	0.329893	0.0041	0.034424	*
Benton	HVLT Recognition	73	0.263101	0.024518	0.106997	
Benton	HVLT Retention	74	0.285298	0.013744	0.07565	
Benton	Symbol Digit	74	0.593503	2.51E-08	1.85E-06	*
Benton	STAI trait	74	0.029102	0.805577	0.890844	
Benton	STAI state	74	-0.15045	0.200726	0.47836	
Benton	GDS	74	0.121335	0.303101	0.580073	
Benton	QUIP	74	-0.03067	0.795328	0.890844	
Benton	ESS	74	0.028182	0.811608	0.894918	
Benton	RBDSQ	74	-0.16284	0.165688	0.427707	
Benton	Systolic BP Drop	74	-0.05767	0.625513	0.820235	
Benton	SCOPA autonome	74	-0.08057	0.494999	0.750955	
Benton	Schwab England ADL	74	0.174415	0.137213	0.365536	
Benton	UPDRS I	74	0.07538	0.523268	0.770548	
Benton	UPDRS II	74	-0.08931	0.449225	0.718752	
Benton	UPDRS III OFF	70	-0.29546	0.013022	0.074127	
Benton	UPDRS III ON	71	-0.29014	0.014113	0.077044	
Benton	UPDRS IV	72	-0.00958	0.936374	0.963872	
Benton	LEDD	74	-0.10806	0.359452	0.628522	
Benton	sum_walking_minutes	74	0.202089	0.084221	0.257485	
Benton	hourly_step_count_sum	74	0.303261	0.008627	0.05744	
Benton	sleep_efficiency	32	-0.21344	0.240826	0.525868	
Benton	num_awakenings	32	0.072994	0.691355	0.849525	
Benton	total_sleep_time_hour	32	0.23031	0.204754	0.481858	
Benton	wake_after_sleep_onset_hour	32	-0.06426	0.726791	0.870173	
Benton	total_nrem_time_hour	32	0.216074	0.234927	0.519805	

Benton	total_rem_time_hour	32	0.199851	0.272792	0.549034	
Benton	total_deep_nrem_time_hour	32	0.082548	0.653322	0.836754	
Benton	total_light_nrem_time_hour	32	0.206816	0.256077	0.536312	
Benton	hourly_mean_pulse_rate	74	-0.07828	0.507383	0.759364	
Benton	hourly_mean_rmssd	73	-0.111	0.349851	0.622996	
Benton	hourly_median_rmssd	73	-0.1116	0.347236	0.622008	
Benton	hourly_rmssd_variance	72	-0.03159	0.792235	0.890844	
Letter Number Sequencing	HVLT Recall	74	0.544284	5.37E-07	2.24E-05	*
Letter Number Sequencing	HVLT Recognition	73	0.120207	0.31106	0.590216	
Letter Number Sequencing	HVLT Retention	74	0.230183	0.048498	0.169998	
Letter Number Sequencing	Symbol Digit	74	0.565994	1.48E-07	8.2E-06	*
Letter Number Sequencing	STAI trait	74	-0.00881	0.940634	0.966763	
Letter Number Sequencing	STAI state	74	-0.05179	0.661221	0.843627	
Letter Number Sequencing	GDS	74	0.021176	0.857872	0.926543	
Letter Number Sequencing	QUIP	74	-0.11803	0.316553	0.594274	
Letter Number Sequencing	ESS	74	0.133973	0.25512	0.535993	
Letter Number Sequencing	RBDSQ	74	-0.10903	0.355117	0.625682	
Letter Number Sequencing	Systolic BP Drop	74	-0.22631	0.052512	0.178434	
Letter Number Sequencing	SCOPA autonome	74	-0.22749	0.051266	0.176908	
Letter Number Sequencing	Schwab England ADL	74	0.142407	0.226142	0.51228	
Letter Number Sequencing	UPDRS I	74	0.047375	0.68855	0.849525	
Letter Number Sequencing	UPDRS II	74	-0.31295	0.006631	0.04843	*
Letter Number Sequencing	UPDRS III OFF	70	-0.27886	0.019406	0.091928	
Letter Number Sequencing	UPDRS III ON	71	-0.34812	0.002931	0.026737	*
Letter Number Sequencing	UPDRS IV	72	-0.10631	0.37413	0.640542	
Letter Number Sequencing	LEDD	74	0.006958	0.953083	0.975043	
Letter Number Sequencing	sum_walking_minutes	74	0.327831	0.004353	0.035358	*
Letter Number Sequencing	hourly_step_count_sum	74	0.463868	3.15E-05	0.000635	*
Letter Number Sequencing	sleep_efficiency	32	0.193689	0.288155	0.564445	
Letter Number Sequencing	num_awakenings	32	-0.13378	0.465416	0.729716	
Letter Number Sequencing	total_sleep_time_hour	32	0.415825	0.017933	0.087821	
Letter Number Sequencing	wake_after_sleep_onset_hour	32	-0.52517	0.002027	0.019281	*
Letter Number Sequencing	total_nrem_time_hour	32	0.458151	0.008366	0.056853	
Letter Number Sequencing	total_rem_time_hour	32	0.198642	0.275763	0.549034	
Letter Number Sequencing	total_deep_nrem_time_hour	32	0.211616	0.244959	0.526267	
Letter Number Sequencing	total_light_nrem_time_hour	32	0.427836	0.01458	0.077681	
Letter Number Sequencing	hourly_mean_pulse_rate	74	0.272332	0.018907	0.09059	
Letter Number Sequencing	hourly_mean_rmssd	73	-0.05791	0.626478	0.820235	
Letter Number Sequencing	hourly_median_rmssd	73	-0.05544	0.641299	0.829331	
Letter Number Sequencing	hourly_rmssd_variance	72	0.029659	0.804667	0.890844	
HVLT Recall	HVLT Recognition	73	0.402225	0.000419	0.005944	*
HVLT Recall	HVLT Retention	74	0.524612	1.6E-06	5.09E-05	*
HVLT Recall	Symbol Digit	74	0.583809	4.77E-08	3.18E-06	*
HVLT Recall	STAI trait	74	-0.18224	0.120165	0.32934	
HVLT Recall	STAI state	74	-0.23122	0.04747	0.167275	
HVLT Recall	GDS	74	-0.12868	0.274538	0.549034	

HVLT Recall	QUIP	74	-0.03295	0.780499	0.890844	
HVLT Recall	ESS	74	0.04698	0.691018	0.849525	
HVLT Recall	RBDSQ	74	0.073164	0.535587	0.774157	
HVLT Recall	Systolic BP Drop	74	-0.26991	0.020036	0.093742	
HVLT Recall	SCOPA autonome	74	0.056578	0.632079	0.825421	
HVLT Recall	Schwab England ADL	74	0.01948	0.869151	0.932133	
HVLT Recall	UPDRS I	74	0.098843	0.402109	0.67119	
HVLT Recall	UPDRS II	74	-0.24185	0.037896	0.145051	
HVLT Recall	UPDRS III OFF	70	-0.36311	0.002005	0.019281	*
HVLT Recall	UPDRS III ON	71	-0.25536	0.031609	0.127632	
HVLT Recall	UPDRS IV	72	-0.06141	0.608325	0.811434	
HVLT Recall	LEDD	74	0.084649	0.473329	0.729716	
HVLT Recall	sum_walking_minutes	74	0.306423	0.007924	0.054409	
HVLT Recall	hourly_step_count_sum	74	0.299174	0.009615	0.061574	
HVLT Recall	sleep_efficiency	32	0.401262	0.022835	0.10276	
HVLT Recall	num_awakenings	32	0.211798	0.244543	0.526267	
HVLT Recall	total_sleep_time_hour	32	0.626706	0.000124	0.002068	*
HVLT Recall	wake_after_sleep_onset_hour	32	-0.19157	0.293566	0.568101	
HVLT Recall	total_nrem_time_hour	32	0.567645	0.000703	0.008669	*
HVLT Recall	total_rem_time_hour	32	0.592273	0.000355	0.005145	*
HVLT Recall	total_deep_nrem_time_hour	32	0.389123	0.027721	0.116114	
HVLT Recall	total_light_nrem_time_hour	32	0.493016	0.004144	0.034424	*
HVLT Recall	hourly_mean_pulse_rate	74	0.111313	0.345076	0.622008	
HVLT Recall	hourly_mean_rmssd	73	-0.27834	0.017107	0.084395	
HVLT Recall	hourly_median_rmssd	73	-0.28489	0.014569	0.077681	
HVLT Recall	hourly_rmssd_variance	72	-0.05413	0.651537	0.836727	
HVLT Recognition	HVLT Retention	73	0.235791	0.044618	0.15976	
HVLT Recognition	Symbol Digit	73	0.153755	0.194031	0.471622	
HVLT Recognition	STAI trait	73	-0.22582	0.054737	0.184116	
HVLT Recognition	STAI state	73	-0.29903	0.010173	0.062734	
HVLT Recognition	GDS	73	-0.2032	0.084673	0.2575	
HVLT Recognition	QUIP	73	0.15935	0.178107	0.446212	
HVLT Recognition	ESS	73	-0.1102	0.353345	0.624239	
HVLT Recognition	RBDSQ	73	0.012504	0.916379	0.958098	
HVLT Recognition	Systolic BP Drop	73	-0.06655	0.575904	0.799067	
HVLT Recognition	SCOPA autonome	73	0.093961	0.429119	0.702195	
HVLT Recognition	Schwab England ADL	73	0.03965	0.739096	0.878996	
HVLT Recognition	UPDRS I	73	-0.03237	0.785741	0.890844	
HVLT Recognition	UPDRS II	73	-0.04444	0.708879	0.859951	
HVLT Recognition	UPDRS III OFF	69	-0.09805	0.422853	0.695358	
HVLT Recognition	UPDRS III ON	70	0.020387	0.866967	0.931707	
HVLT Recognition	UPDRS IV	71	-0.06935	0.565519	0.791251	
HVLT Recognition	LEDD	73	-0.07756	0.514234	0.766174	
HVLT Recognition	sum_walking_minutes	73	-0.10472	0.377936	0.643748	
HVLT Recognition	hourly_step_count_sum	73	-0.00164	0.989027	0.992006	
HVLT Recognition	sleep_efficiency	31	0.074561	0.690165	0.849525	

HVLT Recognition	num_awakenings	31	0.26775	0.145323	0.382411	
HVLT Recognition	total_sleep_time_hour	31	0.403968	0.024209	0.106997	
HVLT Recognition	wake_after_sleep_onset_hour	31	0.042704	0.81957	0.902205	
HVLT Recognition	total_nrem_time_hour	31	0.370383	0.040261	0.149799	
HVLT Recognition	total_rem_time_hour	31	0.374739	0.037793	0.145051	
HVLT Recognition	total_deep_nrem_time_hour	31	0.247415	0.179625	0.4472	
HVLT Recognition	total_light_nrem_time_hour	31	0.318851	0.080414	0.25026	
HVLT Recognition	hourly_mean_pulse_rate	73	-0.01526	0.898055	0.944873	
HVLT Recognition	hourly_mean_rmssd	72	-0.16452	0.167275	0.428481	
HVLT Recognition	hourly_median_rmssd	72	-0.14042	0.2394	0.524475	
HVLT Recognition	hourly_rmssd_variance	71	-0.14781	0.218638	0.504271	
HVLT Retention	Symbol Digit	74	0.32594	0.004598	0.036454	*
HVLT Retention	STAI trait	74	-0.1936	0.098381	0.287375	
HVLT Retention	STAI state	74	-0.19553	0.095018	0.28001	
HVLT Retention	GDS	74	0.038102	0.747224	0.881017	
HVLT Retention	QUIP	74	-0.04427	0.707986	0.859951	
HVLT Retention	ESS	74	0.086353	0.464439	0.729716	
HVLT Retention	RBDSQ	74	-0.14031	0.233129	0.519278	
HVLT Retention	Systolic BP Drop	74	-0.19694	0.092599	0.276315	
HVLT Retention	SCOPA autonome	74	0.150327	0.201099	0.47836	
HVLT Retention	Schwab England ADL	74	0.01975	0.867355	0.931707	
HVLT Retention	UPDRS I	74	-0.01202	0.919066	0.958284	
HVLT Retention	UPDRS II	74	-0.10204	0.386992	0.652498	
HVLT Retention	UPDRS III OFF	70	-0.30766	0.009574	0.061574	
HVLT Retention	UPDRS III ON	71	-0.2073	0.08281	0.25533	
HVLT Retention	UPDRS IV	72	-0.28031	0.017082	0.084395	
HVLT Retention	LEDD	74	0.054327	0.645713	0.831808	
HVLT Retention	sum_walking_minutes	74	0.139949	0.23434	0.519805	
HVLT Retention	hourly_step_count_sum	74	0.177941	0.129323	0.350119	
HVLT Retention	sleep_efficiency	32	0.349082	0.050202	0.175051	
HVLT Retention	num_awakenings	32	0.057638	0.754023	0.881017	
HVLT Retention	total_sleep_time_hour	32	0.445992	0.010515	0.064247	
HVLT Retention	wake_after_sleep_onset_hour	32	-0.28866	0.109096	0.308872	
HVLT Retention	total_nrem_time_hour	32	0.449733	0.009809	0.062217	
HVLT Retention	total_rem_time_hour	32	0.312364	0.081763	0.253275	
HVLT Retention	total_deep_nrem_time_hour	32	0.006616	0.971334	0.98464	
HVLT Retention	total_light_nrem_time_hour	32	0.478709	0.005577	0.042694	*
HVLT Retention	hourly_mean_pulse_rate	74	-0.01038	0.930069	0.961842	
HVLT Retention	hourly_mean_rmssd	73	-0.29731	0.010637	0.064405	
HVLT Retention	hourly_median_rmssd	73	-0.29681	0.010775	0.06465	
HVLT Retention	hourly_rmssd_variance	72	-0.03069	0.798025	0.890844	
Symbol Digit	STAI trait	74	-0.11424	0.332474	0.604994	
Symbol Digit	STAI state	74	-0.21496	0.06588	0.215893	
Symbol Digit	GDS	74	0.035468	0.76417	0.883703	
Symbol Digit	QUIP	74	-0.02999	0.799791	0.890844	
Symbol Digit	ESS	74	0.146276	0.21365	0.499265	

Symbol Digit	RBDSQ	74	-0.14269	0.225218	0.51228	
Symbol Digit	Systolic BP Drop	74	-0.24083	0.038739	0.146593	
Symbol Digit	SCOPA autonome	74	-0.13692	0.244731	0.526267	
Symbol Digit	Schwab England ADL	74	0.129074	0.273069	0.549034	
Symbol Digit	UPDRS I	74	0.050701	0.667927	0.845703	
Symbol Digit	UPDRS II	74	-0.24505	0.03535	0.137678	
Symbol Digit	UPDRS III OFF	70	-0.39047	0.000832	0.0099	*
Symbol Digit	UPDRS III ON	71	-0.36443	0.001782	0.018254	*
Symbol Digit	UPDRS IV	72	-0.04881	0.683911	0.849525	
Symbol Digit	LEDD	74	-0.0095	0.935969	0.963872	
Symbol Digit	sum_walking_minutes	74	0.366247	0.001331	0.014297	*
Symbol Digit	hourly_step_count_sum	74	0.38578	0.000687	0.008639	*
Symbol Digit	sleep_efficiency	32	0.24182	0.182399	0.451591	
Symbol Digit	num_awakenings	32	-0.19462	0.285791	0.561465	
Symbol Digit	total_sleep_time_hour	32	0.422411	0.016023	0.084027	
Symbol Digit	wake_after_sleep_onset_hour	32	-0.42108	0.016394	0.084148	
Symbol Digit	total_nrem_time_hour	32	0.408944	0.020128	0.093742	
Symbol Digit	total_rem_time_hour	32	0.336402	0.059761	0.199005	
Symbol Digit	total_deep_nrem_time_hour	32	0.463539	0.00754	0.052858	
Symbol Digit	total_light_nrem_time_hour	32	0.301675	0.09335	0.276315	
Symbol Digit	hourly_mean_pulse_rate	74	0.163291	0.164493	0.426275	
Symbol Digit	hourly_mean_rmssd	73	-0.03197	0.788297	0.890844	
Symbol Digit	hourly_median_rmssd	73	-0.01759	0.882555	0.93745	
Symbol Digit	hourly_rmssd_variance	72	0.084163	0.482119	0.739842	
STAI trait	STAI state	75	0.7212	2.93E-13	2.79E-11	*
STAI trait	GDS	75	0.642249	5.28E-10	4.39E-08	*
STAI trait	QUIP	75	0.214372	0.064766	0.213535	
STAI trait	ESS	75	0.154242	0.186416	0.458128	
STAI trait	RBDSQ	75	0.109801	0.348362	0.622008	
STAI trait	Systolic BP Drop	75	0.180144	0.121965	0.332906	
STAI trait	SCOPA autonome	75	0.061195	0.60198	0.811434	
STAI trait	Schwab England ADL	75	-0.35199	0.001956	0.019281	*
STAI trait	UPDRS I	75	0.52761	1.15E-06	4.51E-05	*
STAI trait	UPDRS II	75	0.281342	0.014478	0.077681	
STAI trait	UPDRS III OFF	70	0.113034	0.351508	0.624239	
STAI trait	UPDRS III ON	72	0.008635	0.942608	0.967299	
STAI trait	UPDRS IV	73	0.355566	0.002021	0.019281	*
STAI trait	LEDD	75	-0.02992	0.79888	0.890844	
STAI trait	sum_walking_minutes	75	0.020955	0.858374	0.926543	
STAI trait	hourly_step_count_sum	75	-0.12635	0.280046	0.551806	
STAI trait	sleep_efficiency	33	0.120428	0.50441	0.756616	
STAI trait	num_awakenings	33	0.108647	0.547273	0.782154	
STAI trait	total_sleep_time_hour	33	-0.05503	0.761021	0.883703	
STAI trait	wake_after_sleep_onset_hour	33	0.121801	0.499525	0.75243	
STAI trait	total_nrem_time_hour	33	-0.04936	0.785033	0.890844	
STAI trait	total_rem_time_hour	33	-0.05327	0.768429	0.883871	

STAI trait	total_deep_nrem_time_hour	33	-0.05296	0.769737	0.883871	
STAI trait	total_light_nrem_time_hour	33	-0.03724	0.836988	0.913826	
STAI trait	hourly_mean_pulse_rate	75	0.04212	0.719746	0.865254	
STAI trait	hourly_mean_rmssd	74	0.129769	0.270473	0.549034	
STAI trait	hourly_median_rmssd	74	0.10375	0.379037	0.643976	
STAI trait	hourly_rmssd_variance	73	0.130672	0.270495	0.549034	
STAI state	GDS	75	0.568881	1.01E-07	6.11E-06	*
STAI state	QUIP	75	-0.0847	0.469993	0.729716	
STAI state	ESS	75	-0.00468	0.968227	0.98464	
STAI state	RBDSQ	75	0.149553	0.200325	0.47836	
STAI state	Systolic BP Drop	75	0.207331	0.074289	0.23338	
STAI state	SCOPA autonome	75	0.061098	0.602561	0.811434	
STAI state	Schwab England ADL	75	-0.36897	0.001124	0.012451	*
STAI state	UPDRS I	75	0.338008	0.003018	0.027162	*
STAI state	UPDRS II	75	0.236806	0.040801	0.150963	
STAI state	UPDRS III OFF	70	0.257169	0.031621	0.127632	
STAI state	UPDRS III ON	72	0.188855	0.112112	0.311111	
STAI state	UPDRS IV	73	0.305273	0.008633	0.05744	
STAI state	LEDD	75	-0.06752	0.564907	0.791251	
STAI state	sum_walking_minutes	75	-0.10996	0.347673	0.622008	
STAI state	hourly_step_count_sum	75	-0.23367	0.043626	0.157055	
STAI state	sleep_efficiency	33	0.162184	0.367194	0.635198	
STAI state	num_awakenings	33	0.286587	0.105887	0.303969	
STAI state	total_sleep_time_hour	33	-0.04504	0.803451	0.890844	
STAI state	wake_after_sleep_onset_hour	33	0.219905	0.21882	0.504271	
STAI state	total_nrem_time_hour	33	0.009591	0.957754	0.978319	
STAI state	total_rem_time_hour	33	-0.16165	0.368804	0.63633	
STAI state	total_deep_nrem_time_hour	33	-0.08882	0.623038	0.820046	
STAI state	total_light_nrem_time_hour	33	0.03601	0.842302	0.917175	
STAI state	hourly_mean_pulse_rate	75	0.068583	0.558776	0.789928	
STAI state	hourly_mean_rmssd	74	0.159704	0.17409	0.442535	
STAI state	hourly_median_rmssd	74	0.112428	0.340234	0.61575	
STAI state	hourly_rmssd_variance	73	0.083621	0.481832	0.739842	
GDS	QUIP	75	0.114536	0.327839	0.602213	
GDS	ESS	75	0.166251	0.154003	0.40222	
GDS	RBDSQ	75	0.193878	0.095576	0.280413	
GDS	Systolic BP Drop	75	0.156506	0.179954	0.4472	
GDS	SCOPA autonome	75	0.081526	0.486842	0.742027	
GDS	Schwab England ADL	75	-0.32954	0.003889	0.033204	*
GDS	UPDRS I	75	0.457906	3.62E-05	0.000708	*
GDS	UPDRS II	75	0.278436	0.015569	0.082293	
GDS	UPDRS III OFF	70	0.08735	0.472117	0.729716	
GDS	UPDRS III ON	72	-0.06192	0.605357	0.811434	
GDS	UPDRS IV	73	0.316834	0.006313	0.047244	*
GDS	LEDD	75	-0.06234	0.595189	0.810625	
GDS	sum_walking_minutes	75	0.037349	0.750388	0.881017	

GDS	hourly_step_count_sum	75	-0.09068	0.439121	0.710304	
GDS	sleep_efficiency	33	0.119008	0.509487	0.760804	
GDS	num_awakenings	33	0.070056	0.698462	0.853533	
GDS	total_sleep_time_hour	33	-0.04584	0.800032	0.890844	
GDS	wake_after_sleep_onset_hour	33	-0.06553	0.717127	0.863665	
GDS	total_nrem_time_hour	33	-0.05307	0.769294	0.883871	
GDS	total_rem_time_hour	33	-0.01616	0.928878	0.961842	
GDS	total_deep_nrem_time_hour	33	-0.06278	0.72852	0.870173	
GDS	total_light_nrem_time_hour	33	-0.03834	0.832222	0.912922	
GDS	hourly_mean_pulse_rate	75	-0.03484	0.76664	0.883871	
GDS	hourly_mean_rmssd	74	0.165346	0.159173	0.414099	
GDS	hourly_median_rmssd	74	0.135486	0.249747	0.533113	
GDS	hourly_rmssd_variance	73	0.130294	0.271895	0.549034	
QUIP	ESS	75	0.113823	0.330878	0.603739	
QUIP	RBDSQ	75	0.060423	0.606582	0.811434	
QUIP	Systolic BP Drop	75	0.043203	0.712847	0.863193	
QUIP	SCOPA autonome	75	0.110378	0.345818	0.622008	
QUIP	Schwab England ADL	75	-0.02247	0.848252	0.920091	
QUIP	UPDRS I	75	0.249044	0.03119	0.127441	
QUIP	UPDRS II	75	0.148825	0.202549	0.47836	
QUIP	UPDRS III OFF	70	-0.09411	0.438395	0.710304	
QUIP	UPDRS III ON	72	0.068974	0.564812	0.791251	
QUIP	UPDRS IV	73	0.093741	0.430207	0.702249	
QUIP	LEDD	75	0.000728	0.995055	0.995055	
QUIP	sum_walking_minutes	75	0.092798	0.428439	0.702195	
QUIP	hourly_step_count_sum	75	0.03852	0.742826	0.881017	
QUIP	sleep_efficiency	33	-0.01642	0.927715	0.961842	
QUIP	num_awakenings	33	-0.01819	0.919974	0.958284	
QUIP	total_sleep_time_hour	33	0.037749	0.834789	0.912922	
QUIP	wake_after_sleep_onset_hour	33	-0.06621	0.714283	0.863362	
QUIP	total_nrem_time_hour	33	-0.04033	0.823665	0.905217	
QUIP	total_rem_time_hour	33	0.211729	0.236864	0.522355	
QUIP	total_deep_nrem_time_hour	33	0.282812	0.11077	0.30997	
QUIP	total_light_nrem_time_hour	33	-0.12509	0.487912	0.742027	
QUIP	hourly_mean_pulse_rate	75	0.005745	0.960987	0.980119	
QUIP	hourly_mean_rmssd	74	-0.21125	0.070806	0.22563	
QUIP	hourly_median_rmssd	74	-0.16231	0.167081	0.428481	
QUIP	hourly_rmssd_variance	73	0.049888	0.675108	0.84798	
ESS	RBDSQ	75	0.14019	0.230287	0.518146	
ESS	Systolic BP Drop	75	0.114707	0.327111	0.602213	
ESS	SCOPA autonome	75	0.119268	0.308116	0.5867	
ESS	Schwab England ADL	75	-0.03376	0.773694	0.886885	
ESS	UPDRS I	75	0.419289	0.000181	0.002869	*
ESS	UPDRS II	75	0.276275	0.016425	0.084148	
ESS	UPDRS III OFF	70	0.122226	0.313458	0.593077	
ESS	UPDRS III ON	72	0.065698	0.583483	0.803773	

ESS	UPDRS IV	73	0.100094	0.399472	0.670147	
ESS	LEDD	75	0.076028	0.516788	0.76826	
ESS	sum_walking_minutes	75	0.412247	0.000238	0.003683	*
ESS	hourly_step_count_sum	75	0.313678	0.006136	0.046437	*
ESS	sleep_efficiency	33	0.318098	0.071222	0.225876	
ESS	num_awakenings	33	-0.11497	0.524067	0.770548	
ESS	total_sleep_time_hour	33	0.006989	0.969211	0.98464	
ESS	wake_after_sleep_onset_hour	33	-0.13581	0.451109	0.718752	
ESS	total_nrem_time_hour	33	0.006605	0.970899	0.98464	
ESS	total_rem_time_hour	33	0.005971	0.973692	0.98553	
ESS	total_deep_nrem_time_hour	33	0.085071	0.637848	0.827803	
ESS	total_light_nrem_time_hour	33	-0.01766	0.922313	0.958284	
ESS	hourly_mean_pulse_rate	75	0.258814	0.024955	0.107226	
ESS	hourly_mean_rmssd	74	-0.10693	0.364507	0.633471	
ESS	hourly_median_rmssd	74	-0.08529	0.469964	0.729716	
ESS	hourly_rmssd_variance	73	-0.08011	0.50049	0.75243	
RBDSQ	Systolic BP Drop	75	0.258057	0.025397	0.108425	
RBDSQ	SCOPA autonome	75	0.513662	2.44E-06	6.77E-05	*
RBDSQ	Schwab England ADL	75	-0.17558	0.131875	0.354148	
RBDSQ	UPDRS I	75	0.431353	0.000112	0.001907	*
RBDSQ	UPDRS II	75	0.379238	0.000792	0.009596	*
RBDSQ	UPDRS III OFF	70	0.311054	0.008768	0.05744	
RBDSQ	UPDRS III ON	72	0.228491	0.053545	0.18102	
RBDSQ	UPDRS IV	73	0.499159	6.95E-06	0.000154	*
RBDSQ	LEDD	75	0.071846	0.540177	0.777015	
RBDSQ	sum_walking_minutes	75	0.115126	0.325339	0.602213	
RBDSQ	hourly_step_count_sum	75	0.030652	0.794051	0.890844	
RBDSQ	sleep_efficiency	33	0.035574	0.844187	0.917175	
RBDSQ	num_awakenings	33	0.41404	0.016603	0.084395	
RBDSQ	total_sleep_time_hour	33	0.035775	0.843319	0.917175	
RBDSQ	wake_after_sleep_onset_hour	33	0.404768	0.019462	0.091928	
RBDSQ	total_nrem_time_hour	33	-0.03796	0.833878	0.912922	
RBDSQ	total_rem_time_hour	33	0.200041	0.26435	0.546885	
RBDSQ	total_deep_nrem_time_hour	33	-0.20076	0.262604	0.546544	
RBDSQ	total_light_nrem_time_hour	33	0.017816	0.921609	0.958284	
RBDSQ	hourly_mean_pulse_rate	75	0.044341	0.705625	0.859134	
RBDSQ	hourly_mean_rmssd	74	-0.03088	0.793985	0.890844	
RBDSQ	hourly_median_rmssd	74	0.000772	0.99479	0.995055	
RBDSQ	hourly_rmssd_variance	73	-0.13135	0.267993	0.549034	
Systolic BP Drop	SCOPA autonome	75	0.069483	0.553614	0.786156	
Systolic BP Drop	Schwab England ADL	84	-0.14415	0.190794	0.465454	
Systolic BP Drop	UPDRS I	84	0.134763	0.221631	0.507239	
Systolic BP Drop	UPDRS II	84	0.288185	0.007853	0.054409	
Systolic BP Drop	UPDRS III OFF	71	0.328409	0.005172	0.040054	*
Systolic BP Drop	UPDRS III ON	80	0.286012	0.010115	0.062734	
Systolic BP Drop	UPDRS IV	82	0.080418	0.472634	0.729716	

Systolic BP Drop	LEDD	84	0.068556	0.535493	0.774157	
Systolic BP Drop	sum_walking_minutes	84	-0.13486	0.221295	0.507239	
Systolic BP Drop	hourly_step_count_sum	84	-0.18436	0.093185	0.276315	
Systolic BP Drop	sleep_efficiency	36	-0.1794	0.29514	0.568101	
Systolic BP Drop	num_awakenings	36	-0.02383	0.890286	0.941371	
Systolic BP Drop	total_sleep_time_hour	36	-0.26405	0.119673	0.32934	
Systolic BP Drop	wake_after_sleep_onset_hour	36	0.217563	0.202436	0.47836	
Systolic BP Drop	total_nrem_time_hour	36	-0.29173	0.084282	0.257485	
Systolic BP Drop	total_rem_time_hour	36	-0.08994	0.601922	0.811434	
Systolic BP Drop	total_deep_nrem_time_hour	36	-0.10161	0.555395	0.787006	
Systolic BP Drop	total_light_nrem_time_hour	36	-0.27132	0.10945	0.308872	
Systolic BP Drop	hourly_mean_pulse_rate	84	-0.08248	0.455752	0.72189	
Systolic BP Drop	hourly_mean_rmssd	83	-0.01463	0.895561	0.94374	
Systolic BP Drop	hourly_median_rmssd	83	-0.03457	0.756365	0.882205	
Systolic BP Drop	hourly_rmssd_variance	82	0.121452	0.277064	0.549034	
SCOPA autonome	Schwab England ADL	75	-0.30713	0.007357	0.052127	
SCOPA autonome	UPDRS I	75	0.435652	9.36E-05	0.001685	*
SCOPA autonome	UPDRS II	75	0.506357	3.57E-06	8.81E-05	*
SCOPA autonome	UPDRS III OFF	70	0.165156	0.171838	0.438484	
SCOPA autonome	UPDRS III ON	72	0.238631	0.043521	0.157055	
SCOPA autonome	UPDRS IV	73	0.391974	0.000605	0.008013	*
SCOPA autonome	LEDD	75	0.170666	0.143208	0.379985	
SCOPA autonome	sum_walking_minutes	75	0.07261	0.535865	0.774157	
SCOPA autonome	hourly_step_count_sum	75	-0.07574	0.518406	0.768568	
SCOPA autonome	sleep_efficiency	33	-0.04697	0.795219	0.890844	
SCOPA autonome	num_awakenings	33	0.435669	0.011269	0.066416	
SCOPA autonome	total_sleep_time_hour	33	-0.18645	0.298842	0.573571	
SCOPA autonome	wake_after_sleep_onset_hour	33	0.546748	0.000994	0.011413	*
SCOPA autonome	total_nrem_time_hour	33	-0.19954	0.265577	0.547597	
SCOPA autonome	total_rem_time_hour	33	-0.10424	0.563735	0.791251	
SCOPA autonome	total_deep_nrem_time_hour	33	-0.00518	0.977191	0.986679	
SCOPA autonome	total_light_nrem_time_hour	33	-0.21121	0.238047	0.523232	
SCOPA autonome	hourly_mean_pulse_rate	75	-0.0354	0.763038	0.883703	
SCOPA autonome	hourly_mean_rmssd	74	-0.05968	0.613515	0.811434	
SCOPA autonome	hourly_median_rmssd	74	-0.04955	0.675064	0.84798	
SCOPA autonome	hourly_rmssd_variance	73	-0.19389	0.100257	0.291577	
Schwab England ADL	UPDRS I	85	-0.29303	0.006496	0.048072	*
Schwab England ADL	UPDRS II	85	-0.5252	2.46E-07	1.17E-05	*
Schwab England ADL	UPDRS III OFF	71	-0.34474	0.003239	0.028758	*
Schwab England ADL	UPDRS III ON	81	-0.41721	0.000107	0.00187	*
Schwab England ADL	UPDRS IV	83	-0.38342	0.000346	0.005125	*
Schwab England ADL	LEDD	85	-0.09551	0.384567	0.650055	
Schwab England ADL	sum_walking_minutes	85	0.244635	0.024044	0.106997	
Schwab England ADL	hourly_step_count_sum	85	0.3641	0.000614	0.008013	*
Schwab England ADL	sleep_efficiency	36	-0.07065	0.682225	0.849525	
Schwab England ADL	num_awakenings	36	-0.19622	0.251403	0.53323	

Schwab England ADL	total_sleep_time_hour	36	0.356146	0.033013	0.130874	
Schwab England ADL	wake_after_sleep_onset_hour	36	-0.3029	0.072541	0.22897	
Schwab England ADL	total_nrem_time_hour	36	0.355742	0.033227	0.130941	
Schwab England ADL	total_rem_time_hour	36	0.21928	0.198808	0.47836	
Schwab England ADL	total_deep_nrem_time_hour	36	0.021336	0.901704	0.947216	
Schwab England ADL	total_light_nrem_time_hour	36	0.357047	0.032541	0.129774	
Schwab England ADL	hourly_mean_pulse_rate	85	-0.08314	0.449372	0.718752	
Schwab England ADL	hourly_mean_rmssd	84	0.01671	0.88008	0.93745	
Schwab England ADL	hourly_median_rmssd	84	0.088727	0.422209	0.695358	
Schwab England ADL	hourly_rmssd_variance	83	-0.13243	0.232682	0.519278	
UPDRS I	UPDRS II	85	0.482158	2.98E-06	7.93E-05	*
UPDRS I	UPDRS III OFF	71	0.159057	0.185203	0.456835	
UPDRS I	UPDRS III ON	81	0.04712	0.676151	0.84798	
UPDRS I	UPDRS IV	83	0.485663	3.26E-06	8.34E-05	*
UPDRS I	LEDD	85	0.088309	0.421576	0.695358	
UPDRS I	sum_walking_minutes	85	0.107865	0.325796	0.602213	
UPDRS I	hourly_step_count_sum	85	-0.02864	0.794712	0.890844	
UPDRS I	sleep_efficiency	36	0.197525	0.248194	0.531503	
UPDRS I	num_awakenings	36	0.01817	0.91623	0.958098	
UPDRS I	total_sleep_time_hour	36	0.003575	0.983491	0.987941	
UPDRS I	wake_after_sleep_onset_hour	36	0.139645	0.416626	0.690231	
UPDRS I	total_nrem_time_hour	36	-0.05462	0.751703	0.881017	
UPDRS I	total_rem_time_hour	36	0.153289	0.372089	0.640339	
UPDRS I	total_deep_nrem_time_hour	36	0.211768	0.215016	0.500703	
UPDRS I	total_light_nrem_time_hour	36	-0.10972	0.524111	0.770548	
UPDRS I	hourly_mean_pulse_rate	85	-0.05963	0.587744	0.803773	
UPDRS I	hourly_mean_rmssd	84	-0.05673	0.608248	0.811434	
UPDRS I	hourly_median_rmssd	84	-0.0575	0.603379	0.811434	
UPDRS I	hourly_rmssd_variance	83	-0.12258	0.269616	0.549034	
UPDRS II	UPDRS III OFF	71	0.530513	1.95E-06	5.64E-05	*
UPDRS II	UPDRS III ON	81	0.451262	2.36E-05	0.00049	*
UPDRS II	UPDRS IV	83	0.474471	5.86E-06	0.000134	*
UPDRS II	LEDD	85	0.101912	0.35336	0.624239	
UPDRS II	sum_walking_minutes	85	-0.10995	0.316467	0.594274	
UPDRS II	hourly_step_count_sum	85	-0.2547	0.018652	0.090014	
UPDRS II	sleep_efficiency	36	-0.05997	0.728254	0.870173	
UPDRS II	num_awakenings	36	0.143186	0.404788	0.673972	
UPDRS II	total_sleep_time_hour	36	-0.36534	0.028451	0.118426	
UPDRS II	wake_after_sleep_onset_hour	36	0.419054	0.010963	0.065188	
UPDRS II	total_nrem_time_hour	36	-0.42331	0.010098	0.062734	
UPDRS II	total_rem_time_hour	36	-0.07334	0.670796	0.846118	
UPDRS II	total_deep_nrem_time_hour	36	-0.1573	0.35956	0.628522	
UPDRS II	total_light_nrem_time_hour	36	-0.39119	0.018316	0.089039	
UPDRS II	hourly_mean_pulse_rate	85	0.120705	0.271158	0.549034	
UPDRS II	hourly_mean_rmssd	84	-0.12668	0.250873	0.53323	
UPDRS II	hourly_median_rmssd	84	-0.13247	0.229673	0.518146	

UPDRS II	hourly_rmssd_variance	83	-0.11072	0.319007	0.596794	
UPDRS III OFF	UPDRS III ON	67	0.835375	1.5E-18	2E-16	*
UPDRS III OFF	UPDRS IV	69	0.240909	0.046146	0.163888	
UPDRS III OFF	LEDD	71	0.00769	0.949254	0.97262	
UPDRS III OFF	sum_walking_minutes	71	-0.25165	0.034257	0.134205	
UPDRS III OFF	hourly_step_count_sum	71	-0.33401	0.004418	0.035447	*
UPDRS III OFF	sleep_efficiency	31	-0.29004	0.113484	0.313611	
UPDRS III OFF	num_awakenings	31	0.024741	0.894895	0.94374	
UPDRS III OFF	total_sleep_time_hour	31	-0.39134	0.029479	0.121942	
UPDRS III OFF	wake_after_sleep_onset_hour	31	0.215544	0.244208	0.526267	
UPDRS III OFF	total_nrem_time_hour	31	-0.42609	0.016844	0.084395	
UPDRS III OFF	total_rem_time_hour	31	-0.20118	0.277815	0.549034	
UPDRS III OFF	total_deep_nrem_time_hour	31	-0.31343	0.085986	0.259126	
UPDRS III OFF	total_light_nrem_time_hour	31	-0.3678	0.041785	0.15375	
UPDRS III OFF	hourly_mean_pulse_rate	71	0.092183	0.444516	0.715091	
UPDRS III OFF	hourly_mean_rmssd	71	-0.01666	0.890347	0.941371	
UPDRS III OFF	hourly_median_rmssd	71	-0.06146	0.610616	0.811434	
UPDRS III OFF	hourly_rmssd_variance	70	0.091641	0.450542	0.718752	
UPDRS III ON	UPDRS IV	80	0.139887	0.215869	0.500935	
UPDRS III ON	LEDD	81	0.044194	0.695239	0.852724	
UPDRS III ON	sum_walking_minutes	81	-0.27912	0.011622	0.067221	
UPDRS III ON	hourly_step_count_sum	81	-0.35652	0.001087	0.012274	*
UPDRS III ON	sleep_efficiency	34	-0.28168	0.106556	0.304485	
UPDRS III ON	num_awakenings	34	0.144024	0.416429	0.690231	
UPDRS III ON	total_sleep_time_hour	34	-0.16295	0.357147	0.627599	
UPDRS III ON	wake_after_sleep_onset_hour	34	0.21083	0.231357	0.518801	
UPDRS III ON	total_nrem_time_hour	34	-0.1371	0.439407	0.710304	
UPDRS III ON	total_rem_time_hour	34	-0.14895	0.400485	0.670159	
UPDRS III ON	total_deep_nrem_time_hour	34	-0.02023	0.90957	0.953974	
UPDRS III ON	total_light_nrem_time_hour	34	-0.12917	0.466587	0.729716	
UPDRS III ON	hourly_mean_pulse_rate	81	0.002723	0.980753	0.986679	
UPDRS III ON	hourly_mean_rmssd	80	0.051187	0.652044	0.836727	
UPDRS III ON	hourly_median_rmssd	80	0.027997	0.805277	0.890844	
UPDRS III ON	hourly_rmssd_variance	79	-0.05761	0.614058	0.811434	
UPDRS IV	LEDD	83	0.19974	0.070233	0.224879	
UPDRS IV	sum_walking_minutes	83	0.066643	0.549434	0.783562	
UPDRS IV	hourly_step_count_sum	83	-0.04048	0.716331	0.863665	
UPDRS IV	sleep_efficiency	36	0.167188	0.329754	0.603341	
UPDRS IV	num_awakenings	36	0.38023	0.022164	0.100418	
UPDRS IV	total_sleep_time_hour	36	-0.05416	0.75374	0.881017	
UPDRS IV	wake_after_sleep_onset_hour	36	0.493341	0.002233	0.020946	*
UPDRS IV	total_nrem_time_hour	36	-0.10866	0.528171	0.773103	
UPDRS IV	total_rem_time_hour	36	0.108312	0.529486	0.773328	
UPDRS IV	total_deep_nrem_time_hour	36	-0.06691	0.698204	0.853533	
UPDRS IV	total_light_nrem_time_hour	36	-0.09364	0.587004	0.803773	
UPDRS IV	hourly_mean_pulse_rate	83	-0.10989	0.322671	0.601957	

UPDRS IV	hourly_mean_rmssd	82	0.254034	0.02128	0.097741	
UPDRS IV	hourly_median_rmssd	82	0.252581	0.022058	0.100418	
UPDRS IV	hourly_rmssd_variance	81	0.048509	0.667161	0.845703	
LEDD	sum_walking_minutes	85	0.115091	0.294242	0.568101	
LEDD	hourly_step_count_sum	85	0.046833	0.670387	0.846118	
LEDD	sleep_efficiency	36	0.09645	0.575764	0.799067	
LEDD	num_awakenings	36	0.107591	0.532246	0.774157	
LEDD	total_sleep_time_hour	36	0.04679	0.786404	0.890844	
LEDD	wake_after_sleep_onset_hour	36	0.079785	0.643686	0.830803	
LEDD	total_nrem_time_hour	36	0.069222	0.688304	0.849525	
LEDD	total_rem_time_hour	36	-0.02957	0.864068	0.93118	
LEDD	total_deep_nrem_time_hour	36	-0.09363	0.587025	0.803773	
LEDD	total_light_nrem_time_hour	36	0.09444	0.583783	0.803773	
LEDD	hourly_mean_pulse_rate	85	-0.02695	0.806575	0.890844	
LEDD	hourly_mean_rmssd	84	0.068548	0.535542	0.774157	
LEDD	hourly_median_rmssd	84	0.057944	0.600594	0.811434	
LEDD	hourly_rmssd_variance	83	-0.04457	0.689091	0.849525	
sum_walking_minutes	hourly_step_count_sum	85	0.860102	5.52E-26	1.84E-23	*
sum_walking_minutes	sleep_efficiency	36	0.44389	0.00669	0.04843	*
sum_walking_minutes	num_awakenings	36	0.023784	0.890486	0.941371	
sum_walking_minutes	total_sleep_time_hour	36	0.373928	0.024667	0.106997	
sum_walking_minutes	wake_after_sleep_onset_hour	36	-0.14611	0.395159	0.664586	
sum_walking_minutes	total_nrem_time_hour	36	0.415777	0.01167	0.067221	
sum_walking_minutes	total_rem_time_hour	36	0.12047	0.48401	0.741036	
sum_walking_minutes	total_deep_nrem_time_hour	36	0.031581	0.854922	0.925818	
sum_walking_minutes	total_light_nrem_time_hour	36	0.415606	0.011708	0.067221	
sum_walking_minutes	hourly_mean_pulse_rate	85	0.223679	0.039606	0.14819	
sum_walking_minutes	hourly_mean_rmssd	84	0.133569	0.225792	0.51228	
sum_walking_minutes	hourly_median_rmssd	84	0.149065	0.175958	0.443893	
sum_walking_minutes	hourly_rmssd_variance	83	0.035744	0.748357	0.881017	
hourly_step_count_sum	sleep_efficiency	36	0.320028	0.057069	0.190993	
hourly_step_count_sum	num_awakenings	36	-0.12816	0.45633	0.72189	
hourly_step_count_sum	total_sleep_time_hour	36	0.339985	0.042479	0.154755	
hourly_step_count_sum	wake_after_sleep_onset_hour	36	-0.30702	0.068551	0.220556	
hourly_step_count_sum	total_nrem_time_hour	36	0.345606	0.038965	0.146614	
hourly_step_count_sum	total_rem_time_hour	36	0.193733	0.257575	0.537759	
hourly_step_count_sum	total_deep_nrem_time_hour	36	0.047982	0.781095	0.890844	
hourly_step_count_sum	total_light_nrem_time_hour	36	0.339916	0.042523	0.154755	
hourly_step_count_sum	hourly_mean_pulse_rate	85	0.202555	0.063008	0.208773	
hourly_step_count_sum	hourly_mean_rmssd	84	0.126203	0.252657	0.533677	
hourly_step_count_sum	hourly_median_rmssd	84	0.149345	0.175137	0.443502	
hourly_step_count_sum	hourly_rmssd_variance	83	0.056524	0.611767	0.811434	
sleep_efficiency	num_awakenings	36	0.285675	0.091227	0.27368	
sleep_efficiency	total_sleep_time_hour	36	0.742396	2.18E-07	1.12E-05	*
sleep_efficiency	wake_after_sleep_onset_hour	36	-0.02578	0.881369	0.93745	
sleep_efficiency	total_nrem_time_hour	36	0.706311	1.48E-06	4.94E-05	*

sleep_efficiency	total_rem_time_hour	36	0.5486	0.000532	0.007376	*
sleep_efficiency	total_deep_nrem_time_hour	36	0.213475	0.211257	0.495413	
sleep_efficiency	total_light_nrem_time_hour	36	0.665217	9.59E-06	0.000206	*
sleep_efficiency	hourly_mean_pulse_rate	36	0.046089	0.789535	0.890844	
sleep_efficiency	hourly_mean_rmssd	36	0.086353	0.616531	0.813089	
sleep_efficiency	hourly_median_rmssd	36	0.096794	0.574395	0.799067	
sleep_efficiency	hourly_rmssd_variance	35	0.099066	0.571261	0.797609	
num_awakenings	total_sleep_time_hour	36	0.350929	0.035859	0.13885	
num_awakenings	wake_after_sleep_onset_hour	36	0.575084	0.000244	0.003688	*
num_awakenings	total_nrem_time_hour	36	0.395049	0.0171	0.084395	
num_awakenings	total_rem_time_hour	36	0.100482	0.559829	0.789928	
num_awakenings	total_deep_nrem_time_hour	36	-0.14938	0.384548	0.650055	
num_awakenings	total_light_nrem_time_hour	36	0.440683	0.007145	0.051168	
num_awakenings	hourly_mean_pulse_rate	36	-0.07011	0.684516	0.849525	
num_awakenings	hourly_mean_rmssd	36	0.075118	0.663261	0.843729	
num_awakenings	hourly_median_rmssd	36	0.102755	0.550937	0.784026	
num_awakenings	hourly_rmssd_variance	35	-0.18221	0.294812	0.568101	
total_sleep_time_hour	wake_after_sleep_onset_hour	36	-0.15278	0.373696	0.640542	
total_sleep_time_hour	total_nrem_time_hour	36	0.962523	7.59E-21	1.26E-18	*
total_sleep_time_hour	total_rem_time_hour	36	0.710063	1.23E-06	4.56E-05	*
total_sleep_time_hour	total_deep_nrem_time_hour	36	0.275712	0.103607	0.29871	
total_sleep_time_hour	total_light_nrem_time_hour	36	0.910404	1.38E-14	1.53E-12	*
total_sleep_time_hour	hourly_mean_pulse_rate	36	-0.10566	0.539691	0.777015	
total_sleep_time_hour	hourly_mean_rmssd	36	0.151682	0.377184	0.643748	
total_sleep_time_hour	hourly_median_rmssd	36	0.174644	0.308326	0.5867	
total_sleep_time_hour	hourly_rmssd_variance	35	-0.13444	0.441301	0.711638	
wake_after_sleep_onset_hour	total_nrem_time_hour	36	-0.10408	0.545794	0.781718	
wake_after_sleep_onset_hour	total_rem_time_hour	36	-0.22007	0.197157	0.477479	
wake_after_sleep_onset_hour	total_deep_nrem_time_hour	36	-0.3975	0.016364	0.084148	
wake_after_sleep_onset_hour	total_light_nrem_time_hour	36	-0.00457	0.978883	0.986679	
wake_after_sleep_onset_hour	hourly_mean_pulse_rate	36	-0.19103	0.26441	0.546885	
wake_after_sleep_onset_hour	hourly_mean_rmssd	36	0.071787	0.677365	0.84798	
wake_after_sleep_onset_hour	hourly_median_rmssd	36	0.110988	0.519303	0.768568	
wake_after_sleep_onset_hour	hourly_rmssd_variance	35	-0.05616	0.748636	0.881017	
total_nrem_time_hour	total_rem_time_hour	36	0.49249	0.002279	0.021078	*
total_nrem_time_hour	total_deep_nrem_time_hour	36	0.198924	0.244792	0.526267	
total_nrem_time_hour	total_light_nrem_time_hour	36	0.968196	4.87E-22	1.08E-19	*
total_nrem_time_hour	hourly_mean_pulse_rate	36	-0.08094	0.638875	0.827803	
total_nrem_time_hour	hourly_mean_rmssd	36	0.229469	0.178217	0.446212	
total_nrem_time_hour	hourly_median_rmssd	36	0.247339	0.145844	0.382411	
total_nrem_time_hour	hourly_rmssd_variance	35	-0.11821	0.498855	0.75243	
total_rem_time_hour	total_deep_nrem_time_hour	36	0.368318	0.027087	0.114175	
total_rem_time_hour	total_light_nrem_time_hour	36	0.407808	0.013554	0.075223	
total_rem_time_hour	hourly_mean_pulse_rate	36	-0.12891	0.45367	0.721107	
total_rem_time_hour	hourly_mean_rmssd	36	-0.10902	0.52678	0.772766	
total_rem_time_hour	hourly_median_rmssd	36	-0.08173	0.635598	0.826775	

total_rem_time_hour	hourly_rmssd_variance	35	-0.12607	0.470515	0.729716	
total_deep_nrem_time_hour	total_light_nrem_time_hour	36	-0.0526	0.760629	0.883703	
total_deep_nrem_time_hour	hourly_mean_pulse_rate	36	-0.02649	0.878137	0.93745	
total_deep_nrem_time_hour	hourly_mean_rmssd	36	-0.05429	0.753155	0.881017	
total_deep_nrem_time_hour	hourly_median_rmssd	36	-0.02732	0.874344	0.936195	
total_deep_nrem_time_hour	hourly_rmssd_variance	35	-0.05733	0.743589	0.881017	
total_light_nrem_time_hour	hourly_mean_pulse_rate	36	-0.07571	0.660744	0.843627	
total_light_nrem_time_hour	hourly_mean_rmssd	36	0.247684	0.145265	0.382411	
total_light_nrem_time_hour	hourly_median_rmssd	36	0.259007	0.127169	0.345691	
total_light_nrem_time_hour	hourly_rmssd_variance	35	-0.10658	0.542263	0.778335	
hourly_mean_pulse_rate	hourly_mean_rmssd	84	-0.21289	0.051858	0.177419	
hourly_mean_pulse_rate	hourly_median_rmssd	84	-0.1949	0.075634	0.236489	
hourly_mean_pulse_rate	hourly_rmssd_variance	83	0.080936	0.466999	0.729716	
hourly_mean_rmssd	hourly_median_rmssd	84	0.981818	5.98E-61	3.98E-58	*
hourly_mean_rmssd	hourly_rmssd_variance	83	0.246391	0.024741	0.106997	
hourly_median_rmssd	hourly_rmssd_variance	83	0.176002	0.111478	0.310646	

Supplemental Table 3: Performance of digital weekly averages predicting clinical assessments

Results of the predictive modelling of clinical scores from digital weekly averages. For each clinical score the baseline R2 performance and the performance of the model using the digital markers as features are shown as mean R2 and standard deviation. The results of the two-sided T-test comparing the 5-fold cross-validation R2 performances between baseline and fitted model are shown with the t statistic and the associated p-value.

	T	dof	alternative	p-value	CI	cohen-d	BF10	power	sign	mean R2	std R2	baseline mean R2	baseline std R2
updrs_ii	4.566502	8	two-sided	0.001834	[0.05 0.16]	2.888109	17.344	0.977874	*	0.052973	0.022328	-0.05163	0.040002
updrs_iii_OFF	0.505877	8	two-sided	0.626585	[-0.15 0.23]	0.319945	0.533	0.073301		-0.06832	0.113608	-0.10964	0.117366
semantic_fluency	0.40614	8	two-sided	0.695286	[-0.13 0.19]	0.256866	0.519	0.06496		0.118057	0.110473	0.089288	0.08869
moca	0.156266	8	two-sided	0.879695	[-0.08 0.09]	0.098831	0.496	0.0522		-0.0099	0.031966	-0.01557	0.065086
benton	0.117451	8	two-sided	0.909398	[-0.27 0.29]	0.074283	0.495	0.051242		-0.29576	0.171833	-0.30998	0.170644
lns	1.696765	8	two-sided	0.128179	[-0.01 0.09]	1.073128	1.086	0.321514		0.076919	0.042837	0.036734	0.020215
hvlt_recall	1.349826	8	two-sided	0.21402	[-0.05 0.2]	0.853705	0.832	0.222007		-0.07342	0.0777	-0.14752	0.077559
hvlt_recognition	-0.16991	8	two-sided	0.869303	[-0.1 0.08]	0.107457	0.497	0.052602		-0.11071	0.053449	-0.10417	0.055502
hvlt_retention	0.741629	8	two-sided	0.479515	[-0.17 0.33]	0.469048	0.583	0.10064		-0.17287	0.100905	-0.25354	0.192728
symbol_digit	-0.19545	8	two-sided	0.849914	[-0.29 0.24]	0.123613	0.498	0.053444		-0.15414	0.171061	-0.13166	0.153899
stai_trait	-0.04991	8	two-sided	0.961414	[-0.13 0.13]	0.031569	0.493	0.050224		-0.09327	0.082201	-0.09045	0.077069
stai_state	0.175233	8	two-sided	0.865251	[-0.23 0.27]	0.110827	0.497	0.052768		-0.126	0.139052	-0.14482	0.163668

gds	0	8	two-sided	1	[-0.08 0.08]	0	0.492	0.05		-0.11166	0.051324	-0.11166	0.051324
quip	-0.22617	8	two-sided	0.826741	[-0.34 0.28]	0.143042	0.5	0.054615		-0.18318	0.194604	-0.15266	0.187054
epworth	4.537698	8	two-sided	0.001905	[0.05 0.14]	2.869892	16.866	0.976548	*	-0.00021	0.03146	-0.09265	0.025892
rbd	-0.07926	8	two-sided	0.938776	[-0.15 0.14]	0.050125	0.493	0.050565		-0.06457	0.087226	-0.05958	0.090931
systolic_bp_drop	0.021092	8	two-sided	0.983689	[-0.11 0.11]	0.01334	0.492	0.05004		-0.00952	0.071395	-0.01053	0.063237
scopa_aut	0.136293	8	two-sided	0.894957	[-0.19 0.21]	0.086199	0.495	0.051673		-0.15211	0.12849	-0.16401	0.118251
se_adl	1.868912	8	two-sided	0.098572	[-0.02 0.16]	1.182004	1.255	0.376983		-0.09863	0.059547	-0.17237	0.051792
updrs_i	0	8	two-sided	1	[-0.07 0.07]	0	0.492	0.05		-0.06818	0.041239	-0.06818	0.041239
LEDD	-0.3386	8	two-sided	0.743627	[-0.18 0.14]	0.214149	0.51	0.060374		-0.15819	0.105542	-0.13481	0.089034
updrs_iii_ON	1.109388	8	two-sided	0.299495	[-0.08 0.22]	0.701639	0.709	0.165325		0.034923	0.117938	-0.03667	0.052421
updrs_iv	0.318007	8	two-sided	0.758622	[-0.42 0.55]	0.201125	0.508	0.059145		-0.18498	0.293981	-0.25223	0.304119

Supplemental Table 4: Amount of change between clinic visits

For each clinical measure and digital weekly average, the amount of change between visits is quantified with cohen’s d, significance tests between visists are reported with two-sided t-tests.

feature	T	dof	alternative	p-val	CI95%	cohen-d	BF10	power	hedges g	category
semantic_fluency	-1.69002	21	two-sided	0.105816	[-7.81 0.81]	0.207066	0.757	0.152831	0.203346	clinical
moca	-0.81295	21	two-sided	0.425368	[-1.13 0.5]	0.12239	0.3	0.085082	0.120191	clinical
benton	-0.19558	21	two-sided	0.846819	[-0.53 0.44]	0.025273	0.227	0.05147	0.024819	clinical
lns	-1.17611	21	two-sided	0.252712	[-1.38 0.38]	0.190386	0.411	0.136541	0.186965	clinical
hvlt_recall	-3.35808	21	two-sided	0.002977	[-4.12 -0.97]	0.440343	13.911	0.504282	0.432432	clinical

hvlt_recognition	-0.28257	21	two-sided	0.78027	[-0.76 0.58]	0.031854	0.231	0.052336	0.031282	clinical
hvlt_retention	0.608412	21	two-sided	0.549439	[-0.07 0.12]	0.114167	0.264	0.080461	0.112116	clinical
symbol_digit	-1.50966	21	two-sided	0.146031	[-3.03 0.48]	0.101085	0.598	0.073802	0.099269	clinical
stai_trait	-0.63983	22	two-sided	0.528893	[-1.84 0.97]	0.047186	0.263	0.055391	0.046377	clinical
stai_state	1.095844	22	two-sided	0.284999	[-0.78 2.52]	0.07656	0.373	0.064263	0.075247	clinical
gds	1.335223	22	two-sided	0.195456	[-0.24 1.11]	0.13639	0.479	0.095959	0.134052	clinical
quip	0	22	two-sided	1	[-0.26 0.26]	0	0.219	0.05	0	clinical
epworth	-0.17301	22	two-sided	0.864227	[-1.13 0.96]	0.020036	0.222	0.050969	0.019692	clinical
rbd	0.416404	22	two-sided	0.681149	[-0.87 1.3]	0.072792	0.237	0.062884	0.071544	clinical
systolic_bp_drop	-0.9351	34	two-sided	0.356331	[-19.31 7.14]	0.202009	0.272	0.213206	0.199773	clinical
scopa_aut	1.251711	22	two-sided	0.223821	[-0.49 1.96]	0.120661	0.437	0.08581	0.118593	clinical
se_adl	-1.4676	34	two-sided	0.151404	[-4.77 0.77]	0.183168	0.482	0.183563	0.181141	clinical
updrs_i	-1.13044	34	two-sided	0.266203	[-2.16 0.62]	0.137403	0.326	0.12401	0.135882	clinical
updrs_ii	0.804133	34	two-sided	0.426907	[-0.79 1.81]	0.080357	0.245	0.074801	0.079467	clinical
updrs_iii_OFF	-0.87999	21	two-sided	0.388824	[-3.97 1.61]	0.07718	0.315	0.063804	0.075794	clinical
updrs_iii_ON	1.118151	32	two-sided	0.271827	[-1.34 4.62]	0.140179	0.33	0.122329	0.13853	clinical
updrs_iv	1.168987	33	two-sided	0.250784	[-0.48 1.77]	0.203443	0.344	0.210464	0.201122	clinical
LEDD	3.176262	34	two-sided	0.003167	[58.68 267.17]	0.237338	11.513	0.276237	0.234711	clinical
sum_walking_minutes	-1.08132	34	two-sided	0.287163	[-0.91 0.28]	0.153788	0.311	0.143268	0.152086	digital
hourly_step_count_sum	-2.07429	34	two-sided	0.045693	[- 118.08 -1.21]	0.235175	1.212	0.272118	0.232572	digital
sleep_efficiency	1.23733	12	two-sided	0.23963	[-0.06 0.2]	0.314774	0.524	0.181647	0.304834	digital
num_awakenings	1.053825	12	two-sided	0.31272	[-0.73 2.1]	0.359827	0.444	0.222987	0.348464	digital
total_sleep_time_hour	1.048942	12	two-sided	0.314872	[-0.48 1.38]	0.358766	0.442	0.221952	0.347437	digital
wake_after_sleep_onset_hour	0.116099	12	two-sided	0.909494	[-0.28 0.31]	0.04405	0.28	0.052464	0.042659	digital
total_nrem_time_hour	0.677506	12	two-sided	0.510936	[-0.49 0.94]	0.195161	0.339	0.099461	0.188998	digital
total_rem_time_hour	1.785076	12	two-sided	0.099527	[-0.05 0.5]	0.626483	0.963	0.546441	0.606699	digital
total_deep_nrem_time_hour	1.903721	12	two-sided	0.0812	[-0.02 0.27]	0.397333	1.119	0.261402	0.384785	digital
total_light_nrem_time_hour	0.269314	12	two-sided	0.792263	[-0.69 0.88]	0.080628	0.287	0.058281	0.078082	digital

hourly_mean_pulse_rate	-0.59484	34	two-sided	0.555886	[-1.96 1.07]	0.037315	0.214	0.055292	0.036902	digital
hourly_mean_rmssd	1.075409	34	two-sided	0.289764	[-0.67 2.17]	0.19637	0.309	0.20403	0.194196	digital
hourly_median_rmssd	0.743684	34	two-sided	0.462176	[-0.88 1.9]	0.133669	0.234	0.119944	0.132189	digital
hourly_rmssd_variance	1.896054	32	two-sided	0.067014	[-0.57 15.94]	0.319005	0.915	0.427707	0.315252	digital

Supplemental Table 5: Association between rate of change between clinic visits

Results of the Pearson correlation analysis between the rate of change in clinical and in digital markers. The Pearson's r coefficient, the associated p-value, the FDR corrected p-value, and the sample size is displayed.

f1	f2	N	pearson r	p-value
Semantic Fluency	Semantic Fluency			
Semantic Fluency	MOCA	10	-0.02971	0.935064
Semantic Fluency	Benton	10	-0.08715	0.810798
Semantic Fluency	Letter Number Sequencing	10	-0.19901	0.581508
Semantic Fluency	HVLT Recall	10	0.432181	0.212276
Semantic Fluency	HVLT Recognition	10	-0.20997	0.560408
Semantic Fluency	HVLT Retention	10	-0.09308	0.798136
Semantic Fluency	Symbol Digit	10	-0.1787	0.621331
Semantic Fluency	STAI trait	10	0.15847	0.661923
Semantic Fluency	STAI state	10	-0.31079	0.382099
Semantic Fluency	GDS	10	0.228801	0.524886
Semantic Fluency	QUIP	10	-0.15074	0.677657
Semantic Fluency	ESS	10	-0.42505	0.220757
Semantic Fluency	RBDSQ	10	-0.59144	0.071715
Semantic Fluency	Systolic BP Drop	10	-0.27487	0.442129

Semantic Fluency	SCOPA autonome	10	0.05088	0.888988
Semantic Fluency	Schwab England ADL	10	-0.2886	0.418687
Semantic Fluency	UPDRS I	10	0.149542	0.680095
Semantic Fluency	UPDRS II	10	0.370175	0.292377
Semantic Fluency	UPDRS III OFF	8	-0.41767	0.303185
Semantic Fluency	UPDRS III ON	10	-0.16512	0.648492
Semantic Fluency	UPDRS IV	10	0.062942	0.862859
Semantic Fluency	LEDD	9	-0.37688	0.31739
Semantic Fluency	sum_walking_minutes	10	-0.16341	0.651939
Semantic Fluency	hourly_step_count_sum	8	0.010204	0.98087
Semantic Fluency	sleep_efficiency	1		
Semantic Fluency	num_awakenings	1		
Semantic Fluency	total_sleep_time_hour	1		
Semantic Fluency	wake_after_sleep_onset_hour	1		
Semantic Fluency	total_nrem_time_hour	1		
Semantic Fluency	total_rem_time_hour	1		
Semantic Fluency	total_deep_nrem_time_hour	1		
Semantic Fluency	total_light_nrem_time_hour	1		
Semantic Fluency	hourly_mean_pulse_rate	8	-0.4375	0.278347
Semantic Fluency	hourly_mean_rmssd	7	-0.53285	0.218158
Semantic Fluency	hourly_median_rmssd	7	-0.54009	0.210765
Semantic Fluency	hourly_rmssd_variance	5	-0.73223	0.15948
MOCA	Semantic Fluency			
MOCA	MOCA			
MOCA	Benton	10	-0.45444	0.187015
MOCA	Letter Number Sequencing	10	0.350849	0.320219
MOCA	HVLT Recall	10	0.376811	0.283128
MOCA	HVLT Recognition	10	0.668981	0.034404
MOCA	HVLT Retention	10	0.652538	0.040839
MOCA	Symbol Digit	10	0.826161	0.003221

MOCA	STAI trait	10	-0.61691	0.057444
MOCA	STAI state	10	-0.74081	0.014242
MOCA	GDS	10	-0.12996	0.720459
MOCA	QUIP	10	-0.16611	0.646488
MOCA	ESS	10	0.496552	0.144317
MOCA	RBDSQ	10	-0.00131	0.997135
MOCA	Systolic BP Drop	10	-0.39368	0.260345
MOCA	SCOPA autonome	10	0.066395	0.8554
MOCA	Schwab England ADL	10	-0.15877	0.661307
MOCA	UPDRS I	10	0.57154	0.084339
MOCA	UPDRS II	10	0.149608	0.679959
MOCA	UPDRS III OFF	8	-0.3279	0.42784
MOCA	UPDRS III ON	10	-0.3647	0.300132
MOCA	UPDRS IV	10	0.209305	0.561682
MOCA	LEDD	9	0.351903	0.353029
MOCA	sum_walking_minutes	10	-0.26944	0.451563
MOCA	hourly_step_count_sum	8	-0.27421	0.511053
MOCA	sleep_efficiency	1		
MOCA	num_awakenings	1		
MOCA	total_sleep_time_hour	1		
MOCA	wake_after_sleep_onset_hour	1		
MOCA	total_nrem_time_hour	1		
MOCA	total_rem_time_hour	1		
MOCA	total_deep_nrem_time_hour	1		
MOCA	total_light_nrem_time_hour	1		
MOCA	hourly_mean_pulse_rate	8	0.128691	0.761355
MOCA	hourly_mean_rmssd	7	-0.57425	0.177565
MOCA	hourly_median_rmssd	7	-0.55322	0.197676
MOCA	hourly_rmssd_variance	5	-0.23294	0.706116

Benton	Semantic Fluency			
Benton	MOCA			
Benton	Benton			
Benton	Letter Number Sequencing	10	0.380241	0.27841
Benton	HVLT Recall	10	-0.03594	0.921489
Benton	HVLT Recognition	10	0.03062	0.933081
Benton	HVLT Retention	10	-0.04206	0.90815
Benton	Symbol Digit	10	-0.37248	0.289144
Benton	STAI trait	10	0.227659	0.527015
Benton	STAI state	10	0.253942	0.478959
Benton	GDS	10	-0.17439	0.629905
Benton	QUIP	10	0.395037	0.258553
Benton	ESS	10	-0.294	0.409641
Benton	RBDSQ	10	-0.36239	0.303431
Benton	Systolic BP Drop	10	-0.04869	0.893734
Benton	SCOPA autonome	10	-0.68083	0.03022
Benton	Schwab England ADL	10	0.335846	0.342744
Benton	UPDRS I	10	-0.01654	0.963818
Benton	UPDRS II	10	-0.37989	0.278896
Benton	UPDRS III OFF	8	0.154653	0.714616
Benton	UPDRS III ON	10	0.805304	0.004934
Benton	UPDRS IV	10	-0.48291	0.157424
Benton	LEDD	9	-0.08274	0.832404
Benton	sum_walking_minutes	10	0.684868	0.028877
Benton	hourly_step_count_sum	8	0.141267	0.738627
Benton	sleep_efficiency	1		
Benton	num_awakenings	1		
Benton	total_sleep_time_hour	1		
Benton	wake_after_sleep_onset_hour	1		

Benton	total_nrem_time_hour	1		
Benton	total_rem_time_hour	1		
Benton	total_deep_nrem_time_hour	1		
Benton	total_light_nrem_time_hour	1		
Benton	hourly_mean_pulse_rate	8	0.087903	0.836029
Benton	hourly_mean_rmssd	7	0.489797	0.264535
Benton	hourly_median_rmssd	7	0.401177	0.372397
Benton	hourly_rmssd_variance	5	0.007723	0.990166
Letter Number Sequencing	Semantic Fluency			
Letter Number Sequencing	MOCA			
Letter Number Sequencing	Benton			
Letter Number Sequencing	Letter Number Sequencing			
Letter Number Sequencing	HVLT Recall	10	-0.02644	0.942193
Letter Number Sequencing	HVLT Recognition	10	0.254993	0.477079
Letter Number Sequencing	HVLT Retention	10	0.324077	0.360963
Letter Number Sequencing	Symbol Digit	10	0.548582	0.100578
Letter Number Sequencing	STAI trait	10	-0.29894	0.401447
Letter Number Sequencing	STAI state	10	-0.13236	0.715492
Letter Number Sequencing	GDS	10	-0.30497	0.391539
Letter Number Sequencing	QUIP	10	0.105894	0.770936
Letter Number Sequencing	ESS	10	0.19267	0.593834
Letter Number Sequencing	RBDSQ	10	-0.04562	0.900411
Letter Number Sequencing	Systolic BP Drop	10	-0.35941	0.30772
Letter Number Sequencing	SCOPA autonome	10	-0.67134	0.03354
Letter Number Sequencing	Schwab England ADL	10	0.388054	0.267824
Letter Number Sequencing	UPDRS I	10	0.429821	0.215063
Letter Number Sequencing	UPDRS II	10	-0.28528	0.424314
Letter Number Sequencing	UPDRS III OFF	8	-0.53262	0.174131
Letter Number Sequencing	UPDRS III ON	10	0.316058	0.373646
Letter Number Sequencing	UPDRS IV	10	-0.67839	0.031053

Letter Number Sequencing	LEDD	9	-0.06796	0.862086
Letter Number Sequencing	sum_walking_minutes	10	0.329422	0.352629
Letter Number Sequencing	hourly_step_count_sum	8	0.249077	0.551937
Letter Number Sequencing	sleep_efficiency	1		
Letter Number Sequencing	num_awakenings	1		
Letter Number Sequencing	total_sleep_time_hour	1		
Letter Number Sequencing	wake_after_sleep_onset_hour	1		
Letter Number Sequencing	total_nrem_time_hour	1		
Letter Number Sequencing	total_rem_time_hour	1		
Letter Number Sequencing	total_deep_nrem_time_hour	1		
Letter Number Sequencing	total_light_nrem_time_hour	1		
Letter Number Sequencing	hourly_mean_pulse_rate	8	-0.30616	0.46082
Letter Number Sequencing	hourly_mean_rmssd	7	0.206035	0.6576
Letter Number Sequencing	hourly_median_rmssd	7	0.126905	0.78629
Letter Number Sequencing	hourly_rmssd_variance	5	0.330466	0.587026
HVLT Recall	Semantic Fluency			
HVLT Recall	MOCA			
HVLT Recall	Benton			
HVLT Recall	Letter Number Sequencing			
HVLT Recall	HVLT Recall			
HVLT Recall	HVLT Recognition	10	0.217222	0.54662
HVLT Recall	HVLT Retention	10	0.604212	0.064301
HVLT Recall	Symbol Digit	10	0.07969	0.826781
HVLT Recall	STAI trait	10	-0.56203	0.090842
HVLT Recall	STAI state	10	-0.56865	0.086286
HVLT Recall	GDS	10	-0.2324	0.518199
HVLT Recall	QUIP	10	0.040487	0.911581
HVLT Recall	ESS	10	-0.04891	0.893258
HVLT Recall	RBDSQ	10	-0.49078	0.149776
HVLT Recall	Systolic BP Drop	10	-0.7286	0.016846
HVLT Recall	SCOPA autonome	10	0.080802	0.824394

HVLT Recall	Schwab England ADL	10	-0.26114	0.466139
HVLT Recall	UPDRS I	10	0.22775	0.526844
HVLT Recall	UPDRS II	10	0.15655	0.665817
HVLT Recall	UPDRS III OFF	8	-0.1315	0.756272
HVLT Recall	UPDRS III ON	10	0.101201	0.780876
HVLT Recall	UPDRS IV	10	0.215196	0.55046
HVLT Recall	LEDD	9	0.071415	0.85513
HVLT Recall	sum_walking_minutes	10	0.199025	0.581471
HVLT Recall	hourly_step_count_sum	8	-0.16656	0.693421
HVLT Recall	sleep_efficiency	1		
HVLT Recall	num_awakenings	1		
HVLT Recall	total_sleep_time_hour	1		
HVLT Recall	wake_after_sleep_onset_hour	1		
HVLT Recall	total_nrem_time_hour	1		
HVLT Recall	total_rem_time_hour	1		
HVLT Recall	total_deep_nrem_time_hour	1		
HVLT Recall	total_light_nrem_time_hour	1		
HVLT Recall	hourly_mean_pulse_rate	8	0.599491	0.116231
HVLT Recall	hourly_mean_rmssd	7	-0.18346	0.693765
HVLT Recall	hourly_median_rmssd	7	-0.11838	0.800442
HVLT Recall	hourly_rmssd_variance	5	0.128232	0.837179
HVLT Recognition	Semantic Fluency			
HVLT Recognition	MOCA			
HVLT Recognition	Benton			
HVLT Recognition	Letter Number Sequencing			
HVLT Recognition	HVLT Recall			
HVLT Recognition	HVLT Recognition			
HVLT Recognition	HVLT Retention	10	0.681303	0.03006
HVLT Recognition	Symbol Digit	10	0.530301	0.114831
HVLT Recognition	STAI trait	10	-0.52761	0.117034
HVLT Recognition	STAI state	10	-0.69389	0.026025

HVLT Recognition	GDS	10	0.122988	0.734997
HVLT Recognition	QUIP	10	0.197218	0.584978
HVLT Recognition	ESS	10	0.424208	0.221775
HVLT Recognition	RBDSQ	10	0.004607	0.989922
HVLT Recognition	Systolic BP Drop	10	0.032457	0.929075
HVLT Recognition	SCOPA autonome	10	-0.17695	0.624824
HVLT Recognition	Schwab England ADL	10	-0.32682	0.356669
HVLT Recognition	UPDRS I	10	0.697386	0.024973
HVLT Recognition	UPDRS II	10	0.363238	0.302217
HVLT Recognition	UPDRS III OFF	8	0.067286	0.874219
HVLT Recognition	UPDRS III ON	10	0.176662	0.625389
HVLT Recognition	UPDRS IV	10	0.19145	0.596218
HVLT Recognition	LEDD	9	0.381778	0.310629
HVLT Recognition	sum_walking_minutes	10	-0.0755	0.835784
HVLT Recognition	hourly_step_count_sum	8	-0.6454	0.083927
HVLT Recognition	sleep_efficiency	1		
HVLT Recognition	num_awakenings	1		
HVLT Recognition	total_sleep_time_hour	1		
HVLT Recognition	wake_after_sleep_onset_hour	1		
HVLT Recognition	total_nrem_time_hour	1		
HVLT Recognition	total_rem_time_hour	1		
HVLT Recognition	total_deep_nrem_time_hour	1		
HVLT Recognition	total_light_nrem_time_hour	1		
HVLT Recognition	hourly_mean_pulse_rate	8	0.519975	0.186527
HVLT Recognition	hourly_mean_rmssd	7	-0.45637	0.30334
HVLT Recognition	hourly_median_rmssd	7	-0.44541	0.316561
HVLT Recognition	hourly_rmssd_variance	5	-0.30308	0.620095
HVLT Retention	Semantic Fluency			
HVLT Retention	MOCA			
HVLT Retention	Benton			
HVLT Retention	Letter Number Sequencing			

HVLT Retention	HVLT Recall			
HVLT Retention	HVLT Recognition			
HVLT Retention	HVLT Retention			
HVLT Retention	Symbol Digit	10	0.333982	0.345597
HVLT Retention	STAI trait	10	-0.7421	0.013984
HVLT Retention	STAI state	10	-0.81493	0.004078
HVLT Retention	GDS	10	0.051311	0.888052
HVLT Retention	QUIP	10	0.333487	0.346357
HVLT Retention	ESS	10	0.520249	0.123179
HVLT Retention	RBDSQ	10	0.068193	0.85152
HVLT Retention	Systolic BP Drop	10	-0.31388	0.377129
HVLT Retention	SCOPA autonome	10	0.150551	0.678034
HVLT Retention	Schwab England ADL	10	-0.27432	0.443088
HVLT Retention	UPDRS I	10	0.745402	0.01334
HVLT Retention	UPDRS II	10	0.300676	0.398578
HVLT Retention	UPDRS III OFF	8	-0.19911	0.636422
HVLT Retention	UPDRS III ON	10	-0.01578	0.96548
HVLT Retention	UPDRS IV	10	0.209659	0.561005
HVLT Retention	LEDD	9	0.694058	0.038064
HVLT Retention	sum_walking_minutes	10	0.050235	0.890388
HVLT Retention	hourly_step_count_sum	8	-0.63551	0.090377
HVLT Retention	sleep_efficiency	1		
HVLT Retention	num_awakenings	1		
HVLT Retention	total_sleep_time_hour	1		
HVLT Retention	wake_after_sleep_onset_hour	1		
HVLT Retention	total_nrem_time_hour	1		
HVLT Retention	total_rem_time_hour	1		
HVLT Retention	total_deep_nrem_time_hour	1		
HVLT Retention	total_light_nrem_time_hour	1		
HVLT Retention	hourly_mean_pulse_rate	8	0.634788	0.09086

HVLT Retention	hourly_mean_rmssd	7	-0.29234	0.524634
HVLT Retention	hourly_median_rmssd	7	-0.23667	0.609378
HVLT Retention	hourly_rmssd_variance	5	-0.04713	0.940011
Symbol Digit	Semantic Fluency			
Symbol Digit	MOCA			
Symbol Digit	Benton			
Symbol Digit	Letter Number Sequencing			
Symbol Digit	HVLT Recall			
Symbol Digit	HVLT Recognition			
Symbol Digit	HVLT Retention			
Symbol Digit	Symbol Digit			
Symbol Digit	STAI trait	10	-0.6013	0.065946
Symbol Digit	STAI state	10	-0.41972	0.227222
Symbol Digit	GDS	10	-0.35847	0.309086
Symbol Digit	QUIP	10	-0.42983	0.215056
Symbol Digit	ESS	10	0.241383	0.50168
Symbol Digit	RBDSQ	10	0.044649	0.902524
Symbol Digit	Systolic BP Drop	10	-0.29809	0.402842
Symbol Digit	SCOPA autonome	10	-0.28895	0.418103
Symbol Digit	Schwab England ADL	10	0.134386	0.711282
Symbol Digit	UPDRS I	10	0.356174	0.312414
Symbol Digit	UPDRS II	10	0.006729	0.985281
Symbol Digit	UPDRS III OFF	8	-0.32485	0.432395
Symbol Digit	UPDRS III ON	10	-0.14478	0.689844
Symbol Digit	UPDRS IV	10	-0.17891	0.620921
Symbol Digit	LEDD	9	-0.07726	0.843393
Symbol Digit	sum_walking_minutes	10	-0.08616	0.812923
Symbol Digit	hourly_step_count_sum	8	0.187934	0.655833
Symbol Digit	sleep_efficiency	1		
Symbol Digit	num_awakenings	1		

Symbol Digit	total_sleep_time_hour	1		
Symbol Digit	wake_after_sleep_onset_hour	1		
Symbol Digit	total_nrem_time_hour	1		
Symbol Digit	total_rem_time_hour	1		
Symbol Digit	total_deep_nrem_time_hour	1		
Symbol Digit	total_light_nrem_time_hour	1		
Symbol Digit	hourly_mean_pulse_rate	8	-0.22461	0.592809
Symbol Digit	hourly_mean_rmssd	7	-0.40528	0.367059
Symbol Digit	hourly_median_rmssd	7	-0.41816	0.350509
Symbol Digit	hourly_rmssd_variance	5	0.4825	0.410409
STAI trait	Semantic Fluency			
STAI trait	MOCA			
STAI trait	Benton			
STAI trait	Letter Number Sequencing			
STAI trait	HVLT Recall			
STAI trait	HVLT Recognition			
STAI trait	HVLT Retention			
STAI trait	Symbol Digit			
STAI trait	STAI trait			
STAI trait	STAI state	11	0.564923	0.070169
STAI trait	GDS	11	0.371188	0.261054
STAI trait	QUIP	11	0.217534	0.52052
STAI trait	ESS	11	-0.14389	0.672971
STAI trait	RBDSQ	11	-0.16971	0.617863
STAI trait	Systolic BP Drop	11	0.41006	0.210359
STAI trait	SCOPA autonome	11	0.042767	0.900641
STAI trait	Schwab England ADL	11	-0.04037	0.906182
STAI trait	UPDRS I	11	-0.31278	0.349012
STAI trait	UPDRS II	11	-0.25874	0.442348
STAI trait	UPDRS III OFF	9	0.203138	0.600139
STAI trait	UPDRS III ON	10	-0.01455	0.968174
STAI trait	UPDRS IV	10	-0.12595	0.728812

STAI trait	LEDD	10	-0.31066	0.382311
STAI trait	sum_walking_minutes	11	-0.09498	0.781187
STAI trait	hourly_step_count_sum	9	0.085196	0.827487
STAI trait	sleep_efficiency	2	-1	1
STAI trait	num_awakenings	2	1	1
STAI trait	total_sleep_time_hour	2	-1	1
STAI trait	wake_after_sleep_onset_hour	2	1	1
STAI trait	total_nrem_time_hour	2	-1	1
STAI trait	total_rem_time_hour	2	-1	1
STAI trait	total_deep_nrem_time_hour	2	-1	1
STAI trait	total_light_nrem_time_hour	2	-1	1
STAI trait	hourly_mean_pulse_rate	9	-0.38355	0.308196
STAI trait	hourly_mean_rmssd	8	0.345078	0.402509
STAI trait	hourly_median_rmssd	8	0.295191	0.477829
STAI trait	hourly_rmssd_variance	6	-0.45477	0.364874
STAI state	Semantic Fluency			
STAI state	MOCA			
STAI state	Benton			
STAI state	Letter Number Sequencing			
STAI state	HVLT Recall			
STAI state	HVLT Recognition			
STAI state	HVLT Retention			
STAI state	Symbol Digit			
STAI state	STAI trait			
STAI state	STAI state			
STAI state	GDS	11	-0.06509	0.849208
STAI state	QUIP	11	0.006189	0.985592
STAI state	ESS	11	0.182958	0.590259
STAI state	RBDSQ	11	0.166482	0.624671
STAI state	Systolic BP Drop	11	0.304799	0.362096
STAI state	SCOPA autonome	11	-0.30634	0.359546
STAI state	Schwab England ADL	11	0.510976	0.108209
STAI state	UPDRS I	11	-0.77046	0.005522

STAI state	UPDRS II	11	-0.30872	0.35563
STAI state	UPDRS III OFF	9	0.187079	0.629828
STAI state	UPDRS III ON	10	0.279275	0.434545
STAI state	UPDRS IV	10	-0.3949	0.258729
STAI state	LEDD	10	-0.5546	0.096148
STAI state	sum_walking_minutes	11	-0.10212	0.765124
STAI state	hourly_step_count_sum	9	0.669076	0.048746
STAI state	sleep_efficiency	2	-1	1
STAI state	num_awakenings	2	1	1
STAI state	total_sleep_time_hour	2	-1	1
STAI state	wake_after_sleep_onset_hour	2	1	1
STAI state	total_nrem_time_hour	2	-1	1
STAI state	total_rem_time_hour	2	-1	1
STAI state	total_deep_nrem_time_hour	2	-1	1
STAI state	total_light_nrem_time_hour	2	-1	1
STAI state	hourly_mean_pulse_rate	9	-0.33688	0.375355
STAI state	hourly_mean_rmssd	8	0.501023	0.205954
STAI state	hourly_median_rmssd	8	0.475544	0.233661
STAI state	hourly_rmssd_variance	6	0.043132	0.935342
GDS	Semantic Fluency			
GDS	MOCA			
GDS	Benton			
GDS	Letter Number Sequencing			
GDS	HVLT Recall			
GDS	HVLT Recognition			
GDS	HVLT Retention			
GDS	Symbol Digit			
GDS	STAI trait			
GDS	STAI state			
GDS	GDS			
GDS	QUIP	11	0.59703	0.052468
GDS	ESS	11	0.394171	0.230312
GDS	RBDSQ	11	0.270214	0.421617
GDS	Systolic BP Drop	11	0.592221	0.054899

GDS	SCOPA autonome	11	0.314298	0.346551
GDS	Schwab England ADL	11	-0.43067	0.186066
GDS	UPDRS I	11	0.410707	0.209568
GDS	UPDRS II	11	0.685836	0.019813
GDS	UPDRS III OFF	9	-0.18796	0.628183
GDS	UPDRS III ON	10	-0.21595	0.549033
GDS	UPDRS IV	10	0.271601	0.447795
GDS	LEDD	10	0.039222	0.914333
GDS	sum_walking_minutes	11	-0.63514	0.035753
GDS	hourly_step_count_sum	9	-0.58704	0.096538
GDS	sleep_efficiency	2	-1	1
GDS	num_awakenings	2	1	1
GDS	total_sleep_time_hour	2	-1	1
GDS	wake_after_sleep_onset_hour	2	1	1
GDS	total_nrem_time_hour	2	-1	1
GDS	total_rem_time_hour	2	-1	1
GDS	total_deep_nrem_time_hour	2	-1	1
GDS	total_light_nrem_time_hour	2	-1	1
GDS	hourly_mean_pulse_rate	9	0.087561	0.822757
GDS	hourly_mean_rmssd	8	0.203207	0.629345
GDS	hourly_median_rmssd	8	0.23299	0.578696
GDS	hourly_rmssd_variance	6	-0.55704	0.250858
QUIP	Semantic Fluency			
QUIP	MOCA			
QUIP	Benton			
QUIP	Letter Number Sequencing			
QUIP	HVLT Recall			
QUIP	HVLT Recognition			
QUIP	HVLT Retention			
QUIP	Symbol Digit			
QUIP	STAI trait			
QUIP	STAI state			
QUIP	GDS			
QUIP	QUIP			

QUIP	ESS	11	0.344881	0.298946
QUIP	RBDSQ	11	0.15008	0.65961
QUIP	Systolic BP Drop	11	0.126248	0.711469
QUIP	SCOPA autonome	11	0.009541	0.977788
QUIP	Schwab England ADL	11	-0.36635	0.26781
QUIP	UPDRS I	11	0.385525	0.241616
QUIP	UPDRS II	11	0.218917	0.517806
QUIP	UPDRS III OFF	9	-0.18523	0.633275
QUIP	UPDRS III ON	10	0.31335	0.377978
QUIP	UPDRS IV	10	0.049871	0.891178
QUIP	LEDD	10	0.155611	0.667724
QUIP	sum_walking_minutes	11	-0.1683	0.620834
QUIP	hourly_step_count_sum	9	-0.4641	0.208229
QUIP	sleep_efficiency	2	-1	1
QUIP	num_awakenings	2	1	1
QUIP	total_sleep_time_hour	2	-1	1
QUIP	wake_after_sleep_onset_hour	2	1	1
QUIP	total_nrem_time_hour	2	-1	1
QUIP	total_rem_time_hour	2	-1	1
QUIP	total_deep_nrem_time_hour	2	-1	1
QUIP	total_light_nrem_time_hour	2	-1	1
QUIP	hourly_mean_pulse_rate	9	0.303985	0.426454
QUIP	hourly_mean_rmssd	8	0.676869	0.065228
QUIP	hourly_median_rmssd	8	0.694141	0.056127
QUIP	hourly_rmssd_variance	6	-0.74373	0.090097
ESS	Semantic Fluency			
ESS	MOCA			
ESS	Benton			
ESS	Letter Number Sequencing			
ESS	HVLT Recall			
ESS	HVLT Recognition			
ESS	HVLT Retention			
ESS	Symbol Digit			

ESS	STAI trait			
ESS	STAI state			
ESS	GDS			
ESS	QUIP			
ESS	ESS			
ESS	RBDSQ	11	0.467612	0.146967
ESS	Systolic BP Drop	11	0.20875	0.5379
ESS	SCOPA autonome	11	-0.11816	0.729329
ESS	Schwab England ADL	11	0.372375	0.259413
ESS	UPDRS I	11	0.072743	0.831682
ESS	UPDRS II	11	0.421616	0.196515
ESS	UPDRS III OFF	9	-0.37405	0.321335
ESS	UPDRS III ON	10	-0.2644	0.46039
ESS	UPDRS IV	10	0.18952	0.599997
ESS	LEDD	10	-0.02691	0.94118
ESS	sum_walking_minutes	11	-0.7434	0.008738
ESS	hourly_step_count_sum	9	-0.03334	0.932151
ESS	sleep_efficiency	2	-1	1
ESS	num_awakenings	2	1	1
ESS	total_sleep_time_hour	2	-1	1
ESS	wake_after_sleep_onset_hour	2	1	1
ESS	total_nrem_time_hour	2	-1	1
ESS	total_rem_time_hour	2	-1	1
ESS	total_deep_nrem_time_hour	2	-1	1
ESS	total_light_nrem_time_hour	2	-1	1
ESS	hourly_mean_pulse_rate	9	0.203786	0.59895
ESS	hourly_mean_rmssd	8	-0.14157	0.738079
ESS	hourly_median_rmssd	8	-0.10764	0.799723
ESS	hourly_rmssd_variance	6	-0.58889	0.218775
RBDSQ	Semantic Fluency			
RBDSQ	MOCA			
RBDSQ	Benton			
RBDSQ	Letter Number Sequencing			
RBDSQ	HVLT Recall			
RBDSQ	HVLT Recognition			

RBDSQ	HVLT Retention			
RBDSQ	Symbol Digit			
RBDSQ	STAI trait			
RBDSQ	STAI state			
RBDSQ	GDS			
RBDSQ	QUIP			
RBDSQ	ESS			
RBDSQ	RBDSQ			
RBDSQ	Systolic BP Drop	11	0.395405	0.228724
RBDSQ	SCOPA autonome	11	0.270202	0.421638
RBDSQ	Schwab England ADL	11	0.059521	0.861995
RBDSQ	UPDRS I	11	-0.09638	0.778016
RBDSQ	UPDRS II	11	0.342697	0.302219
RBDSQ	UPDRS III OFF	9	-0.20558	0.595658
RBDSQ	UPDRS III ON	10	-0.39044	0.264639
RBDSQ	UPDRS IV	10	0.349427	0.322319
RBDSQ	LEDD	10	0.20897	0.562323
RBDSQ	sum_walking_minutes	11	-0.5019	0.115695
RBDSQ	hourly_step_count_sum	9	-0.08971	0.818473
RBDSQ	sleep_efficiency	2	-1	1
RBDSQ	num_awakenings	2	1	1
RBDSQ	total_sleep_time_hour	2	-1	1
RBDSQ	wake_after_sleep_onset_hour	2	1	1
RBDSQ	total_nrem_time_hour	2	-1	1
RBDSQ	total_rem_time_hour	2	-1	1
RBDSQ	total_deep_nrem_time_hour	2	-1	1
RBDSQ	total_light_nrem_time_hour	2	-1	1
RBDSQ	hourly_mean_pulse_rate	9	-0.12385	0.750907
RBDSQ	hourly_mean_rmssd	8	-0.02977	0.944219
RBDSQ	hourly_median_rmssd	8	0.031006	0.941901
RBDSQ	hourly_rmssd_variance	6	-0.19218	0.715282
Systolic BP Drop	Semantic Fluency			
Systolic BP Drop	MOCA			

Systolic BP Drop	Benton			
Systolic BP Drop	Letter Number Sequencing			
Systolic BP Drop	HVLT Recall			
Systolic BP Drop	HVLT Recognition			
Systolic BP Drop	HVLT Retention			
Systolic BP Drop	Symbol Digit			
Systolic BP Drop	STAI trait			
Systolic BP Drop	STAI state			
Systolic BP Drop	GDS			
Systolic BP Drop	QUIP			
Systolic BP Drop	ESS			
Systolic BP Drop	RBDSQ			
Systolic BP Drop	Systolic BP Drop			
Systolic BP Drop	SCOPA autonome	11	0.167718	0.622064
Systolic BP Drop	Schwab England ADL	35	0.046823	0.789398
Systolic BP Drop	UPDRS I	35	-0.0745	0.670603
Systolic BP Drop	UPDRS II	35	0.246284	0.153812
Systolic BP Drop	UPDRS III OFF	10	0.35728	0.310806
Systolic BP Drop	UPDRS III ON	33	-0.12783	0.478383
Systolic BP Drop	UPDRS IV	34	-0.37764	0.027671
Systolic BP Drop	LEDD	34	0.005534	0.975222
Systolic BP Drop	sum_walking_minutes	35	-0.08293	0.635753
Systolic BP Drop	hourly_step_count_sum	33	-0.0253	0.888852
Systolic BP Drop	sleep_efficiency	11	-0.42716	0.190073
Systolic BP Drop	num_awakenings	11	-0.58903	0.056554
Systolic BP Drop	total_sleep_time_hour	11	-0.32102	0.335767
Systolic BP Drop	wake_after_sleep_onset_hour	11	-0.38561	0.24151
Systolic BP Drop	total_nrem_time_hour	11	-0.28424	0.396939
Systolic BP Drop	total_rem_time_hour	11	-0.3517	0.288851
Systolic BP Drop	total_deep_nrem_time_hour	11	-0.36799	0.265503
Systolic BP Drop	total_light_nrem_time_hour	11	-0.15602	0.646876

Systolic BP Drop	hourly_mean_pulse_rate	33	-0.15218	0.397852
Systolic BP Drop	hourly_mean_rmssd	32	0.094591	0.606584
Systolic BP Drop	hourly_median_rmssd	32	0.08412	0.647143
Systolic BP Drop	hourly_rmssd_variance	29	0.198524	0.301894
SCOPA autonome	Semantic Fluency			
SCOPA autonome	MOCA			
SCOPA autonome	Benton			
SCOPA autonome	Letter Number Sequencing			
SCOPA autonome	HVLT Recall			
SCOPA autonome	HVLT Recognition			
SCOPA autonome	HVLT Retention			
SCOPA autonome	Symbol Digit			
SCOPA autonome	STAI trait			
SCOPA autonome	STAI state			
SCOPA autonome	GDS			
SCOPA autonome	QUIP			
SCOPA autonome	ESS			
SCOPA autonome	RBDSQ			
SCOPA autonome	Systolic BP Drop			
SCOPA autonome	SCOPA autonome			
SCOPA autonome	Schwab England ADL	11	-0.4893	0.12664
SCOPA autonome	UPDRS I	11	0.069698	0.838644
SCOPA autonome	UPDRS II	11	0.145885	0.668648
SCOPA autonome	UPDRS III OFF	9	0.21425	0.579887
SCOPA autonome	UPDRS III ON	10	-0.76543	0.00987
SCOPA autonome	UPDRS IV	10	0.720648	0.01871
SCOPA autonome	LEDD	10	0.651349	0.041333
SCOPA autonome	sum_walking_minutes	11	-0.17452	0.607794
SCOPA autonome	hourly_step_count_sum	9	-0.64537	0.060494
SCOPA autonome	sleep_efficiency	2	1	1
SCOPA autonome	num_awakenings	2	-1	1
SCOPA autonome	total_sleep_time_hour	2	1	1
SCOPA autonome	wake_after_sleep_onset_hour	2	-1	1
SCOPA autonome	total_nrem_time_hour	2	1	1
SCOPA autonome	total_rem_time_hour	2	1	1
SCOPA autonome	total_deep_nrem_time_hour	2	1	1
SCOPA autonome	total_light_nrem_time_hour	2	1	1

SCOPA autonome	hourly_mean_pulse_rate	9	0.317679	0.404815
SCOPA autonome	hourly_mean_rmssd	8	-0.26399	0.527527
SCOPA autonome	hourly_median_rmssd	8	-0.18314	0.664212
SCOPA autonome	hourly_rmssd_variance	6	-0.22403	0.669574
Schwab England ADL	Semantic Fluency			
Schwab England ADL	MOCA			
Schwab England ADL	Benton			
Schwab England ADL	Letter Number Sequencing			
Schwab England ADL	HVLT Recall			
Schwab England ADL	HVLT Recognition			
Schwab England ADL	HVLT Retention			
Schwab England ADL	Symbol Digit			
Schwab England ADL	STAI trait			
Schwab England ADL	STAI state			
Schwab England ADL	GDS			
Schwab England ADL	QUIP			
Schwab England ADL	ESS			
Schwab England ADL	RBDSQ			
Schwab England ADL	Systolic BP Drop			
Schwab England ADL	SCOPA autonome			
Schwab England ADL	Schwab England ADL			
Schwab England ADL	UPDRS I	35	-0.18051	0.299414
Schwab England ADL	UPDRS II	35	-0.20842	0.229558
Schwab England ADL	UPDRS III OFF	10	-0.01455	0.968178
Schwab England ADL	UPDRS III ON	33	-0.31251	0.076614
Schwab England ADL	UPDRS IV	34	-0.24435	0.163709
Schwab England ADL	LEDD	34	-0.453	0.007138
Schwab England ADL	sum_walking_minutes	35	0.088261	0.614132
Schwab England ADL	hourly_step_count_sum	33	0.198384	0.26841
Schwab England ADL	sleep_efficiency	11	-0.0842	0.805587
Schwab England ADL	num_awakenings	11	0.287981	0.390476
Schwab England ADL	total_sleep_time_hour	11	0.260673	0.438818
Schwab England ADL	wake_after_sleep_onset_hour	11	0.214653	0.526195

Schwab England ADL	total_nrem_time_hour	11	0.22933	0.497563
Schwab England ADL	total_rem_time_hour	11	0.288666	0.389299
Schwab England ADL	total_deep_nrem_time_hour	11	-0.28107	0.402444
Schwab England ADL	total_light_nrem_time_hour	11	0.36085	0.275602
Schwab England ADL	hourly_mean_pulse_rate	33	-0.28311	0.110374
Schwab England ADL	hourly_mean_rmssd	32	-0.32294	0.071422
Schwab England ADL	hourly_median_rmssd	32	-0.32863	0.066291
Schwab England ADL	hourly_rmssd_variance	29	-0.16448	0.393883
UPDRS I	Semantic Fluency			
UPDRS I	MOCA			
UPDRS I	Benton			
UPDRS I	Letter Number Sequencing			
UPDRS I	HVLT Recall			
UPDRS I	HVLT Recognition			
UPDRS I	HVLT Retention			
UPDRS I	Symbol Digit			
UPDRS I	STAI trait			
UPDRS I	STAI state			
UPDRS I	GDS			
UPDRS I	QUIP			
UPDRS I	ESS			
UPDRS I	RBDSQ			
UPDRS I	Systolic BP Drop			
UPDRS I	SCOPA autonome			
UPDRS I	Schwab England ADL			
UPDRS I	UPDRS I			
UPDRS I	UPDRS II	35	0.462103	0.005195
UPDRS I	UPDRS III OFF	10	-0.29464	0.40857
UPDRS I	UPDRS III ON	33	-0.2095	0.24195
UPDRS I	UPDRS IV	34	0.326324	0.059628
UPDRS I	LEDD	34	-0.00194	0.991307
UPDRS I	sum_walking_minutes	35	-0.09697	0.579474
UPDRS I	hourly_step_count_sum	33	-0.28214	0.11166
UPDRS I	sleep_efficiency	11	0.566731	0.069078
UPDRS I	num_awakenings	11	-0.03549	0.917499

UPDRS I	total_sleep_time_hour	11	0.433613	0.182738
UPDRS I	wake_after_sleep_onset_hour	11	-0.1159	0.734346
UPDRS I	total_nrem_time_hour	11	0.376643	0.253557
UPDRS I	total_rem_time_hour	11	0.490269	0.125773
UPDRS I	total_deep_nrem_time_hour	11	0.832718	0.001464
UPDRS I	total_light_nrem_time_hour	11	0.066455	0.846073
UPDRS I	hourly_mean_pulse_rate	33	-0.20764	0.246247
UPDRS I	hourly_mean_rmssd	32	0.07785	0.671926
UPDRS I	hourly_median_rmssd	32	0.123407	0.501012
UPDRS I	hourly_rmssd_variance	29	0.086327	0.656124
UPDRS II	Semantic Fluency			
UPDRS II	MOCA			
UPDRS II	Benton			
UPDRS II	Letter Number Sequencing			
UPDRS II	HVLT Recall			
UPDRS II	HVLT Recognition			
UPDRS II	HVLT Retention			
UPDRS II	Symbol Digit			
UPDRS II	STAI trait			
UPDRS II	STAI state			
UPDRS II	GDS			
UPDRS II	QUIP			
UPDRS II	ESS			
UPDRS II	RBDSQ			
UPDRS II	Systolic BP Drop			
UPDRS II	SCOPA autonome			
UPDRS II	Schwab England ADL			
UPDRS II	UPDRS I			
UPDRS II	UPDRS II			
UPDRS II	UPDRS III OFF	10	-0.39315	0.261035
UPDRS II	UPDRS III ON	33	-0.20743	0.24673
UPDRS II	UPDRS IV	34	0.12563	0.478975
UPDRS II	LEDD	34	-0.10223	0.565076
UPDRS II	sum_walking_minutes	35	-0.08615	0.622672

UPDRS II	hourly_step_count_sum	33	-0.05371	0.766557
UPDRS II	sleep_efficiency	11	0.155637	0.6477
UPDRS II	num_awakenings	11	-0.22686	0.502334
UPDRS II	total_sleep_time_hour	11	0.029075	0.932373
UPDRS II	wake_after_sleep_onset_hour	11	-0.07212	0.833102
UPDRS II	total_nrem_time_hour	11	0.107321	0.753462
UPDRS II	total_rem_time_hour	11	-0.13844	0.684793
UPDRS II	total_deep_nrem_time_hour	11	-0.07553	0.825313
UPDRS II	total_light_nrem_time_hour	11	0.146102	0.66818
UPDRS II	hourly_mean_pulse_rate	33	-0.06088	0.736438
UPDRS II	hourly_mean_rmssd	32	0.066452	0.717835
UPDRS II	hourly_median_rmssd	32	0.127348	0.487328
UPDRS II	hourly_rmssd_variance	29	0.336378	0.074396
UPDRS III OFF	Semantic Fluency			
UPDRS III OFF	MOCA			
UPDRS III OFF	Benton			
UPDRS III OFF	Letter Number Sequencing			
UPDRS III OFF	HVLT Recall			
UPDRS III OFF	HVLT Recognition			
UPDRS III OFF	HVLT Retention			
UPDRS III OFF	Symbol Digit			
UPDRS III OFF	STAI trait			
UPDRS III OFF	STAI state			
UPDRS III OFF	GDS			
UPDRS III OFF	QUIP			
UPDRS III OFF	ESS			
UPDRS III OFF	RBDSQ			
UPDRS III OFF	Systolic BP Drop			
UPDRS III OFF	SCOPA autonome			
UPDRS III OFF	Schwab England ADL			
UPDRS III OFF	UPDRS I			
UPDRS III OFF	UPDRS II			
UPDRS III OFF	UPDRS III OFF			
UPDRS III OFF	UPDRS III ON	8	0.325189	0.431892

UPDRS III OFF	UPDRS IV	9	0.140625	0.718201
UPDRS III OFF	LEDD	9	0.300664	0.431777
UPDRS III OFF	sum_walking_minutes	10	0.401941	0.249563
UPDRS III OFF	hourly_step_count_sum	8	-0.21402	0.610791
UPDRS III OFF	sleep_efficiency	1		
UPDRS III OFF	num_awakenings	1		
UPDRS III OFF	total_sleep_time_hour	1		
UPDRS III OFF	wake_after_sleep_onset_hour	1		
UPDRS III OFF	total_nrem_time_hour	1		
UPDRS III OFF	total_rem_time_hour	1		
UPDRS III OFF	total_deep_nrem_time_hour	1		
UPDRS III OFF	total_light_nrem_time_hour	1		
UPDRS III OFF	hourly_mean_pulse_rate	8	0.580153	0.13165
UPDRS III OFF	hourly_mean_rmssd	7	0.16138	0.729586
UPDRS III OFF	hourly_median_rmssd	7	0.205856	0.657886
UPDRS III OFF	hourly_rmssd_variance	6	0.475003	0.341082
UPDRS III ON	Semantic Fluency			
UPDRS III ON	MOCA			
UPDRS III ON	Benton			
UPDRS III ON	Letter Number Sequencing			
UPDRS III ON	HVLT Recall			
UPDRS III ON	HVLT Recognition			
UPDRS III ON	HVLT Retention			
UPDRS III ON	Symbol Digit			
UPDRS III ON	STAI trait			
UPDRS III ON	STAI state			
UPDRS III ON	GDS			
UPDRS III ON	QUIP			
UPDRS III ON	ESS			
UPDRS III ON	RBDSQ			
UPDRS III ON	Systolic BP Drop			
UPDRS III ON	SCOPA autonome			
UPDRS III ON	Schwab England ADL			
UPDRS III ON	UPDRS I			
UPDRS III ON	UPDRS II			
UPDRS III ON	UPDRS III OFF			
UPDRS III ON	UPDRS III ON			
UPDRS III ON	UPDRS IV	33	-0.09323	0.605813

UPDRS III ON	LEDD	32	0.510962	0.002804
UPDRS III ON	sum_walking_minutes	33	-0.01903	0.916268
UPDRS III ON	hourly_step_count_sum	31	-0.15672	0.399825
UPDRS III ON	sleep_efficiency	10	0.348547	0.323622
UPDRS III ON	num_awakenings	10	0.212759	0.555091
UPDRS III ON	total_sleep_time_hour	10	0.549217	0.100104
UPDRS III ON	wake_after_sleep_onset_hour	10	0.068813	0.850182
UPDRS III ON	total_nrem_time_hour	10	0.398581	0.253915
UPDRS III ON	total_rem_time_hour	10	0.783418	0.007343
UPDRS III ON	total_deep_nrem_time_hour	10	0.281607	0.430556
UPDRS III ON	total_light_nrem_time_hour	10	0.313553	0.377653
UPDRS III ON	hourly_mean_pulse_rate	31	0.366974	0.042282
UPDRS III ON	hourly_mean_rmssd	30	0.193313	0.30606
UPDRS III ON	hourly_median_rmssd	30	0.193647	0.305209
UPDRS III ON	hourly_rmssd_variance	27	-0.2898	0.142574
UPDRS IV	Semantic Fluency			
UPDRS IV	MOCA			
UPDRS IV	Benton			
UPDRS IV	Letter Number Sequencing			
UPDRS IV	HVLT Recall			
UPDRS IV	HVLT Recognition			
UPDRS IV	HVLT Retention			
UPDRS IV	Symbol Digit			
UPDRS IV	STAI trait			
UPDRS IV	STAI state			
UPDRS IV	GDS			
UPDRS IV	QUIP			
UPDRS IV	ESS			
UPDRS IV	RBDSQ			
UPDRS IV	Systolic BP Drop			
UPDRS IV	SCOPA autonome			
UPDRS IV	Schwab England ADL			
UPDRS IV	UPDRS I			
UPDRS IV	UPDRS II			

UPDRS IV	UPDRS III OFF			
UPDRS IV	UPDRS III ON			
UPDRS IV	UPDRS IV			
UPDRS IV	LEDD	33	0.168254	0.349286
UPDRS IV	sum_walking_minutes	34	0.062761	0.724376
UPDRS IV	hourly_step_count_sum	32	-0.09915	0.589258
UPDRS IV	sleep_efficiency	10	0.32001	0.367368
UPDRS IV	num_awakenings	10	0.436174	0.207608
UPDRS IV	total_sleep_time_hour	10	0.012989	0.971592
UPDRS IV	wake_after_sleep_onset_hour	10	0.319307	0.36848
UPDRS IV	total_nrem_time_hour	10	0.035974	0.921409
UPDRS IV	total_rem_time_hour	10	-0.03866	0.915566
UPDRS IV	total_deep_nrem_time_hour	10	0.42371	0.222377
UPDRS IV	total_light_nrem_time_hour	10	-0.10657	0.769504
UPDRS IV	hourly_mean_pulse_rate	32	0.219704	0.226964
UPDRS IV	hourly_mean_rmssd	31	-0.06027	0.747407
UPDRS IV	hourly_median_rmssd	31	0.013361	0.943131
UPDRS IV	hourly_rmssd_variance	28	-0.21602	0.269576
LEDD	Semantic Fluency			
LEDD	MOCA			
LEDD	Benton			
LEDD	Letter Number Sequencing			
LEDD	HVLT Recall			
LEDD	HVLT Recognition			
LEDD	HVLT Retention			
LEDD	Symbol Digit			
LEDD	STAI trait			
LEDD	STAI state			
LEDD	GDS			
LEDD	QUIP			
LEDD	ESS			
LEDD	RBDSQ			
LEDD	Systolic BP Drop			
LEDD	SCOPA autonome			

LEDD	Schwab England ADL			
LEDD	UPDRS I			
LEDD	UPDRS II			
LEDD	UPDRS III OFF			
LEDD	UPDRS III ON			
LEDD	UPDRS IV			
LEDD	LEDD			
LEDD	sum_walking_minutes	34	0.079694	0.654137
LEDD	hourly_step_count_sum	32	-0.11283	0.538654
LEDD	sleep_efficiency	11	0.450925	0.163922
LEDD	num_awakenings	11	-0.54007	0.086345
LEDD	total_sleep_time_hour	11	0.139427	0.682641
LEDD	wake_after_sleep_onset_hour	11	-0.49912	0.11806
LEDD	total_nrem_time_hour	11	0.15645	0.645963
LEDD	total_rem_time_hour	11	0.083868	0.806331
LEDD	total_deep_nrem_time_hour	11	0.67931	0.021505
LEDD	total_light_nrem_time_hour	11	-0.10794	0.752081
LEDD	hourly_mean_pulse_rate	32	0.238988	0.187733
LEDD	hourly_mean_rmssd	31	0.117643	0.528506
LEDD	hourly_median_rmssd	31	0.141192	0.448674
LEDD	hourly_rmssd_variance	29	-0.23501	0.219761
sum_walking_minutes	Semantic Fluency			
sum_walking_minutes	MOCA			
sum_walking_minutes	Benton			
sum_walking_minutes	Letter Number Sequencing			
sum_walking_minutes	HVLT Recall			
sum_walking_minutes	HVLT Recognition			
sum_walking_minutes	HVLT Retention			
sum_walking_minutes	Symbol Digit			
sum_walking_minutes	STAI trait			
sum_walking_minutes	STAI state			
sum_walking_minutes	GDS			
sum_walking_minutes	QUIP			
sum_walking_minutes	ESS			
sum_walking_minutes	RBDSQ			

sum_walking_minutes	Systolic BP Drop			
sum_walking_minutes	SCOPA autonome			
sum_walking_minutes	Schwab England ADL			
sum_walking_minutes	UPDRS I			
sum_walking_minutes	UPDRS II			
sum_walking_minutes	UPDRS III OFF			
sum_walking_minutes	UPDRS III ON			
sum_walking_minutes	UPDRS IV			
sum_walking_minutes	LEDD			
sum_walking_minutes	sum_walking_minutes			
sum_walking_minutes	hourly_step_count_sum	33	0.842929	7.53E-10
sum_walking_minutes	sleep_efficiency	11	0.157419	0.643897
sum_walking_minutes	num_awakenings	11	-0.61583	0.04367
sum_walking_minutes	total_sleep_time_hour	11	-0.10501	0.758637
sum_walking_minutes	wake_after_sleep_onset_hour	11	-0.65963	0.027226
sum_walking_minutes	total_nrem_time_hour	11	-0.11388	0.738839
sum_walking_minutes	total_rem_time_hour	11	-0.07141	0.834721
sum_walking_minutes	total_deep_nrem_time_hour	11	0.489143	0.126777
sum_walking_minutes	total_light_nrem_time_hour	11	-0.3213	0.335322
sum_walking_minutes	hourly_mean_pulse_rate	33	0.404906	0.019417
sum_walking_minutes	hourly_mean_rmssd	32	-0.26671	0.14005
sum_walking_minutes	hourly_median_rmssd	32	-0.25626	0.156859
sum_walking_minutes	hourly_rmssd_variance	29	-0.09995	0.605939
hourly_step_count_sum	Semantic Fluency			
hourly_step_count_sum	MOCA			
hourly_step_count_sum	Benton			
hourly_step_count_sum	Letter Number Sequencing			
hourly_step_count_sum	HVLT Recall			
hourly_step_count_sum	HVLT Recognition			
hourly_step_count_sum	HVLT Retention			
hourly_step_count_sum	Symbol Digit			
hourly_step_count_sum	STAI trait			
hourly_step_count_sum	STAI state			
hourly_step_count_sum	GDS			
hourly_step_count_sum	QUIP			
hourly_step_count_sum	ESS			
hourly_step_count_sum	RBDSQ			

hourly_step_count_sum	Systolic BP Drop			
hourly_step_count_sum	SCOPA autonome			
hourly_step_count_sum	Schwab England ADL			
hourly_step_count_sum	UPDRS I			
hourly_step_count_sum	UPDRS II			
hourly_step_count_sum	UPDRS III OFF			
hourly_step_count_sum	UPDRS III ON			
hourly_step_count_sum	UPDRS IV			
hourly_step_count_sum	LEDD			
hourly_step_count_sum	sum_walking_minutes			
hourly_step_count_sum	hourly_step_count_sum			
hourly_step_count_sum	sleep_efficiency	11	-0.24147	0.474405
hourly_step_count_sum	num_awakenings	11	-0.69269	0.018141
hourly_step_count_sum	total_sleep_time_hour	11	-0.39876	0.224444
hourly_step_count_sum	wake_after_sleep_onset_hour	11	-0.62416	0.040117
hourly_step_count_sum	total_nrem_time_hour	11	-0.36696	0.266946
hourly_step_count_sum	total_rem_time_hour	11	-0.40786	0.213055
hourly_step_count_sum	total_deep_nrem_time_hour	11	0.020737	0.951743
hourly_step_count_sum	total_light_nrem_time_hour	11	-0.403	0.219089
hourly_step_count_sum	hourly_mean_pulse_rate	33	0.237782	0.182699
hourly_step_count_sum	hourly_mean_rmssd	32	-0.26921	0.136236
hourly_step_count_sum	hourly_median_rmssd	32	-0.26806	0.137987
hourly_step_count_sum	hourly_rmssd_variance	29	0.061611	0.750871
sleep_efficiency	Semantic Fluency			
sleep_efficiency	MOCA			
sleep_efficiency	Benton			
sleep_efficiency	Letter Number Sequencing			
sleep_efficiency	HVLT Recall			
sleep_efficiency	HVLT Recognition			
sleep_efficiency	HVLT Retention			
sleep_efficiency	Symbol Digit			
sleep_efficiency	STAI trait			
sleep_efficiency	STAI state			
sleep_efficiency	GDS			
sleep_efficiency	QUIP			
sleep_efficiency	ESS			
sleep_efficiency	RBDSQ			

sleep_efficiency	Systolic BP Drop			
sleep_efficiency	SCOPA autonome			
sleep_efficiency	Schwab England ADL			
sleep_efficiency	UPDRS I			
sleep_efficiency	UPDRS II			
sleep_efficiency	UPDRS III OFF			
sleep_efficiency	UPDRS III ON			
sleep_efficiency	UPDRS IV			
sleep_efficiency	LEDD			
sleep_efficiency	sum_walking_minutes			
sleep_efficiency	hourly_step_count_sum			
sleep_efficiency	sleep_efficiency			
sleep_efficiency	num_awakenings	11	0.013957	0.967513
sleep_efficiency	total_sleep_time_hour	11	0.792186	0.003651
sleep_efficiency	wake_after_sleep_onset_hour	11	-0.13432	0.693759
sleep_efficiency	total_nrem_time_hour	11	0.812185	0.002389
sleep_efficiency	total_rem_time_hour	11	0.636676	0.035169
sleep_efficiency	total_deep_nrem_time_hour	11	0.493989	0.122492
sleep_efficiency	total_light_nrem_time_hour	11	0.672471	0.023385
sleep_efficiency	hourly_mean_pulse_rate	11	-0.28599	0.393911
sleep_efficiency	hourly_mean_rmssd	11	0.204106	0.547184
sleep_efficiency	hourly_median_rmssd	11	0.216408	0.522735
sleep_efficiency	hourly_rmssd_variance	11	0.024659	0.942629
num_awakenings	Semantic Fluency			
num_awakenings	MOCA			
num_awakenings	Benton			
num_awakenings	Letter Number Sequencing			
num_awakenings	HVLT Recall			
num_awakenings	HVLT Recognition			
num_awakenings	HVLT Retention			
num_awakenings	Symbol Digit			
num_awakenings	STAI trait			
num_awakenings	STAI state			
num_awakenings	GDS			
num_awakenings	QUIP			
num_awakenings	ESS			
num_awakenings	RBDSQ			

num_awakenings	Systolic BP Drop			
num_awakenings	SCOPA autonome			
num_awakenings	Schwab England ADL			
num_awakenings	UPDRS I			
num_awakenings	UPDRS II			
num_awakenings	UPDRS III OFF			
num_awakenings	UPDRS III ON			
num_awakenings	UPDRS IV			
num_awakenings	LEDD			
num_awakenings	sum_walking_minutes			
num_awakenings	hourly_step_count_sum			
num_awakenings	sleep_efficiency			
num_awakenings	num_awakenings			
num_awakenings	total_sleep_time_hour	11	0.230311	0.495675
num_awakenings	wake_after_sleep_onset_hour	11	0.947926	9.11E-06
num_awakenings	total_nrem_time_hour	11	0.254066	0.450919
num_awakenings	total_rem_time_hour	11	0.147649	0.664843
num_awakenings	total_deep_nrem_time_hour	11	-0.02574	0.940116
num_awakenings	total_light_nrem_time_hour	11	0.283646	0.397965
num_awakenings	hourly_mean_pulse_rate	11	-0.44399	0.171311
num_awakenings	hourly_mean_rmssd	11	0.168647	0.620109
num_awakenings	hourly_median_rmssd	11	0.206729	0.541933
num_awakenings	hourly_rmssd_variance	11	-0.46617	0.148386
total_sleep_time_hour	Semantic Fluency			
total_sleep_time_hour	MOCA			
total_sleep_time_hour	Benton			
total_sleep_time_hour	Letter Number Sequencing			
total_sleep_time_hour	HVLT Recall			
total_sleep_time_hour	HVLT Recognition			
total_sleep_time_hour	HVLT Retention			
total_sleep_time_hour	Symbol Digit			
total_sleep_time_hour	STAI trait			
total_sleep_time_hour	STAI state			
total_sleep_time_hour	GDS			
total_sleep_time_hour	QUIP			
total_sleep_time_hour	ESS			
total_sleep_time_hour	RBDSQ			
total_sleep_time_hour	Systolic BP Drop			

total_sleep_time_hour	SCOPA autonome			
total_sleep_time_hour	Schwab England ADL			
total_sleep_time_hour	UPDRS I			
total_sleep_time_hour	UPDRS II			
total_sleep_time_hour	UPDRS III OFF			
total_sleep_time_hour	UPDRS III ON			
total_sleep_time_hour	UPDRS IV			
total_sleep_time_hour	LEDD			
total_sleep_time_hour	sum_walking_minutes			
total_sleep_time_hour	hourly_step_count_sum			
total_sleep_time_hour	sleep_efficiency			
total_sleep_time_hour	num_awakenings			
total_sleep_time_hour	total_sleep_time_hour			
total_sleep_time_hour	wake_after_sleep_onset_hour	11	0.112228	0.742513
total_sleep_time_hour	total_nrem_time_hour	11	0.97831	1.85E-07
total_sleep_time_hour	total_rem_time_hour	11	0.901674	0.000149
total_sleep_time_hour	total_deep_nrem_time_hour	11	0.419344	0.199192
total_sleep_time_hour	total_light_nrem_time_hour	11	0.88144	0.000335
total_sleep_time_hour	hourly_mean_pulse_rate	11	-0.47231	0.142404
total_sleep_time_hour	hourly_mean_rmssd	11	0.182479	0.591248
total_sleep_time_hour	hourly_median_rmssd	11	0.195755	0.56404
total_sleep_time_hour	hourly_rmssd_variance	11	-0.40564	0.215805
wake_after_sleep_onset_hour	Semantic Fluency			
wake_after_sleep_onset_hour	MOCA			
wake_after_sleep_onset_hour	Benton			
wake_after_sleep_onset_hour	Letter Number Sequencing			
wake_after_sleep_onset_hour	HVLT Recall			
wake_after_sleep_onset_hour	HVLT Recognition			
wake_after_sleep_onset_hour	HVLT Retention			
wake_after_sleep_onset_hour	Symbol Digit			
wake_after_sleep_onset_hour	STAI trait			
wake_after_sleep_onset_hour	STAI state			

wake_after_sleep_onset_hour	GDS			
wake_after_sleep_onset_hour	QUIP			
wake_after_sleep_onset_hour	ESS			
wake_after_sleep_onset_hour	RBDSQ			
wake_after_sleep_onset_hour	Systolic BP Drop			
wake_after_sleep_onset_hour	SCOPA autonome			
wake_after_sleep_onset_hour	Schwab England ADL			
wake_after_sleep_onset_hour	UPDRS I			
wake_after_sleep_onset_hour	UPDRS II			
wake_after_sleep_onset_hour	UPDRS III OFF			
wake_after_sleep_onset_hour	UPDRS III ON			
wake_after_sleep_onset_hour	UPDRS IV			
wake_after_sleep_onset_hour	LEDD			
wake_after_sleep_onset_hour	sum_walking_minutes			
wake_after_sleep_onset_hour	hourly_step_count_sum			
wake_after_sleep_onset_hour	sleep_efficiency			
wake_after_sleep_onset_hour	num_awakenings			
wake_after_sleep_onset_hour	total_sleep_time_hour			
wake_after_sleep_onset_hour	wake_after_sleep_onset_hour			
wake_after_sleep_onset_hour	total_nrem_time_hour	11	0.175441	0.60587
wake_after_sleep_onset_hour	total_rem_time_hour	11	-0.03584	0.916673
wake_after_sleep_onset_hour	total_deep_nrem_time_hour	11	-0.107	0.754185
wake_after_sleep_onset_hour	total_light_nrem_time_hour	11	0.232139	0.492164
wake_after_sleep_onset_hour	hourly_mean_pulse_rate	11	-0.43541	0.180729
wake_after_sleep_onset_hour	hourly_mean_rmssd	11	0.227317	0.50145

wake_after_sleep_onset_hour	hourly_median_rmssd	11	0.266775	0.427779
wake_after_sleep_onset_hour	hourly_rmssd_variance	11	-0.40585	0.215544
total_nrem_time_hour	Semantic Fluency			
total_nrem_time_hour	MOCA			
total_nrem_time_hour	Benton			
total_nrem_time_hour	Letter Number Sequencing			
total_nrem_time_hour	HVLT Recall			
total_nrem_time_hour	HVLT Recognition			
total_nrem_time_hour	HVLT Retention			
total_nrem_time_hour	Symbol Digit			
total_nrem_time_hour	STAI trait			
total_nrem_time_hour	STAI state			
total_nrem_time_hour	GDS			
total_nrem_time_hour	QUIP			
total_nrem_time_hour	ESS			
total_nrem_time_hour	RBDSQ			
total_nrem_time_hour	Systolic BP Drop			
total_nrem_time_hour	SCOPA autonome			
total_nrem_time_hour	Schwab England ADL			
total_nrem_time_hour	UPDRS I			
total_nrem_time_hour	UPDRS II			
total_nrem_time_hour	UPDRS III OFF			
total_nrem_time_hour	UPDRS III ON			
total_nrem_time_hour	UPDRS IV			
total_nrem_time_hour	LEDD			
total_nrem_time_hour	sum_walking_minutes			
total_nrem_time_hour	hourly_step_count_sum			
total_nrem_time_hour	sleep_efficiency			
total_nrem_time_hour	num_awakenings			
total_nrem_time_hour	total_sleep_time_hour			
total_nrem_time_hour	wake_after_sleep_onset_hour			
total_nrem_time_hour	total_nrem_time_hour			
total_nrem_time_hour	total_rem_time_hour	11	0.792543	0.003625
total_nrem_time_hour	total_deep_nrem_time_hour	11	0.367643	0.265993
total_nrem_time_hour	total_light_nrem_time_hour	11	0.925781	4.34E-05
total_nrem_time_hour	hourly_mean_pulse_rate	11	-0.44106	0.174488
total_nrem_time_hour	hourly_mean_rmssd	11	0.221303	0.513137
total_nrem_time_hour	hourly_median_rmssd	11	0.239272	0.478569
total_nrem_time_hour	hourly_rmssd_variance	11	-0.33531	0.313436

total_rem_time_hour	Semantic Fluency			
total_rem_time_hour	MOCA			
total_rem_time_hour	Benton			
total_rem_time_hour	Letter Number Sequencing			
total_rem_time_hour	HVLT Recall			
total_rem_time_hour	HVLT Recognition			
total_rem_time_hour	HVLT Retention			
total_rem_time_hour	Symbol Digit			
total_rem_time_hour	STAI trait			
total_rem_time_hour	STAI state			
total_rem_time_hour	GDS			
total_rem_time_hour	QUIP			
total_rem_time_hour	ESS			
total_rem_time_hour	RBDSQ			
total_rem_time_hour	Systolic BP Drop			
total_rem_time_hour	SCOPA autonome			
total_rem_time_hour	Schwab England ADL			
total_rem_time_hour	UPDRS I			
total_rem_time_hour	UPDRS II			
total_rem_time_hour	UPDRS III OFF			
total_rem_time_hour	UPDRS III ON			
total_rem_time_hour	UPDRS IV			
total_rem_time_hour	LEDD			
total_rem_time_hour	sum_walking_minutes			
total_rem_time_hour	hourly_step_count_sum			
total_rem_time_hour	sleep_efficiency			
total_rem_time_hour	num_awakenings			
total_rem_time_hour	total_sleep_time_hour			
total_rem_time_hour	wake_after_sleep_onset_hour			
total_rem_time_hour	total_nrem_time_hour			
total_rem_time_hour	total_rem_time_hour			
total_rem_time_hour	total_deep_nrem_time_hour	11	0.467051	0.147518
total_rem_time_hour	total_light_nrem_time_hour	11	0.662302	0.026394
total_rem_time_hour	hourly_mean_pulse_rate	11	-0.4697	0.144925
total_rem_time_hour	hourly_mean_rmssd	11	0.07523	0.826001
total_rem_time_hour	hourly_median_rmssd	11	0.076803	0.822413
total_rem_time_hour	hourly_rmssd_variance	11	-0.49419	0.122314
total_deep_nrem_time_hour	Semantic Fluency			
total_deep_nrem_time_hour	MOCA			
total_deep_nrem_time_hour	Benton			
total_deep_nrem_time_hour	Letter Number Sequencing			

total_deep_nrem_time_hour	HVLT Recall			
total_deep_nrem_time_hour	HVLT Recognition			
total_deep_nrem_time_hour	HVLT Retention			
total_deep_nrem_time_hour	Symbol Digit			
total_deep_nrem_time_hour	STAI trait			
total_deep_nrem_time_hour	STAI state			
total_deep_nrem_time_hour	GDS			
total_deep_nrem_time_hour	QUIP			
total_deep_nrem_time_hour	ESS			
total_deep_nrem_time_hour	RBDSQ			
total_deep_nrem_time_hour	Systolic BP Drop			
total_deep_nrem_time_hour	SCOPA autonome			
total_deep_nrem_time_hour	Schwab England ADL			
total_deep_nrem_time_hour	UPDRS I			
total_deep_nrem_time_hour	UPDRS II			
total_deep_nrem_time_hour	UPDRS III OFF			
total_deep_nrem_time_hour	UPDRS III ON			
total_deep_nrem_time_hour	UPDRS IV			
total_deep_nrem_time_hour	LEDD			
total_deep_nrem_time_hour	sum_walking_minutes			
total_deep_nrem_time_hour	hourly_step_count_sum			
total_deep_nrem_time_hour	sleep_efficiency			
total_deep_nrem_time_hour	num_awakenings			
total_deep_nrem_time_hour	total_sleep_time_hour			
total_deep_nrem_time_hour	wake_after_sleep_onset_hour			
total_deep_nrem_time_hour	total_nrem_time_hour			
total_deep_nrem_time_hour	total_rem_time_hour			
total_deep_nrem_time_hour	total_deep_nrem_time_hour			
total_deep_nrem_time_hour	total_light_nrem_time_hour	11	-0.01123	0.973864
total_deep_nrem_time_hour	hourly_mean_pulse_rate	11	-0.29239	0.382932
total_deep_nrem_time_hour	hourly_mean_rmssd	11	0.263464	0.433752
total_deep_nrem_time_hour	hourly_median_rmssd	11	0.269459	0.422966
total_deep_nrem_time_hour	hourly_rmssd_variance	11	0.049252	0.885655
total_light_nrem_time_hour	Semantic Fluency			
total_light_nrem_time_hour	MOCA			
total_light_nrem_time_hour	Benton			
total_light_nrem_time_hour	Letter Number Sequencing			
total_light_nrem_time_hour	HVLT Recall			
total_light_nrem_time_hour	HVLT Recognition			
total_light_nrem_time_hour	HVLT Retention			
total_light_nrem_time_hour	Symbol Digit			
total_light_nrem_time_hour	STAI trait			

total_light_nrem_time_hour	STAI state			
total_light_nrem_time_hour	GDS			
total_light_nrem_time_hour	QUIP			
total_light_nrem_time_hour	ESS			
total_light_nrem_time_hour	RBDSQ			
total_light_nrem_time_hour	Systolic BP Drop			
total_light_nrem_time_hour	SCOPA autonome			
total_light_nrem_time_hour	Schwab England ADL			
total_light_nrem_time_hour	UPDRS I			
total_light_nrem_time_hour	UPDRS II			
total_light_nrem_time_hour	UPDRS III OFF			
total_light_nrem_time_hour	UPDRS III ON			
total_light_nrem_time_hour	UPDRS IV			
total_light_nrem_time_hour	LEDD			
total_light_nrem_time_hour	sum_walking_minutes			
total_light_nrem_time_hour	hourly_step_count_sum			
total_light_nrem_time_hour	sleep_efficiency			
total_light_nrem_time_hour	num_awakenings			
total_light_nrem_time_hour	total_sleep_time_hour			
total_light_nrem_time_hour	wake_after_sleep_onset_hour			
total_light_nrem_time_hour	total_nrem_time_hour			
total_light_nrem_time_hour	total_rem_time_hour			
total_light_nrem_time_hour	total_deep_nrem_time_hour			
total_light_nrem_time_hour	total_light_nrem_time_hour			
total_light_nrem_time_hour	hourly_mean_pulse_rate	11	-0.35538	0.283477
total_light_nrem_time_hour	hourly_mean_rmssd	11	0.130847	0.701368
total_light_nrem_time_hour	hourly_median_rmssd	11	0.14773	0.664668
total_light_nrem_time_hour	hourly_rmssd_variance	11	-0.38056	0.248247
hourly_mean_pulse_rate	Semantic Fluency			
hourly_mean_pulse_rate	MOCA			
hourly_mean_pulse_rate	Benton			
hourly_mean_pulse_rate	Letter Number Sequencing			
hourly_mean_pulse_rate	HVLT Recall			
hourly_mean_pulse_rate	HVLT Recognition			
hourly_mean_pulse_rate	HVLT Retention			
hourly_mean_pulse_rate	Symbol Digit			
hourly_mean_pulse_rate	STAI trait			
hourly_mean_pulse_rate	STAI state			
hourly_mean_pulse_rate	GDS			
hourly_mean_pulse_rate	QUIP			
hourly_mean_pulse_rate	ESS			
hourly_mean_pulse_rate	RBDSQ			
hourly_mean_pulse_rate	Systolic BP Drop			

hourly_mean_pulse_rate	SCOPA autonome			
hourly_mean_pulse_rate	Schwab England ADL			
hourly_mean_pulse_rate	UPDRS I			
hourly_mean_pulse_rate	UPDRS II			
hourly_mean_pulse_rate	UPDRS III OFF			
hourly_mean_pulse_rate	UPDRS III ON			
hourly_mean_pulse_rate	UPDRS IV			
hourly_mean_pulse_rate	LEDD			
hourly_mean_pulse_rate	sum_walking_minutes			
hourly_mean_pulse_rate	hourly_step_count_sum			
hourly_mean_pulse_rate	sleep_efficiency			
hourly_mean_pulse_rate	num_awakenings			
hourly_mean_pulse_rate	total_sleep_time_hour			
hourly_mean_pulse_rate	wake_after_sleep_onset_hour			
hourly_mean_pulse_rate	total_nrem_time_hour			
hourly_mean_pulse_rate	total_rem_time_hour			
hourly_mean_pulse_rate	total_deep_nrem_time_hour			
hourly_mean_pulse_rate	total_light_nrem_time_hour			
hourly_mean_pulse_rate	hourly_mean_pulse_rate			
hourly_mean_pulse_rate	hourly_mean_rmssd	32	-0.21969	0.226996
hourly_mean_pulse_rate	hourly_median_rmssd	32	-0.19997	0.272496
hourly_mean_pulse_rate	hourly_rmssd_variance	29	-0.18728	0.330658
hourly_mean_rmssd	Semantic Fluency			
hourly_mean_rmssd	MOCA			
hourly_mean_rmssd	Benton			
hourly_mean_rmssd	Letter Number Sequencing			
hourly_mean_rmssd	HVLT Recall			
hourly_mean_rmssd	HVLT Recognition			
hourly_mean_rmssd	HVLT Retention			
hourly_mean_rmssd	Symbol Digit			
hourly_mean_rmssd	STAI trait			
hourly_mean_rmssd	STAI state			
hourly_mean_rmssd	GDS			
hourly_mean_rmssd	QUIP			
hourly_mean_rmssd	ESS			
hourly_mean_rmssd	RBDSQ			
hourly_mean_rmssd	Systolic BP Drop			
hourly_mean_rmssd	SCOPA autonome			
hourly_mean_rmssd	Schwab England ADL			
hourly_mean_rmssd	UPDRS I			
hourly_mean_rmssd	UPDRS II			
hourly_mean_rmssd	UPDRS III OFF			
hourly_mean_rmssd	UPDRS III ON			
hourly_mean_rmssd	UPDRS IV			

hourly_mean_rmssd	LEDD			
hourly_mean_rmssd	sum_walking_minutes			
hourly_mean_rmssd	hourly_step_count_sum			
hourly_mean_rmssd	sleep_efficiency			
hourly_mean_rmssd	num_awakenings			
hourly_mean_rmssd	total_sleep_time_hour			
hourly_mean_rmssd	wake_after_sleep_onset_hour			
hourly_mean_rmssd	total_nrem_time_hour			
hourly_mean_rmssd	total_rem_time_hour			
hourly_mean_rmssd	total_deep_nrem_time_hour			
hourly_mean_rmssd	total_light_nrem_time_hour			
hourly_mean_rmssd	hourly_mean_pulse_rate			
hourly_mean_rmssd	hourly_mean_rmssd			
hourly_mean_rmssd	hourly_median_rmssd	32	0.988494	3.6E-26
hourly_mean_rmssd	hourly_rmssd_variance	29	0.486378	0.007466
hourly_median_rmssd	Semantic Fluency			
hourly_median_rmssd	MOCA			
hourly_median_rmssd	Benton			
hourly_median_rmssd	Letter Number Sequencing			
hourly_median_rmssd	HVLT Recall			
hourly_median_rmssd	HVLT Recognition			
hourly_median_rmssd	HVLT Retention			
hourly_median_rmssd	Symbol Digit			
hourly_median_rmssd	STAI trait			
hourly_median_rmssd	STAI state			
hourly_median_rmssd	GDS			
hourly_median_rmssd	QUIP			
hourly_median_rmssd	ESS			
hourly_median_rmssd	RBDSQ			
hourly_median_rmssd	Systolic BP Drop			
hourly_median_rmssd	SCOPA autonome			
hourly_median_rmssd	Schwab England ADL			
hourly_median_rmssd	UPDRS I			
hourly_median_rmssd	UPDRS II			
hourly_median_rmssd	UPDRS III OFF			
hourly_median_rmssd	UPDRS III ON			
hourly_median_rmssd	UPDRS IV			
hourly_median_rmssd	LEDD			
hourly_median_rmssd	sum_walking_minutes			
hourly_median_rmssd	hourly_step_count_sum			
hourly_median_rmssd	sleep_efficiency			
hourly_median_rmssd	num_awakenings			
hourly_median_rmssd	total_sleep_time_hour			
hourly_median_rmssd	wake_after_sleep_onset_hour			

hourly_median_rmssd	total_nrem_time_hour			
hourly_median_rmssd	total_rem_time_hour			
hourly_median_rmssd	total_deep_nrem_time_hour			
hourly_median_rmssd	total_light_nrem_time_hour			
hourly_median_rmssd	hourly_mean_pulse_rate			
hourly_median_rmssd	hourly_mean_rmssd			
hourly_median_rmssd	hourly_median_rmssd			
hourly_median_rmssd	hourly_rmssd_variance	29	0.498395	0.005929
hourly_rmssd_variance	Semantic Fluency			
hourly_rmssd_variance	MOCA			
hourly_rmssd_variance	Benton			
hourly_rmssd_variance	Letter Number Sequencing			
hourly_rmssd_variance	HVLT Recall			
hourly_rmssd_variance	HVLT Recognition			
hourly_rmssd_variance	HVLT Retention			
hourly_rmssd_variance	Symbol Digit			
hourly_rmssd_variance	STAI trait			
hourly_rmssd_variance	STAI state			
hourly_rmssd_variance	GDS			
hourly_rmssd_variance	QUIP			
hourly_rmssd_variance	ESS			
hourly_rmssd_variance	RBDSQ			
hourly_rmssd_variance	Systolic BP Drop			
hourly_rmssd_variance	SCOPA autonome			
hourly_rmssd_variance	Schwab England ADL			
hourly_rmssd_variance	UPDRS I			
hourly_rmssd_variance	UPDRS II			
hourly_rmssd_variance	UPDRS III OFF			
hourly_rmssd_variance	UPDRS III ON			
hourly_rmssd_variance	UPDRS IV			
hourly_rmssd_variance	LEDD			
hourly_rmssd_variance	sum_walking_minutes			
hourly_rmssd_variance	hourly_step_count_sum			
hourly_rmssd_variance	sleep_efficiency			
hourly_rmssd_variance	num_awakenings			
hourly_rmssd_variance	total_sleep_time_hour			
hourly_rmssd_variance	wake_after_sleep_onset_hour			
hourly_rmssd_variance	total_nrem_time_hour			
hourly_rmssd_variance	total_rem_time_hour			
hourly_rmssd_variance	total_deep_nrem_time_hour			
hourly_rmssd_variance	total_light_nrem_time_hour			
hourly_rmssd_variance	hourly_mean_pulse_rate			
hourly_rmssd_variance	hourly_mean_rmssd			
hourly_rmssd_variance	hourly_median_rmssd			
hourly_rmssd_variance	hourly_rmssd_variance			

Supplemental Table 6: Association between time progression over whole observation period

Results of the Pearson correlation analysis between the progression markers of the clinical measures and the timeseries features of the digital measures. This analysis considers the whole observation timeframe. The Pearson's r coefficient, the associated p-value, and the sample size are displayed.

clinical	digital	pearson r	p-value	N	sign
cognitive_slope_PC	PC_0	0.073888	0.397984	133	
cognitive_slope_PC	PC_1	-0.11406	0.191133	133	
cognitive_slope_PC	PC_2	-0.08416	0.335502	133	
cognitive_slope_PC	PC_3	0.071408	0.414052	133	
cognitive_slope_PC	PC_4	0.039295	0.653383	133	
cognitive_slope_PC	PC_5	0.137387	0.114806	133	
cognitive_slope_PC	PC_6	-0.00807	0.926535	133	
cognitive_slope_PC	PC_7	0.050218	0.565938	133	
cognitive_slope_PC	PC_8	0.049587	0.570839	133	
cognitive_slope_PC	PC_9	-0.02763	0.752222	133	
motor_slope_PC	PC_0	0.014569	0.867808	133	
motor_slope_PC	PC_1	0.067002	0.443516	133	
motor_slope_PC	PC_2	-0.12011	0.168494	133	
motor_slope_PC	PC_3	0.071613	0.412706	133	
motor_slope_PC	PC_4	0.159201	0.067196	133	
motor_slope_PC	PC_5	0.263544	0.002177	133	*
motor_slope_PC	PC_6	0.206735	0.016962	133	
motor_slope_PC	PC_7	0.227411	0.008477	133	*
motor_slope_PC	PC_8	-0.04705	0.590707	133	
motor_slope_PC	PC_9	-0.0459	0.599842	133	
autonome_slope_PC	PC_0	0.044563	0.607798	135	
autonome_slope_PC	PC_1	0.055712	0.521008	135	
autonome_slope_PC	PC_2	0.062071	0.474496	135	
autonome_slope_PC	PC_3	-0.10705	0.216536	135	
autonome_slope_PC	PC_4	0.099087	0.252872	135	
autonome_slope_PC	PC_5	-0.1641	0.057195	135	
autonome_slope_PC	PC_6	0.043707	0.614718	135	
autonome_slope_PC	PC_7	0.217734	0.011186	135	
autonome_slope_PC	PC_8	0.020598	0.812563	135	
autonome_slope_PC	PC_9	-0.22654	0.008239	135	*
daily_slope_PC	PC_0	-0.02267	0.794068	135	
daily_slope_PC	PC_1	0.110305	0.202807	135	
daily_slope_PC	PC_2	-0.09819	0.257217	135	

daily_slope_PC	PC_3	0.034222	0.69355	135	
daily_slope_PC	PC_4	0.257954	0.002523	135	*
daily_slope_PC	PC_5	0.173094	0.044681	135	
daily_slope_PC	PC_6	0.206027	0.016515	135	
daily_slope_PC	PC_7	0.165651	0.054848	135	
daily_slope_PC	PC_8	-0.11961	0.167041	135	
daily_slope_PC	PC_9	-0.17325	0.044487	135	
neuropsychiatric_slope_PC	PC_0	0.060311	0.487135	135	
neuropsychiatric_slope_PC	PC_1	0.051679	0.551666	135	
neuropsychiatric_slope_PC	PC_2	-0.08153	0.34719	135	
neuropsychiatric_slope_PC	PC_3	-0.00226	0.979209	135	
neuropsychiatric_slope_PC	PC_4	0.209238	0.01487	135	
neuropsychiatric_slope_PC	PC_5	0.148555	0.085514	135	
neuropsychiatric_slope_PC	PC_6	0.184589	0.032094	135	
neuropsychiatric_slope_PC	PC_7	0.07275	0.40173	135	
neuropsychiatric_slope_PC	PC_8	0.060499	0.485782	135	
neuropsychiatric_slope_PC	PC_9	-0.08387	0.333501	135	
medication_slope_PC	PC_0	-0.24964	0.004179	130	*
medication_slope_PC	PC_1	-0.00281	0.974651	130	
medication_slope_PC	PC_2	-0.01422	0.872399	130	
medication_slope_PC	PC_3	-0.12259	0.16468	130	
medication_slope_PC	PC_4	0.118107	0.180794	130	
medication_slope_PC	PC_5	-0.02314	0.793855	130	
medication_slope_PC	PC_6	-0.0268	0.762115	130	
medication_slope_PC	PC_7	0.160939	0.067369	130	
medication_slope_PC	PC_8	0.155	0.078263	130	
medication_slope_PC	PC_9	-0.05435	0.539125	130	
