# Supplementary material for: Digital outcome measures from smartwatch data relate to non-motor features of Parkinson’s disease
Source: NPJ Parkinsons Dis. 2024 May 29;10:110. doi: 10.1038/s41531-024-00719-w (PMC11137004; doi:10.1038/s41531-024-00719-w)
Supplement: Supplementary file 2 — Reporting summary [file 41531_2024_719_MOESM2_ESM.pdf]

Corresponding author(s): Cynthia Sandor

Last updated by author(s): Apr 22, 2024

Reporting Summary

Nature Portfolio wishes to improve the reproducibility of the work that we publish. This form provides structure for consistency and transparency in reporting. For further information on Nature Portfolio policies, see our Editorial Policies and the Editorial Policy Checklist.

Statistics

For all statistical analyses, confirm that the following items are present in the figure legend, table legend, main text, or Methods section.

n/a	Confirmed	
☐	☒	The exact sample size (n) for each experimental group/condition, given as a discrete number and unit of measurement
☐	☒	A statement on whether measurements were taken from distinct samples or whether the same sample was measured repeatedly
☐	☒	The statistical test(s) used AND whether they are one- or two-sided<br>*Only common tests should be described solely by name; describe more complex techniques in the Methods section.*
☐	☒	A description of all covariates tested
☐	☒	A description of any assumptions or corrections, such as tests of normality and adjustment for multiple comparisons
☐	☒	A full description of the statistical parameters including central tendency (e.g. means) or other basic estimates (e.g. regression coefficient) AND variation (e.g. standard deviation) or associated estimates of uncertainty (e.g. confidence intervals)
☐	☒	For null hypothesis testing, the test statistic (e.g. F, t, r) with confidence intervals, effect sizes, degrees of freedom and P value noted<br>*Give P values as exact values whenever suitable.*
☒	☐	For Bayesian analysis, information on the choice of priors and Markov chain Monte Carlo settings
☒	☐	For hierarchical and complex designs, identification of the appropriate level for tests and full reporting of outcomes
☐	☒	Estimates of effect sizes (e.g. Cohen's d, Pearson's r), indicating how they were calculated

Our web collection on statistics for biologists contains articles on many of the points above.

Software and code

Policy information about availability of computer code

Data collection	Data was downloaded from the PPMI Loni website (https://ida.loni.usc.edu/explore/jsp/common/login.jsp?project=PPMI&classic=true) in March 2021 with an additional download of the studywatch data in 2022. PPMI is a longitudinal, observational, multi-center natural history study which started collection in 2010.
Data analysis	All analyses were performed in python v3.9 using sklearn (Pedregosa et al., 2011) 1.2.1 for model training and evaluation, scipy 1.10.0 and pingouin (Vallat, 2018) 0.5.3 for statistical testing, and matplotlib 3.6.3 and seaborn 0.12.2 for creating figures. Data loading and manipulation has been facilitated through an adapted version of pypmi (https://github.com/rmarkello/pypmi). All code will be made available upon publication.

For manuscripts utilizing custom algorithms or software that are central to the research but not yet described in published literature, software must be made available to editors and reviewers. We strongly encourage code deposition in a community repository (e.g. GitHub). See the Nature Portfolio guidelines for submitting code & software for further information.

Data

Policy information about availability of data

All manuscripts must include a data availability statement. This statement should provide the following information, where applicable:

- Accession codes, unique identifiers, or web links for publicly available datasets
- A description of any restrictions on data availability
- For clinical datasets or third party data, please ensure that the statement adheres to our policy

All data used in this study was accessed from https://www.ppmi-info.org/access-data-specimens/download-data. The Institutional Review Board approved the PPMI program and all participants gave written informed consent. For up-to-date information on the study, visit www.ppmi-info.org.

Research involving human participants, their data, or biological material

Policy information about studies with human participants or human data. See also policy information about sex, gender (identity/presentation), and sexual orientation and race, ethnicity and racism.

Reporting on sex and gender	Male sex as reported by participants was included in the analysis as a covariate. Gender was not available and thus not included in the analysis.
Reporting on race, ethnicity, or other socially relevant groupings	We do not report race or ethnicity. We do include years of education as a proxy for social groups.
Population characteristics	The analyzed cohort consists of individuals with a diagnosis of PD who participated in the digital smartwatch study and completed clinic visits (N=149). Subanalyses were performed on different subsets of participants. 1) 85 subjects with overlapping clinic visit and digital data. 2) 35 subjects with two or more visits overlapping with digital data. 3) 139 subjects with two or more clinic visits and digital data. Digital data was collected later between 2018 and 2020.
Recruitment	24 recruitment sites in US, Europe, and Australia. At enrollment, PD subjects were 30 years or older, untreated, within two years of diagnosis, Hoehn and Yahr <3, had visually determined DaT deficiency (DaTscan). Details are described elsewhere: https://doi.org/10.1016/j.pneurobio.2011.09.005. Study protocol is available at https://www.ppmi-info.org/sites/default/files/docs/PPMI002_Clinical%20Protocol_AM3.2_30Jan2023_Final.pdf.
Ethics oversight	Institutional Review Board. Conducted in accordance with the Declaration of Helsinki and the Good Clinical Practice (GCP) guidelines after approval of the local ethics committees of the participating sites.

Note that full information on the approval of the study protocol must also be provided in the manuscript.

Field-specific reporting

Please select the one below that is the best fit for your research. If you are not sure, read the appropriate sections before making your selection.

☒ Life sciences ☐ Behavioural & social sciences ☐ Ecological, evolutionary & environmental sciences

For a reference copy of the document with all sections, see nature.com/documents/nr-reporting-summary-flat.pdf

Life sciences study design

All studies must disclose on these points even when the disclosure is negative.

Sample size	No sample size calculations were performed. Any subject with data available was included in the study. Inclusion criteria: consensus PD diagnosis, digital data collected, clinic data available for selected measures (N=149). This sample was then used for several subanalyses with further criteria.1) overlapping clinic visit and digital data (N=85), 2) at least two visits overlapping with digital data collection (N=35), 3) at least two clinic visits performed (N=139).
Data exclusions	Participants were excluded when data was missing.
Replication	Replication analyses were performed for the prediction models via nested cross-validation. No replication analysis was performed for the correlation analyses.
Randomization	For the prediction models, participants were randomly assigned to training and test sets. For other analyses, randomization did not apply as no subgroups were formed.
Blinding	Blinding was not relevant to this study as it is an analysis of observational data.

Reporting for specific materials, systems and methods

We require information from authors about some types of materials, experimental systems and methods used in many studies. Here, indicate whether each material, system or method listed is relevant to your study. If you are not sure if a list item applies to your research, read the appropriate section before selecting a response.

Materials & experimental systems

n/a	Involved in the study
☒	☐ Antibodies
☒	☐ Eukaryotic cell lines
☒	☐ Palaeontology and archaeology
☒	☐ Animals and other organisms
☒	☐ Clinical data
☒	☐ Dual use research of concern
☒	☐ Plants

Methods

n/a	Involved in the study
☒	☐ ChIP-seq
☒	☐ Flow cytometry
☒	☐ MRI-based neuroimaging

Plants

Seed stocks

Report on the source of all seed stocks or other plant material used. If applicable, state the seed stock centre and catalogue number. If plant specimens were collected from the field, describe the collection location, date and sampling procedures.

Novel plant genotypes

Describe the methods by which all novel plant genotypes were produced. This includes those generated by transgenic approaches, gene editing, chemical/radiation-based mutagenesis and hybridization. For transgenic lines, describe the transformation method, the number of independent lines analyzed and the generation upon which experiments were performed. For gene-edited lines, describe the editor used, the endogenous sequence targeted for editing, the targeting guide RNA sequence (if applicable) and how the editor was applied.

Authentication

Describe any authentication procedures for each seed stock used or novel genotype generated. Describe any experiments used to assess the effect of a mutation and, where applicable, how potential secondary effects (e.g. second site T-DNA insertions, mosiacism, off-target gene editing) were examined.
